# A Striking Mode of Activation of Carbon Disulfide with a Cooperative Bis(silylene)

**DOI:** 10.1002/anie.202110398

**Published:** 2021-11-25

**Authors:** Marcel‐Philip Luecke, Luisa Giarrana, Arseni Kostenko, Tobias Gensch, Shenglai Yao, Matthias Driess

**Affiliations:** ^1^ Department of Chemistry: Metalorganics and Inorganic Materials Technische Universität Berlin Strasse des 17. Juni 115, Sekr. C2 10623 Berlin Germany

**Keywords:** dearomatization, silenes, silicon, small-molecule activation

## Abstract

The reactivity of the 1,4‐substituted bis(silylenyl)terphenylene **1**, 1,4‐[*ortho*‐(LSi)C_6_H_4_]_2_C_6_H_4_, (L=RC(NtBu)_2_, R=Ph, Mes) towards CS_2_ is reported. It results in a dearomatization of the phenylene ring, affording the 1,3‐substituted cyclohexadiene derivative **2**. According to DFT calculations, a transient silene containing a Si=C bond capable of π(C=C) addition at the aromatic phenylene ring is a key intermediate. In contrast, addition of CS_2_ to the biphenyl‐substituted mono‐silylene *ortho*‐(LSi)C_6_H_4_‐C_6_H_5_ **3** leaves the aromatic π‐system intact and forms, in a [1+2] cycloaddition reaction, the corresponding thiasilirane **4** with a three‐membered SiSC ring. Further experimental studies led to the isolation of the novel mesoionic five‐membered Si_2_S_2_C heterocycle **6**, which reacts with CS_2_ under C−C bond formation. All isolated new compounds were fully characterized and their molecular structures determined by single‐crystal X‐ray diffraction analyses.

## Graphical abstract

An unexpected new type of cooperativity of the two silicon(II) atoms in a 1,4‐terphenyl‐bridged bis(silylene) towards CS_2_ is reported and the results are compared with that of related mono‐silylenes. While a dearomatization occurs with the bis‐silylene, a novel mesoionic five‐membered Si_2_S_2_C heterocyclic compound, among other new products, results from stepwise degradation of CS_2_ with mono‐silylenes.

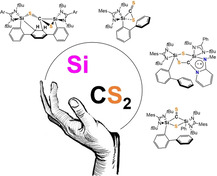

## Introduction

**Figure 1:**
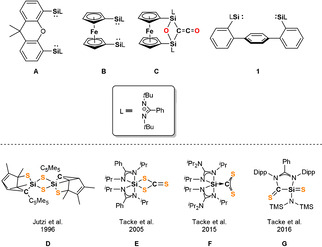
Top: Bis(NHSis) **A**, **B**, **1** and the disilaketene **C**; bottom: known products **D**–**G** resulting from CS_2_ activation with NHSis.

The activation of small molecules offers numerous possibilities to synthesize functional organic compounds. While the activation of small molecules used to be a domain of transition‐metals (TMs), during the last decade, it has been shown that even unactivated substrates such as H_2_, CO and N_2_ can be transformed without the mediation of TMs, representing a major achievement in main‐group chemistry.^[1]^ Especially low‐valent main‐group element compounds such as silylenes were found to be capable of activating numerous simple substrates such as O_2_, N_2_O, S_8_, P_4_, NH_3_, CO_2_, H_2_ and CO.[^2^, ^3^] Since the first report of an isolable *N*‐heterocyclic silylene (NHSi) by West and Denk in 1994,^[4]^ a wide range of cyclic, acyclic and base‐stabilized silylenes were synthesized.[^2^, ^5^] Featuring a lone‐pair of electrons and a vacant 3p‐orbital at the silicon center, silylenes are known to exhibit a Lewis amphoteric behavior in which both the Lewis acidic as well as the Lewis basic centers are located at the Si^II^ atom. Bridged bis(N‐heterocyclic silylenes) [bis(NHSis)] with two silylene moieties present in a single molecule were shown to act as very electron‐rich donors in metal‐mediated homogeneous catalysis and in metal‐free cooperative activation of small molecules (Figure 1, top). Furthermore, bis(NHSis) are suitable to stabilize unusually low oxidation states of main‐group elements such as zero‐valent single silicon and germanium atoms in silylones and germylones, respectively.^[6]^

Very recently, we reported the isolation of a bis(NHSi)‐stabilized diphosphorus compound acting as P^−^ transfer reagent and the cooperative activation of CO by the bis(NHSis) **A** and **B** leading to disilaketenes such as **C**, which react with NH_3_ or primary amines to afford acetamides.^[7]^ For the latter, it turned out that cooperativity of the bis(NHSis) is key to bring about unprecedented activation modes of CO, resulting in a bis(NHSi)‐μ(CO) complex in the first step of activation.^[8]^ Activation of CO_2_ with mono‐silylenes was intensively studied^[9]^ and very recently, cooperativity of bis(NHSis) towards activation of CO_2_ could also be observed for **A** yielding a cyclic bis(silylated) carbonate.^[10b]^ Hereby, depending on the hybridization/stabilization of the Si atom, elusive silanones are formed as transient intermediates which undergo fast dimerization to cyclic disiloxanes or formation of silyl carbonates from the reaction with an additional CO_2_ molecule.[^9^, ^10a^]

CS_2_, a heavy chalcogen homologue of CO_2_, is another versatile building block. It is used for the industrial production of viscose fibers from cellulose and as precursor for the synthesis of CCl_4_ and thiourea in large quantities.^[11]^ Furthermore, CS_2_ is employed as a convenient C_1_ building block for C,S‐containing compounds in organic chemistry.[^11^, ^12^] With respect to the reactivity of low‐valent silicon compounds towards CS_2,_ Jutzi discovered the formation of a dithiasilethane **D** from the reaction of decamethylsilicocene and CS_2_.^[13]^ More recently, the Tacke group showed that mono‐NHSis lead to the isolable NHSi→CS_2_ adduct **F** as a proposed key intermediate for a variety of sila‐C,S‐containing heterocycles **D**, **E** and **G**.[^14^, ^15^, ^16^] We wondered whether the two silylene groups in bis(silylene)terphenylene **1** are cooperative in the activation of CS_2_.

**Scheme 1:**
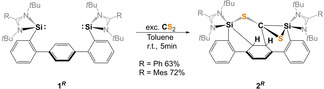
Reaction of bis(NHSis) **1** ^***R***^ with CS_2_ to afford the de‐aromatized products **2** ^***R***^ (R=Ph, Mes).

**Scheme 2:**
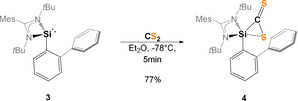
Synthesis of the thiasilirane **4**.

**Scheme 3:**
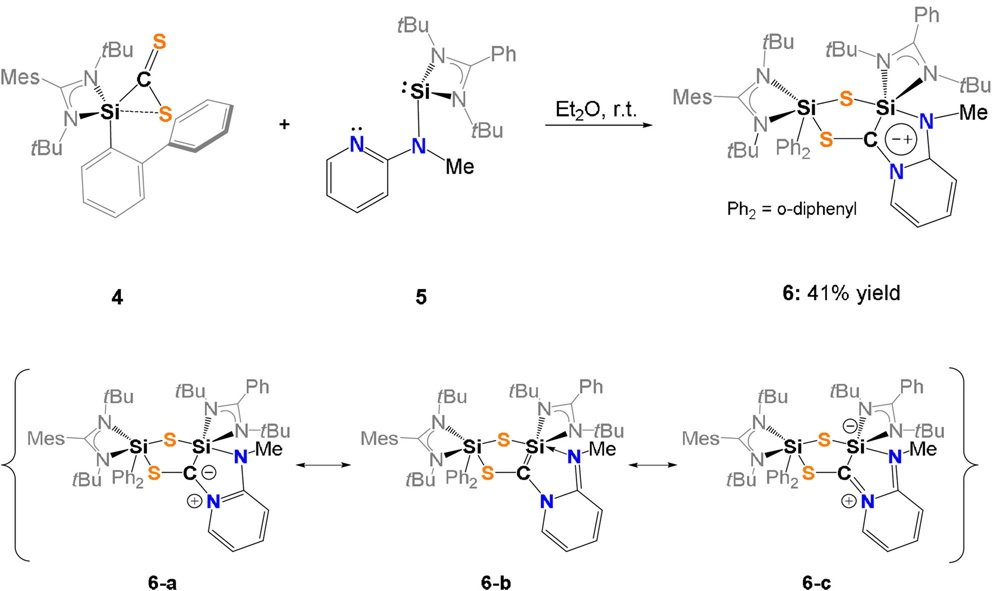
Synthesis of the mesoionic compound **6** (top) with the representative resonance structures **6 a**–**c** (bottom).

Herein, we report the cooperativity of the two silylene groups in **1** towards CS_2_, affording the unusual de‐aromatization product **2** via intramolecular cycloaddition of a reactive silene intermediate to the phenylene ring (Scheme 1). Furthermore, we invesitigated the reactivity of the new biphenyl‐substituted thiasilirane **4**, synthesized from the corresponding mono‐silylene **3** and CS_2_ (Scheme 2) and its transformation to the novel mesoionic five‐membered Si_2_S_2_C heterocyclic compound **6** (Scheme 3).

## Results and Discussion

Exposure of bis(NHSis) **1** ^***Ph***^ and **1** ^***Mes***^ in toluene to CS_2_ at room temperature led to the selective formation of the highly asymmetric coupling products **2** ^***Ph***^ and **2** ^***Mes***^, which could be isolated in 63 and 72 % yields as yellow solids, respectively (Scheme 1).

The composition and connectivity of the products was confirmed by HR‐ESI mass spectrometry, multinuclear NMR spectroscopy and, in the case of **2** ^***Mes***^, by a single crystal X‐ray structure analysis, revealing the unexpected de‐aromatization of the phenylene ring. However, suitable single crystals of **2** ^***Ph***^ could not be obtained.

As expected, the ^29^Si NMR spectrum of **2** ^***Ph***^ exhibits two singlets at *δ*=−114.3 (Si1) and −35.7 ppm (Si2) which were assigned by means of a ^1^H,^29^Si‐HMQC NMR spectrum. The formation of **2** ^***Ph***^ is further supported by the appearance of two new high‐field shifted ^13^C resonances in the ^13^C‐NMR spectra at *δ*=35.7 and 43.7 ppm corresponding to the C(sp^3^) carbons of the cyclohexa‐1,3‐diene unit in **2** ^***Ph***^. The related ^1^H resonances appear at *δ*=3.11 (d, ^3^ *J*_H,H_=11.0 Hz) and 2.23 ppm (dd, ^3^ *J*_H,H_=11.0 Hz, ^4^ *J*_H,H_=3.3 Hz) in the ^1^H‐NMR spectrum. The C(sp^2^)‐H protons resonate at *δ*=6.60 (dd, ^3^ *J*_H,H_=5.8 Hz, ^4^ *J*_H,H_=3.3 Hz) and *δ*=6.36 ppm (d, ^3^ *J*_H,H_=5.8 Hz) with coupling to ^13^C(sp^2^) nuclei as shown in the ^1^H,^13^C‐HSQC NMR spectrum at δ(^13^C)=115.1 and 123.8 ppm. The chiral, quaternary C1 nucleus of **2** ^***Ph***^ appears in the ^13^C‐NMR spectrum at *δ*=43.9 ppm (**2** ^***Mes***^: 42.1 ppm) which could be assigned with ^13^C‐DEPT and ^1^H,^13^C‐HMBC NMR spectra. The structural similarity of **2** ^***Ph***^ and **2** ^***Mes***^ is given based on consistent NMR spectroscopic and HR‐ESI mass spectrometric features (see Supporting Information).

**Figure 2:**
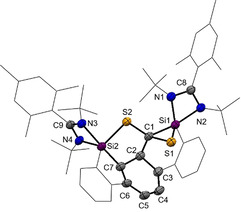
Molecular structure of **2** ^***Mes***^ at 50 % probability level.^[39]^ Hydrogen and solvent atoms are omitted for clarity. Bond lengths [Å]: C1–C2 1.543(5), C2–C7 1.524(4), C3–C4 1.356(5), C1–S1 1.908(3), C1–S2 1.795(3), Si1–C1 1.883(3), Si1–S1 2.1455(12), Si2–S2 2.1817(11), Si2–C7 1.924(3), C7–C6 1.513(4), C6–C5 1.349(5), C5–C4 1.446(5), C4–C3 1.356(5), C3–C2 1.512(4). Bond angles [°]: S2‐C1‐S1 112.65(17), C2‐C1‐S2 110.5(2), C2‐C1‐S1 116.8(2), C2‐C1‐Si1 115.6(3), Si1‐C1‐S1 68.92(11), S2‐Si2‐C7 91.47(10), S2‐Si2‐N4 113.62(9), S2‐Si2‐N3 86.64(8).

Single‐crystals suitable for an X‐ray diffraction analysis of **2** ^***Mes***^ were obtained in concentrated toluene solutions at room temperature (Figure 2). The compound crystallizes as yellow rods in the triclinic space group $P\bar{1}$ with one molecule **2** ^***Mes***^ and two toluene molecules in the asymmetric unit cell. Compound **2** ^***Mes***^ has a highly unsymmetrical structure containing a cyclohexa‐1,3‐diene unit. It features a new C−C and Si−C single bond with the C1‐C2 distance of 1.543(5) Å and Si2‐C7 distance of 1.924(3) Å, respectively. In line with the presence of a cyclohexa‐1,3‐diene, the C3‐C4 (1.356(5) Å) and C5‐C6 (1.349(5) Å) bond distances are much shorter compared to the C2‐C7 bond distance with 1.524(4) Å for a C−C single bond. The Si‐S bond distances of d(Si1‐S1)=2.1455(12) Å and d(Si2‐S2)=2.1817(11) Å are both in the range of a Si‐S single bond.[^17^, ^18^] The obtained Si−S/C−S and Si−C bond distances of the silylcyclopentene and silacyclohexene units are in accordance with the reported bond distances from the literature.[^13^, ^14^, ^15^, ^16^] The formation of a 1‐thia‐2‐silacyclopentane unit in **D** (Figure 1) was also ascribed by Jutzi et al. starting from decamethylsilicocene and CS_2_.^[13]^ In contrast to the isolated dithiasilethane **E** and silathione **I**, no C‐S bond cleavage did occur.

**Figure 3:**
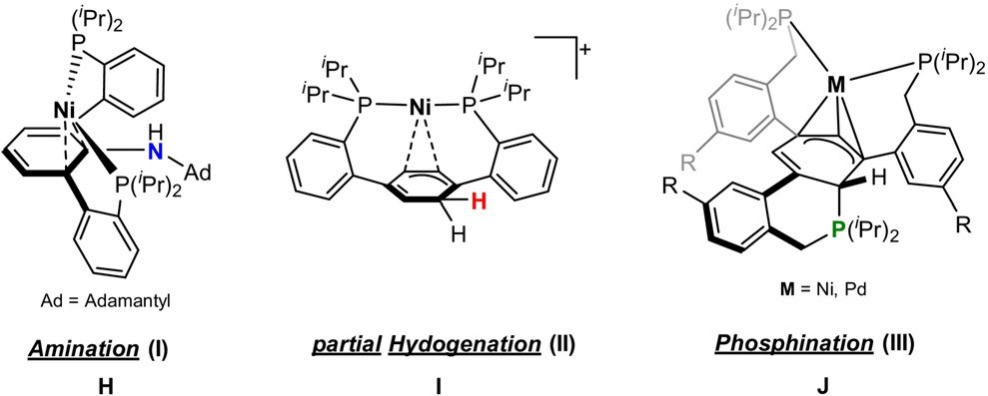
Reactivity of terphenyl‐based metal complexes.

De‐aromatization is a fundamental process in organic and metalorganic chemistry allowing further functionalization of aromatic compounds.^[19]^ In comparison to heteroatom containing cyclic arenes (e.g., thiophene, pyridine), benzene exhibits the highest resonance energy of 36 kcal mol^−1^.^[20]^ Based on terphenyl‐supported bis(phosphine)nickel complexes, Agapie et al. reported on the amination^[21]^ and partial hydrogenation of the central phenylene unit (Figure 3).^[22]^ Similarly, the phosphination based on a tris(phosphine)metal complex (M=Ni, Pd) was also reported by this group.^[23]^ However, a metal‐free, silicon‐based de‐aromatization yielding a cyclohexa‐1,3‐diene is unprecedented.

**Figure 4:**
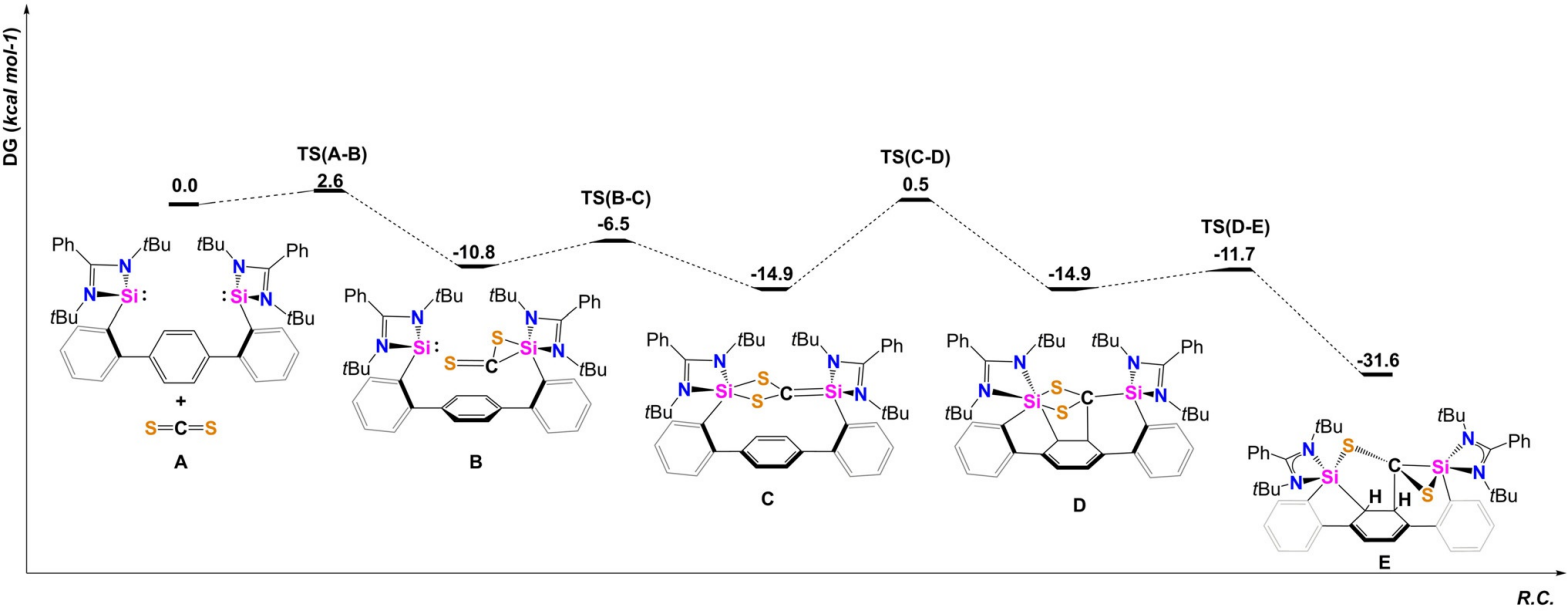
Calculated potential energy surface (PES) of the proposed mechanism starting from bis(NHSi) **1** and CS_2_.

To further elucidate the mechanism leading to this unexpected de‐aromatization at room temperature, affording compound **2**, Density Functional Theory (DFT) calculations were performed suggesting the formation of a reactive silene as key intermediate.^[24]^ The DFT‐derived mechanism (Figure 4) leading to **2** ^***Ph***^ includes the initial formation of a NHSi→CS_2_ adduct **B** from which the reactive silene intermediate **C** (Δ*G*=−14.9 kcal mol^−1^) is formed by an intramolecular attack of the second Si^II^‐unit via **TS(B‐C)** at Δ*G* ^≠^=−6.5 kcal mol^−1^. The silene intermediate **C** is capable to add to one C=C bond of the 1,4‐terphenyl spacer in a single step via the low barrier **TS(C‐D)** of Δ*G* ^≠^=0.5 kcal mol^−1^. Silenes (R_2_Si=CR_2_) are very reactive species which could only be observed as transient intermediates until 1981 when Brook et al. successfully isolated and characterized the first isolable silene.^[25]^ Silene intermediates are known to play an important role in organosilicon synthesis and are prone to undergo [2+2] cycloaddition reactions.^[26]^ After isomerization of the Si‐S bond, the final product **2** ^***Ph***^ is formed, which can be seen as an intramolecularly trapped silene capable to de‐aromatize the bridging phenylene. With Δ*G*=−31.6 kcal mol^−1^, the overall reaction (**A** → **E**) is strongly exergonic and thus in accordance with experimental findings in which a selective formation of **2** ^***Ph***^ was observed within the first minute at room temperature.

**Figure 5:**
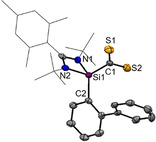
Molecular structure of **4** at 50 % probability level.^[39]^ Hydrogen and solvent atoms are omitted for clarity. Bond lengths [Å]: Si1–C1 1.855(7), Si1–C2 1.871(6), Si1⋅⋅⋅S1 3.133(3), Si1⋅⋅⋅S2 2.707(3), C1–S1 1.657(7), C1–S2 1.667(7). Bond angle [°]: N1‐Si1‐C1 111.0(3), N2‐Si1‐C1 114.4(3), N1‐Si1‐C2 112.6(3), N2‐Si1‐C2 110.8(3), C1‐Si1‐C2 123.9(3), S1‐C1‐Si1 129.3(4), S2‐C1‐Si1 100.1(3).

**Figure 6:**
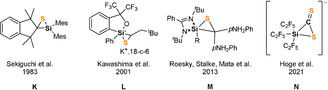
Known thiasilirane derivatives.

Intrigued by the striking reactivity of **1** towards CS_2_, we synthesized the new biphenyl‐substituted NHSi **3** to investigate whether the aromatic π‐system in **3** is similarly attacked. However, this transformation led solely to the thiasilirane **4** (Scheme 2), which could be isolated as red rods in 77 % yields. Single‐crystals suitable for an X‐ray diffraction analysis were obtained in concentrated toluene solutions. Compound **4** crystallizes in the monoclinic space group *P*c with two independent molecules in the unit cell. Its molecular structure (Figure 5) contains a three‐membered SiCS‐ring as central structural motif with a Si−C bond distance of 1.855(6) Å, which is in accordance with the reported Si−C bond distance of 1.843 Å in the thiasilirane **N** reported by Hoge et al.^[27]^ and the typical Si‐C distance of about 1.86 Å.^[28]^ However, the Si−C bond distance in **4** is shorter compared to that in the NHSi→CS_2_ adduct **F** (see Figure 1) reported by Tacke et al. (*d*(Si‐C)=1.865 Å).^[15]^ Notably, the Si1⋅⋅⋅S2 distance of 2.707 Å is significantly longer than those in the related thiasiliranes **K**–**N** (see Figure 6) (2.09–2.21 Å)[^27^, ^29^, ^30^, ^31^] and typical Si‐S bond distances (2.13–2.15 Å).^[32]^

However, the Si1‐S2 distance of **4** is similar to that in the anionic, pentacoordinated thiasiliranide **N** (*d*(Si‐S)=2.660 Å),^[27]^ suggesting a weak S2→Si1 bonding interaction. The C‐S bond distances in **4** (1.657(7) and 1.667(7) Å) are in close agreement with those in **N** with *d*(C‐S)=1.658(7) Å and 1.684(6) Å, respectively.

The C1 atom of **4** appears as a singlet resonance in the ^13^C‐NMR spectrum at *δ*=274.8 ppm which is strongly low‐field shifted compared to CS_2_ (δ(^13^C)=192.4 ppm) and the zwitterionic carbene‐ and phosphine→CS_2_ adducts (*δ*(^13^C)=219–222 ppm).^[33]^ Tacke et al. reported for the chemically closer related NHSi→CS_2_ adduct **F** a similar chemical shift for the C1 nucleus at *δ*(^13^C)=253 ppm.^[15]^ In accordance with the presence of a pentacoordinate silicon atom, the ^29^Si nucleus in **4** is strongly low‐field shifted (*δ*(^29^Si)=−89.2 ppm) compared to NHSi **3** (*δ*(^29^Si)=17.1 ppm). Similar low‐field shifts were observed for the thiasilirane derivatives **K**–**N** (Figure 6) of *δ*(^29^Si)=−70 to −118 ppm. Noteworthy, the ^13^C nucleus of the amidinato backbone in **4** resonates at lower field with *δ*=182 ppm (**3**: *δ*(^13^C)=157 ppm), implying a considerable zwitterionic electronic structure. Similarly, both Si−N bonds are shortened, suggesting that **4** is best described as a pentacoordinate, neutral thiasilirane with a relatively weak Si1‐S2 bonding interaction (3.133(3) Å) akin to that in **N**. Noteworthy, **4** is not stable in solutions and slowly converts to a silathione as indicated by a color change from dark pink to deep brown upon release of CS. The formation of the silathione is further supported by heteroatom NMR spectroscopy and an X‐ray diffraction analysis (see Supporting Information). Similarly, Tacke et al. reported the isolation of the silylated trithiocarbonate **E** (Figure 1), proposing the release of CS from a hexacoordinated NHSi→CS_2_ adduct.^[15]^

With compound **4** in hand, we wondered whether the postulated silene can be trapped upon reaction with NHSi **5** containing a (methylamino)pyridine group with an additional intramolecular *N*‐donor site.^[34]^ However, we learned that **4** acts as a C,S‐transfer reagent,[^15^, ^27^, ^29^, ^30^, ^31^] affording the first mesoionic heterobicyclic species of this type, compound **6**, which could be isolated in 41 % yields as orange crystals from concentrated Et_2_O solutions at −30 °C (Scheme 3).^[35]^ In‐situ NMR analysis of the reaction mixture revealed the selective formation of **6** within minutes as indicated by a color change from dark red to yellow.

**Figure 7:**
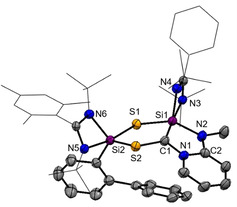
Molecular structures of **6** at 50 % probability level.^[39]^ Hydrogen and solvent atoms are omitted for clarity. Bond lengths [Å]: C1–Si1 1.844(3), C1–S2 1.747(3), Si1–S1 2.1804(11), Si1–S1 2.1804(11), Si2–S1 2.2023(10), Si2–S2 2.1767(10). Bond angle [°]: S2‐C1‐Si1 127.70(17), C1‐S2‐Si2 104.37(10), Si1‐S1‐Si2 108.94(4), S1‐Si2‐S2 98.20(4), C1‐Si1‐S1 95.27(10).

The ^29^Si NMR spectrum of **6** recorded at −50 °C exhibits two singlet resonances at high field at *δ*=−71.3 (Si2) and −73.4 ppm (Si1) which could be assigned with a ^1^H,^29^Si‐HMQC NMR spectrum. The observed high‐field shifted resonances are in line with the presence of two penta‐coordinated ^29^Si atoms, as observed in the molecular structure depicted in Figure 7.

**6** crystallizes in the monoclinic space group *P*2_1_/*n* with one molecule of Et_2_O in the unit cell. The central structural motif shows a planar CSi_2_S_2_‐dithiadisilolane ring. The C1 atom is trigonal planar (∑=359.09°) surrounded as expected for a sp^2^‐hybridized C‐atom (Figure 7). The Si1‐C1 bond distance of 1.844(3) Å is in the range of a typical Si‐C single bond (1.85–1.89 Å).^[28]^ The Si‐S distances in **6** (see Figure 7) are akin to common Si‐S bonds (2.13–2.15 Å).^[32]^ The C1‐N1 (1.360(4) Å) and C2‐N2 bond lengths (1.372(2) Å) are in‐between typical C‐N‐ (1.45–1.48 Å) and C=N‐bonds (1.28–1.31 Å).^[28]^ Comparison of the N1‐C1, N1‐C5 and C3‐C4 distances within the pyridine unit of NHSi **5** indicates that the π‐electrons are delocalized over the diazasilolidine ring (see Supporting Information), which also explains its planarity.

As mentioned above, **6** is a mesoionic heterocycle, which includes a ylidic C−N bond (see resonance structure **6 a**, Scheme 3). Other resonance structures include the silylated imine **6 b** and a silene form **6 c**, respectively. The ylide‐like structure of **6** is further supported by a NBO analysis (Supporting Information) in which the LUMO is represented by the π*(C‐N) orbital.[^27^, ^36^] Additional theoretical calculations revealed that the formation of the proposed silene‐intermediate found at Δ*G*=30.1 kcal mol^−1^ is most unlikely to occur.

**Scheme 4:**
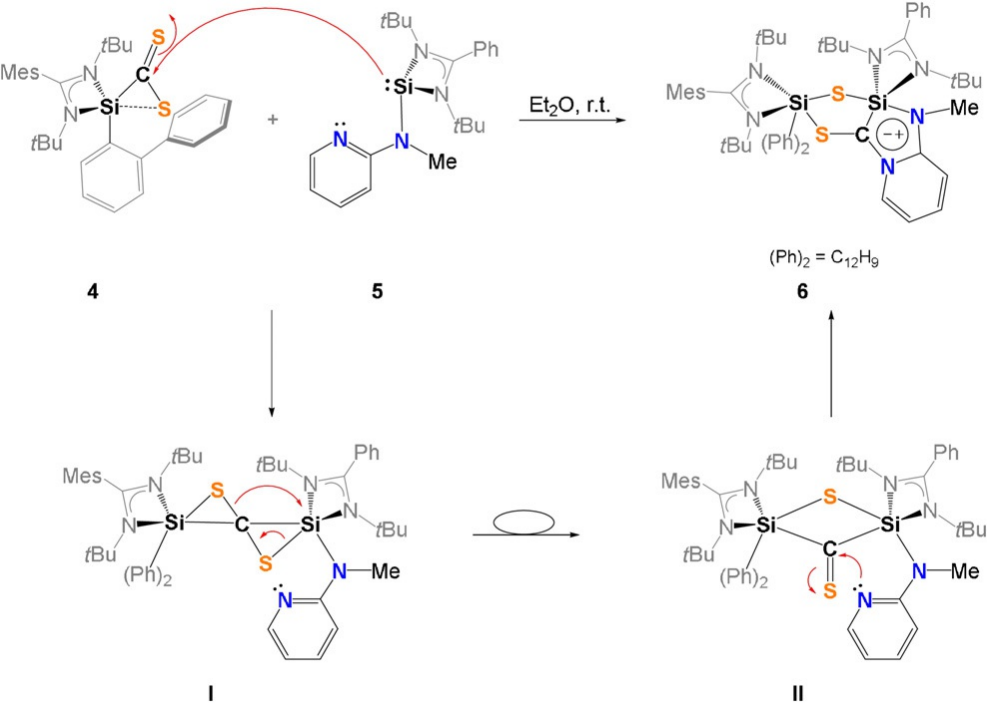
Proposed mechanism for the formation of **6**.

The proposed mechanism for the formation of **6** (Scheme 4) involves the formation of the 3,3′‐spirobi(1,2‐thiasilirane) intermediate **I** as previously suggested by Okazaki et al. via Si−C bond formation.^[37]^ **I** rearranges to a bis(silyl)thioketone **II** containing a four‐membered Si‐S‐Si‐C ring. A comparable species was isolated by Okazaki et al. in 1996.^[37]^ Nucleophilic attack of the intramolecular *N*‐donor atom of the pyridine unit in **II** initiates the final rearrangement to furnish the final product **6**.

**Scheme 5:**
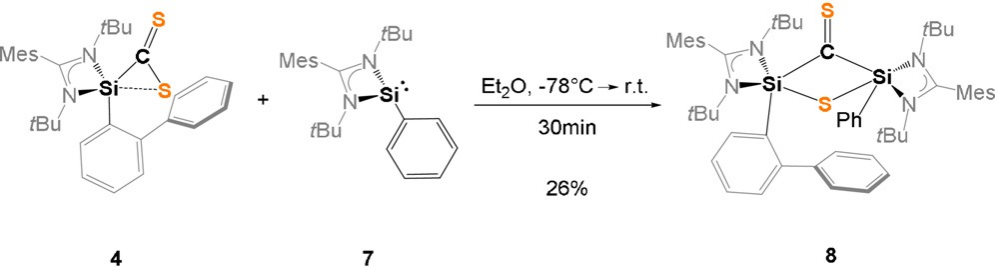
Synthesis of the bis(silylated) thione **8**.

Interestingly, in the absence of an additional *N*‐donor site, the proposed intermediate **II**, the bis(silylated) thione **8** could be isolated in 26 % yields as pale green crystals from concentrated Et_2_O solutions at −30 °C (Scheme 5).

**Figure 8:**
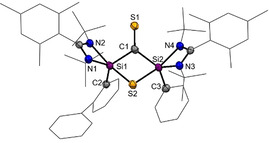
Molecular structure of **8** at 50 % probability level.^[39]^ Hydrogen and solvent atoms are omitted for clarity. Bond lengths [Å]: Si1–C1 1.8885(19), Si2–C1 1.905(2), C1–S1 1.654(2), Si1–S2 2.2652(7), Si2–S2 2.2760(6). Bond angle [°]: Si1‐S2‐Si2 86.62(2), Si1‐C1‐Si2 110.42(10), C1‐Si1‐S2 74.63(6), C1‐Si2‐S2 74.08(6), S1‐C1‐Si1 119.35(11), S1‐C1‐Si2 122.07(11).

Single‐crystals of **8** were obtained from the reaction mixture in C_6_D_6_ at room temperature. The compound crystallizes in the triclinic space group $P\bar{1}$ with three molecules of benzene in the asymmetric unit cell. The central structural motif is based on a non‐planar, four‐membered Si_2_SC‐ring with two penta‐coordinate Si atoms (Figure 8).

The FT‐IR spectrum of **8** shows a characteristic strong absorption band in the $\tilde{\nu }$ (C=S) range of 1230–1082 cm^−1^ for thiones.^[38]^ Both Si atoms show a similar ^29^Si chemical shift of *δ*=−59.7 (Si2) and −67.4 ppm (Si1). The ^13^C nucleus of the C=S group appears as a singlet at *δ*(^13^C)=267.3 ppm.

**Scheme 6:**
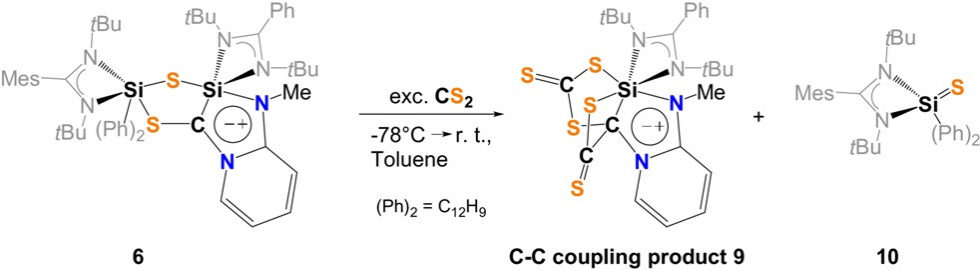
Synthesis of the C−C‐coupling product **9** and liberation of **10**.

Next, the reactivity of **6** and **8** towards CS_2_ was examined to address the question whether C−C bond formation does occur. While bis(silyl)thione **8** showed no reactivity, addition of CS_2_ to **6** in toluene at room temperature resulted in the immediate color change from orange to pink. After one week, red single‐crystals could be obtained in small quantities which turned out to be the trithiasilabicyclo[3.2.0]‐dithione **9**, resulting from the fragmentation reaction of **6** with two molar equivalents of CS_2_ (Scheme 6).

**Figure 9:**
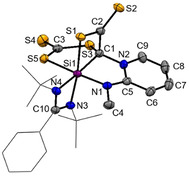
Molecular structure of **9** at 50 % probability level.^[39]^ Hydrogen and solvent atoms are omitted for clarity. Bond lengths [Å]: C1–C2 1.544(2), C1–Si1 1.9639(18), C2–S2 1.6426(18), C2–S1 1.7078(18), C3–S4 1.6548(19), Si1–S1 2.3340(6), Si1–S5 2.3521(6), C1–S3 1.8058(18), C3–S3 1.7609(19), C3–S5 1.7005(19). Bond angle [°]: C1‐C2‐S1 106.79(12), S3‐C3‐S5 120.81(11), C1‐Si1‐S5 88.98(5), S1‐Si1‐S5 90.53(2), C1‐Si1‐S1 74.31(5).

**9** crystallizes in the triclinic space group $P\bar{1}$ with one molecule of toluene in the asymmetric unit cell (Figure 9). The addition of one CS_2_ molecule occurs along the Si−C bond and can be described as a [2+2]‐cycloaddition product of a silene and CS_2_ in the first step, resulting in a four‐membered SiSC_2_‐ ring in **9**. Similarly to **6**, compound **9** has a mesoionic structure containing a octahedrally coordinated Si1 atom with a chemical shift of δ(^29^Si)=−143.4 ppm in solution. In‐situ NMR analysis of the reaction mixture indicated the formation of the corresponding silathione **10** as the only byproduct (δ(^29^Si)=4.4 ppm).

The Si1‐C1 bond (1.9639(18) Å) is elongated compared to typical Si−C bonds with *d*(Si‐C) distances of 1.85–1.89 Å.^[28]^ The C1‐C2 distance of 1.544(2) Å represents a single bond. The two Si‐S bonds amount 2.3340(6) Å (Si1‐S1) and 2.3521(6) Å (Si1‐S5), which are both elongated to those observed in **6** (*d*(Si‐S)=2.17/2.20 Å) and a common Si‐S single bond (*d*(Si‐S)=2.13–2.15 Å),[^17^, ^18^] but shorter if compared to the isolated thiasilirane **4** (2.707) Å).

**Scheme 7:**
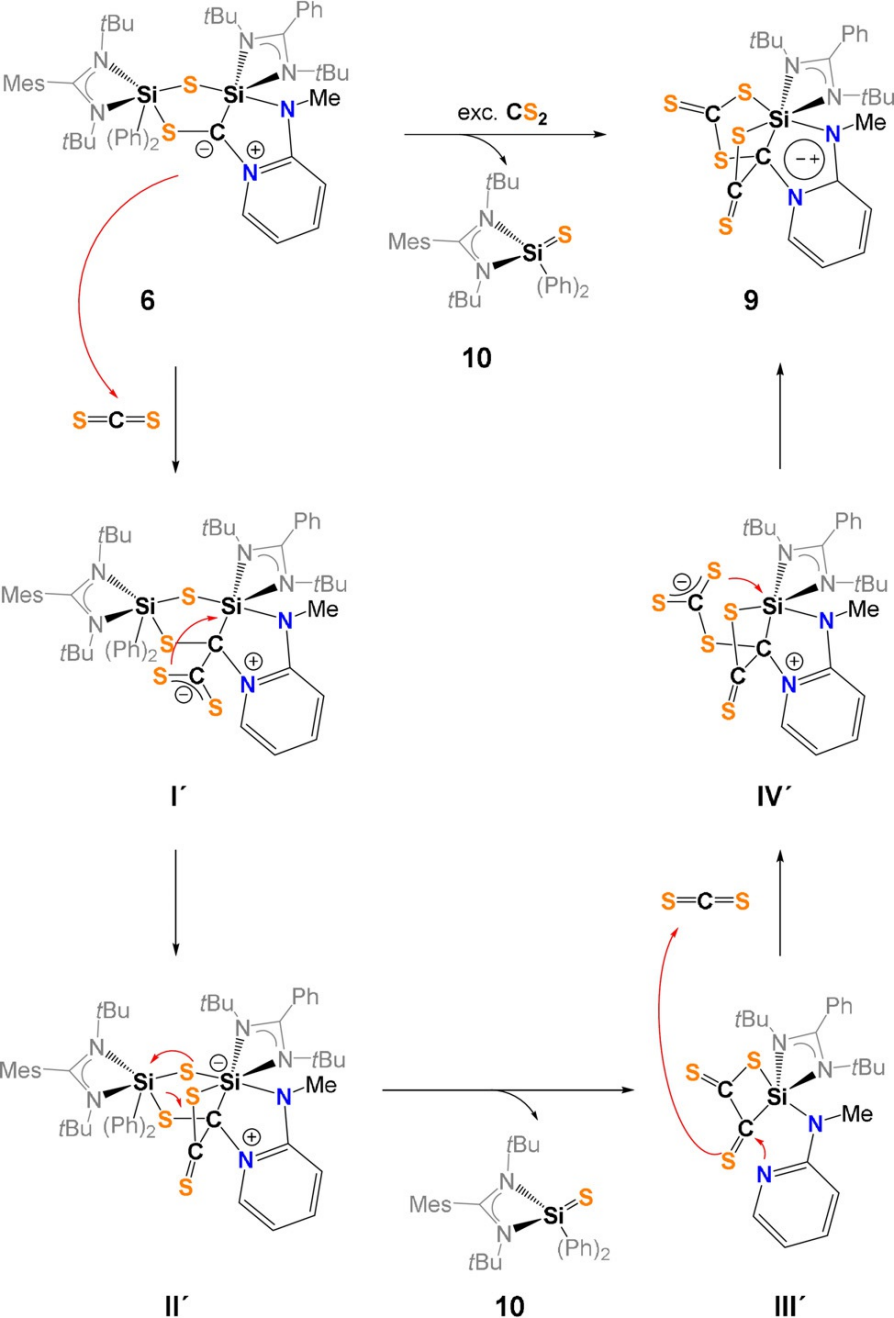
Proposed mechanism for the formation of **9** and **10**.

Based on the observation of silathione **10** as stoichiometric byproduct in the reaction mixture, the mechanism depicted in Scheme 7 is proposed for the formation of the C−C‐coupling product **9**.

In the first step, a CS_2_ molecule forms the adduct **I′** with the partially negatively charged C atom in **6**. Next, the four‐membered SiCCS‐ring is formed via S−Si bond formation. Under liberation of the silathione **10**, the formation of dithione **II′** is proposed, containing two C=S moieties along with C‐N(pyridine) bond breaking. Through a nucleophilic attack of the N‐pyridine atom in **III′**, the reaction of a second CS_2_ molecule takes place with formation of a S−C and S−Si bond; in the last step, the final product **9** is formed.

## Conclusion

In summary, the new mode of activation of CS_2_ by a 1,4‐terphenyl supported bis(NHSi) **1** was reported, resulting in the room temperature de‐aromatization of an intramolecular bridging phenylene ring to afford **2**. DFT calculations revealed a reactive base‐stabilized silene **C** as key intermediate for this facile transformation. In contrast, the reaction of the diphenyl‐substituted mono‐NHSi **3** and CS_2_ resulted in the isolation of an unstable thiasilirane **4** which slowly converts to a silathione upon release of CS. The reaction of the NHSi **5** containing a (methylamino)pyridine substituent furnished the novel mesoionic compound **6** which does further react with CS_2_ under C−C bond formation similar to the reaction of bis(NHSi) **1** and CS_2_. The series of different CS_2_ activation products with divalent Si demonstrates the high potential of mono‐ and bis‐silylenes in generating multifunctional organosulfur species which are difficult to access by other routes.

## Conflict of interest

The authors declare no conflict of interest.

## Supporting information

As a service to our authors and readers, this journal provides supporting information supplied by the authors. Such materials are peer reviewed and may be re‐organized for online delivery, but are not copy‐edited or typeset. Technical support issues arising from supporting information (other than missing files) should be addressed to the authors.

Supporting Information
