# Supplementary material for: A Striking Mode of Activation of Carbon Disulfide with a Cooperative Bis(silylene)
Source: Angew Chem Int Ed Engl. 2021 Nov 25;61(2):e202110398. doi: 10.1002/anie.202110398 (PMC9298776; doi:10.1002/anie.202110398)
Supplement: Supplementary file 1 — Supporting Information [file ANIE-61-0-s001.pdf]

Angewandte Chemie
Eine Zeitschrift der Gesellschaft Deutscher Chemiker

Supporting Information

A Striking Mode of Activation of Carbon Disulfide with a Cooperative Bis(silylene)

*Marcel-Philip Luecke, Luisa Giarrana, Arseni Kostenko, Tobias Gensch, Shenglai Yao, and Matthias Driess**

anie_202110398_sm_miscellaneous_information.pdf

Table of Contents

1. **General Considerations** ... 3
2. **Syntheses and Characterization** ... 5
   2.1 **Compound S1** ... 5
   2.2 **Synthesis of compound S2** ... 5
   2.3 **Compound 3** ... 6
   2.4 **Compound 1*Mes*** ... 7
   2.5 **Compound 2*Ph* (*Ph*TpSi2CS2)** ... 7
   2.6 **Compound 2*Mes* (*Mes*TpSi2CS2)** ... 8
   2.7 **Compound 4** ... 10
   2.8 **Compound 6 (dithiasilolane)** ... 11
   2.9 **Compound 8 (thiadisiletane-3-thione)** ... 11
   2.10 **Compound 9 (trithia-silabicyclo[3.2.0]-dithione)** ... 12
   2.11 **Compound 10** ... 13
3. **NMR Spectra** ... 14
   3.1 **Compound S1** ... 14
   3.2 **Compound S2** ... 16
   3.3 **Compound 2*Ph*** ... 17
   3.4 **Compound 2*Mes*** ... 20
   3.5 **Compound 3** ... 24
   3.6 **Compound 4** ... 26
   3.7 **Compound 6** ... 27
   3.8 **Compound 8** ... 28
   3.9 **Compound 10** ... 30
4. **FT-IR Spectra** ... 32
   4.1 **Compound 1*Mes*** ... 32
   4.2 **Compound 2*Ph*** ... 33
   4.3 **Compound 2*Mes*** ... 33
   4.4 **Compound 3** ... 34
   4.5 **Compound 4** ... 34
   4.6 **Compound 6** ... 35
   4.7 **Compound 7** ... 35
   4.8 **Compound 8** ... 36

5. Calculations ... 36
5.1 Compound 2 – DFT ... 36
5.2 Compound 6 – NBO ... 71
6. Crystallographic Data ... 79
6.1 Compound S2 ... 79
6.2 Compound 2^{Mes} ... 80
6.3 Compound 3 ... 81
6.4 Compound 4 ... 82
6.5 Compound 6 ... 83
6.6 Compound 8 ... 84
6.7 Compound 9 ... 85
6.8 Compound 10 ... 86
7. References ... 87

1. General Considerations

All experiments and manipulations were carried out under dry oxygen-free nitrogen using standard Schlenk techniques or inside a MBraun glovebox. Solvents were dried by standard methods and freshly distilled prior to use. The NMR spectra were recorded on Bruker spectrometers Avance II 500 (^{1}H, 500.1 MHz; 13C{1H}, 125.8 MHz; ^{29}Si{1H}, 99.0 MHz), 400 (^{1}H, 400.1 MHz; 13C{1H}, 99.4 MHz; ^{29}Si{1H}, 79.5 MHz) and 200 (^{1}H, 200.1 MHz; 13C{1H}, 50.3 MHz) with residual solvent signals as internal reference (^{1}H NMR: benzene-d_6, 7.16 ppm, THF-d_8, 1.73 ppm, 3.58 ppm; $^{13}C\{^{1}H\}$ NMR: benzene-d_6 128.1 ppm, THF-d_8, 25.3 ppm, 67.2 ppm). High-resolution ESI (electrospray ionization) or APCI (atmospheric-pressure chemical ionization) mass spectra were measured on an Orbitrap LTQ XL by Thermo Scientific. Elemental analyses were recorded in a Thermo Flash EA 1112 Organic elemental analyzed and HEKAtech EuroEA 3000. The ATR/IR spectroscopic measurements were recorded on a Thermo Fisher Scientific Spectrometer inside a glovebox. Vibration modes are given in wavenumbers (cm^{-1}). Abbreviations: (vs) very strong, (s) strong, (m) middle, (w) weak and (br) broad. Commercially available reagents were purchased from Sigma-Aldrich, Acros, Alfa-Assar or abcr and used as received. The starting materials 1,4-bis(2-bromophenyl) benzene,[1] *N*,*N′*-di-tert-butyl(phenylamidinato)-chlorosilylene,[2] phenyl-substituted NHSi,[3], 2-(methylamino)pyridine-substituted silylene **5**,[4] 2-bromobiphenyl and 1,4-terphenyl bridged bis(silylene)[5] **1** were prepared according to reported literature procedures.

Molecule Index of new Compounds:

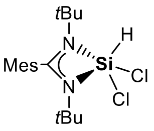

S1

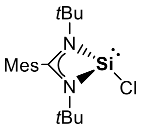

S2

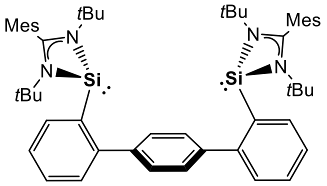

1$^{\mathbf{Mes}}$

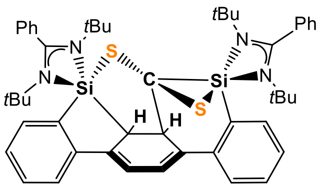

2$^{\mathbf{Ph}}$

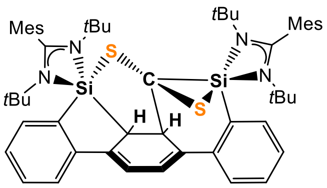

2$^{\mathbf{Mes}}$

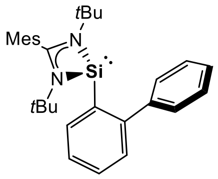

3

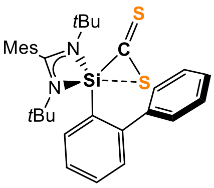

4

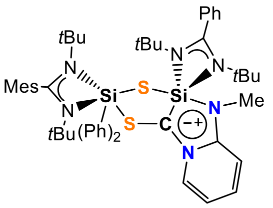

6

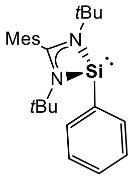

7

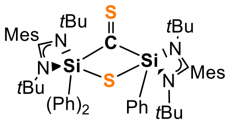

8

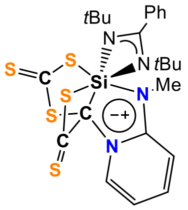

9

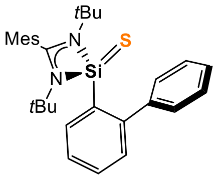

10

Single crystal X-ray structure analyses: Crystals were mounted on a glas capillary in perfluorinated oil and measured in a cold N_2 flow. The data for all compounds were collected on an Agilent Technologies SuperNova device (single source) at 150 K (Cu-K$_{\alpha}$ radiation, $\lambda = 1.54184$ Å). All structures were solved by direct methods and refined on F^2 with the SHELX-97 software.[6] All atoms except for hydrogen atoms were calculated and considered isotopically according to a riding model. Compound **6** crystallized with free yet severely disordered diethylether molecules in the asymmetric unit which were treated using the SQUEEZE routine in PLATON. The CCDC numbers CCDC 2116268 (**S2**), 2099865 (**2**$^{\mathbf{Mes}}$), 2116269 (**3**), 2099867 (**4**), 2099866 (**6**), 2099868 (**8**), 2099864 (**9**) and 2116270 (**10**) contain the supplementary crystallographic data for this paper. These data can be obtained free of charge by contacting The Cambridge Crystallographic Data Centre, 12, Union Road, Cambridge CB2 1EZ, UK; fax: +44 1223 336033.

2. Syntheses and Characterization

2.1 Compound S1

To a suspension of mesityl lithium (8.00 g, 63.5 mmol, 1.0 equiv.) in Et_2O (200 ml) *N,N'*-Di-*tert*-butylcarbodiimid (12.5 ml, 10.0 g, 64.8 mmol, 1.0 equiv.) was added at –78 °C. After warming up to room temperature, the reaction was stirred for 3 h. The suspension was added to a solution of trichlorosilane (9.80 ml, 13.2 g, 97.1 mmol, 1.5 equiv) in Et_2O (50 ml) at –78 °C. The mixture was warmed up to room temperature and stirred overnight. The solution was filtered and concentrated and then kept at at –30 °C overnight affording **S1** as colorless crystals suitable for X-ray diffraction analysis. After removal of Et_2O by filtration, compound **S1** was obtained in 51 % (12.0 g, 32.1 mmol) isolated yields. Compound **S1** is not air stable and generally good soluble in organic solvents except for ^{n}hexane.

^{1}H NMR (500.1 MHz, C_6D_6, 298 K): δ/ppm = 6.80 (s, 1H, Si*H*), 6.46 (d, $^4J_{HH}$ = 0.5 Hz, 2H, H_{Ar}), 2.07 (s, 6H, *o*-MesCH_3), 1.96 (s, 3H, *p*-MesCH_3), 1.16 (s, 18H, 2x NC(CH_3)$_3$). **^{13}C{^{1}H} NMR** (50.3 MHz, C_6D_6, 298 K): δ/ppm = 171.2 (s, C_{quart}, N*C*N), 140.3 (s, *p*-Mes*C*CH$_3$, C_{quart}), 135.8 (s, 2x *o*-Mes*C*CH$_3$, C_{quart}), 130.0 (s, C_{quart}, C_{Ar}), 128.7 (s, C_{Ar}), 128.6 (s, C_{Ar}), 55.8 (s, C_{quart}, 2x N*C*(CH$_3$)$_3$), 30.7 (s, 9x NC(*C*H$_3$)$_3$), 21.3 (s, *p*-MesC*C*H$_3$), 20.7 (s, 2x *o*-MesC*C*H$_3$). **^{1}H,^{29}Si{^{1}H} NMR** (99.0 MHz, C_6D_6, 298 K, optimized for J = 200 Hz): δ/ppm = 6.79/–96.7 (*Si*H).

2.2 Synthesis of compound S2

A mixture of **S1** (12 g, 32.1 mmol, 1.0 equiv.) and LiHMDS·Et_2O (7.80 g, 32.1 mmol, 1.0 equiv.) was dissolved in cold Et_2O (200 ml) and cooled to –78 °C. After warming up to room temperature, the mixture was stirred for 1 h turning from yellow to orange. After the solution was filtered and concentrated under reduced pressure, the solution was kept at –30 °C overnight affording **S2** as colorless crystals. Et_2O was removed by filtration and the title compound **S2** was obtained in 75 % (8.5 g, 32.4 mmol) isolated yields. Single crystals suitable for X-ray diffraction analysis of **S2** were obtained from a concentrated solution in Et_2O at –30 °C. Compound **S2** is not air stable and generally good soluble in organic solvents except for ^{n}hexane.

^{1}H NMR (500.1 MHz, C_6D_6, 298 K): δ/ppm = 6.52 (s, 1H, H_{Ar}), 6.49 (s, 1H, H_{Ar}), 2.26 (s, 3H, MesCH_3), 2.02 (s, 3H, MesCH_3), 1.97 (s, 3H, MesCH_3), 1.12 (s, 18H, NC(CH_3)). **^{13}C{^{1}H} NMR** (125.8 MHz, C_6D_6, 298 K): δ/ppm = 166.9 (s, C_{quart}, N*CN*), 139.8

(s, C_{quart}, *p*-MesCCH_3,), 136.8 (s, C_{quart}, *o*-MesCCH_3), 135.0 (s, C_{quart}, *o*-MesCCH_3,), 129.8 (s, C_{quart}, C_{Ar}), 128.6 (s, C_{Ar}), 128.5 (s, C_{Ar}), 54.1 (s, C_{quart}, 2x N$C(CH_3)_3$), 30.7 (s, 9x NC($CH_3)_3$), 21.1 (s, MesCH_3), 21.0 (s, m-MesCH_3), 20.6 (s, MesCH_3). **^{29}Si{^{1}H} NMR** (99.0 MHz, C_6D_6, 298 K): δ/ppm = 18.3 (s).

2.3 Compound 3

To a suspension of 2-bromobiphenyl (212 µl, 1.23 mmol, 1.0 equiv.) and Et_2O (5 ml) at –78 °C ^{n}Buli (615 µl, 2.0 M solution in THF, 1.23 mmol, 1.0 equiv.) was added via syringe. After stirring for 30 min at room temperature, all volatiles were removed under reduced pressure. The residue was re-dissolved in Et_2O (5 ml) and added to a solution of *N*,*N′*-di-tert-butyl(mesitylamidinato) chlorosilylene **S2** in Et_2O (10 ml) at –78 °C. After stirring for 1 hour at room temperature, the solution was filtered and Et_2O was slowly removed under reduced pressure without heating. The title compound **3** was obtained quantitatively (598 mg, 1.23 mmol) and was isolated as a crystalline orange solid. Single crystals suitable for X-ray diffraction analysis of **3** were obtained from a concentrated solution of **3** in Et_2O at –30 °C. Compound **3** is not stable in solution and slowly reacts to a silane via an intramolecular C-H activation reaction. Do not heat during work-up. **3** is stable in the solid state under an inert atmosphere for months.

^{1}H NMR (500.1 MHz, C_6D_6, 298 K): δ/ppm = 7.98 (dd, $^3J_{HH}$ = 7.3 Hz, $^4J_{HH}$ = 1.3 Hz, 1H, H_{Ar}), 7.82 (dd, $^3J_{HH}$ = 8.2 Hz, $^4J_{HH}$ = 1.3 Hz, 2H, H_{Ar}), 7.46 (dd, $^3J_{HH}$ = 7.6 Hz, $^4J_{HH}$ = 1.5 Hz, 1H, H_{Ar}), 7.40 – 7.37 (m, 3H, H_{Ar}), 7.31 (td, $^3J_{HH}$ = 7.3 Hz, $^4J_{HH}$ = 1.5 Hz, 1H, H_{Ar}), 7.25 – 7.21 (m, 3H, H_{Ar}, 1 H), 6.63 (s, 1H, *m*-Mes*H*), 6.53 (s, 1H, *m*-Mes*H*), 2.56 (s, 3H, *p*-MesCH_3), 2.06 (s, 3H, *p*-MesCH_3), 2.02 (s, 3H, *p*-MesCH_3), 1.04 (s, 18H, NC($CH_3)_3$). **^{13}C{^{1}H} NMR** (125.8 MHz, C_6D_6, 298 K): δ/ppm = 157.3 (s, C_{quart}, N*C*N), 152.1 (s, C_{quart}, C_{Ar}), 146.1 (s, C_{quart}, C_{Ar}), 144.7 (s, C_{quart}, C_{Ar}), 139.1 (s, C_{quart}, C_{Ar}), 137.6 (s, C_{quart}, C_{Ar})136.7 (s, C_{quart}, C_{Ar}), 132.8 (s, C_{Ar}), 131.4 (s, C_{Ar}), 130.2 (s, C_{Ar}), 128.8 (s, C_{Ar}), 128.7 (s, C_{Ar}), 128.6 (s, C_{Ar}), 126.7 (s, C_{Ar}), 125.8 (s, C_{Ar}), 53.4 (s, C_{quart}, N$C(CH_3)_3$), 30.9(s, NC($CH_3)_3$), 21.9 (s, MesCH_3), 21.0 (s, MesCH_3), 20.7 (s, MesCH_3). **^{29}Si{^{1}H} NMR** (79.5 MHz, C_6D_6, 298 K): δ/ppm = 17.1 (s). **^{1}H,^{29}Si HMQC NMR** (400.13/79.45 MHz, C_6D_6, 298 K, optimized for J = 7 Hz): δ/ppm = 7.98/17.1 (*Si*H_{Ar}), 7.46/17.1 (*Si*H_{Ar}), 7.37/17.1 (*Si*H_{Ar}), 1.04/17.1 (*Si*NC($CH_3)_3$).

2.4 Compound 1^{Mes}

A diethlyether suspension (10 ml) of 1,4-bis(2-bromophenyl) benzene[1] (400 mg, 1.03 mmol, 1.0 equiv.) was cooled –78 °C and ^{s}BuLi (1.53 ml, 1.3 M in cyclohexane, 2.00 mmol, 2.0 equiv.) was added via syringe. After warming up to room temperature, the solution turned from slight yellow to a suspension containing a white precipitate. After stirring for 4 h at room temperature, the suspension was cooled to –78 °C and a solution of *N*,*N′*-di-tert-butyl(mesitylamidinato)-chlorosilylene **S2** (674 mg, 2.00 mmol, 2.0 equiv.) in Et_2O (10 ml) was added via syringe. The cooling bath was removed and the suspension was stirred overnight to give a yellow suspension. All volatiles were removed under reduced pressure and the residue was washed with Et_2O (3x 2 ml). After removal of the solvent under reduced pressure, the residue was washed with THF (2x 3 ml) under removal of the residual metathesis salt (LiCl). The product was dried under reduced pressure and $\mathbf{1}^{Mes}$ was obtained as a yellow solid in 56 % (480 mg, 0.577 mmol) isolated yields. Single crystals suitable for X-ray diffraction analysis of $\mathbf{1}^{Mes}$ could not be obtained after several attempts. $\mathbf{1}^{Mes}$ is not air stable and not soluble in C_6D_6 and THF-d_8. NMR date was recorded in the solid state.

^{13}C{^{1}H} CP/MAS-NMR (10 kHz, 298 K): δ/ppm = 159.3 (s, C_{quart}, N*C*N), 150.5 (s, C_{Ar}), 146.2 (s, C_{Ar}), 141.6 (s, C_{Ar}), 139.2 (s, C_{Ar}), 137.6 (s, C_{Ar}), 136.3 (s, C_{Ar}), 133.5 (s, C_{Ar}), 131.5 (s, C_{Ar}), 129.9 (s, C_{Ar}), 127.9 (s, C_{Ar}), 125.4 (s, C_{Ar}), 53.7 (s, C_{quart}, 4x N*C*$(CH_3)_3$), 31.7 (s, 4x NC(CH_3)$_3$), 22.4 .3 (s, 2x *o*-MesCH_3), 20.1 (s, *p*-MesCH_3). **^{29}Si{^{1}H} CP/MAS NMR** (10 kHz, 298 K): δ/ppm = 16.5 (s, 2x *Si*).**FT-IR (ATR):** ṽ (cm^{-1}) = 3043 (w), 2296 (w), 2921 (w), 2868 (w), 1609 (w), 1581 (w), 1453 (w), 1430 (w), 1395 (vs), 1389 (vs), 1359 (s), 1296 (w), 1267 (w), 1224 (w), 1206 (s); **APCI-MS** m/z (%): Calcd. for $[C_{54}H_{70}N_4Si_2+H]^+$**:** 831.5212, found: 831.5208. **Melting Point:** T = 297°C.

2.5 Compound 2^{Ph} (*Ph*TpSi$_2$CS$_2$)

To a solution of 1,4-terphenyl bis(silylene) $\mathbf{1}^{Ph}$ (50 mg, 0.0669 mmol, 1.0 equiv.) in toluene (5 ml) at room temperature an excess of CS_2 was added via syringe. The pale yellow solution was stirred for 30 min at room temperature. After filtration the solution was concentrated under reduced pressure and kept at room temperature overnight to give compound $\mathbf{2}^{Ph}$ as yellow crystalline material in 63 % (35 mg, 0.0425 mmol) isolated yields after filtration. Single crystals suitable for X-ray diffraction analysis could not be obtained after several attempts. Compound $\mathbf{2}^{Ph}$ is not air stable and generally not good soluble in common organic solvents.

^{1}H NMR (500.1 MHz, THF-d_8, 298 K): δ/ppm = 7.98 (d, $^{3}J_{HH}$ = 7.3 Hz, 1H, H_{Ar}), 7.82 (d, $^{3}J_{HH}$ = 7.2 Hz, 1H, H_{Ar}), 7.79 – 7.76 (m, 1H, H_{Ar}), 7.69 (t, $^{3}J_{HH}$ = 8.7 Hz, 2H, H_{Ar}), 7.57 – 7.48 (m, 4H, H_{Ar}), 7.47 – 7.36 (m, 4H, H_{Ar}), 7.35 – 7.28 (m, 3H, H_{Ar}), 7.23 (td, $^{3}J_{HH}$ = 7.2 Hz, $^{4}J_{HH}$ = 1.5 Hz, 1H, H_{Ar}), 7.19 (t, $^{3}J_{HH}$ = 7.2 Hz, 1H, H_{Ar}), 6.60 (dd, $^{3}J_{HH}$ = 5.8 Hz, $^{4}J_{HH}$ = 3.3 Hz, 1H, C(sp^{2})*H*), 6.35 (d, $^{3}J_{HH}$ = 5.8 Hz, 1H, C(sp^{2})*H*), 3.11 (d, $^{3}J_{HH}$ = 11.0 Hz, 1H, SiC*H*), 2.23 (dd, $^{4}J_{HH}$ = 3.3 Hz, $^{3}J_{HH}$ = 11.0 Hz, 1H, (Si)(S)$_2$ CC*H*), 1.28 (d, $^{3}J_{HH}$ = 11.0 Hz, 18H, 2x NC(CH_3)$_3$), 1.00 (s, 9H, NC(CH_3)$_3$), 0.92 (s, 9H, NC(CH_3)$_3$). **^{13}C{^{1}H} NMR** (125.8 MHz, THF-d_8, 298 K): δ/ppm = 176.8 (s, C_{quart}, N*C*N), 171.0 (s, C_{quart}, N*C*N), 152.3 (s, C_{quart}), 150.9 (s, C_{quart}), 145.1 (s, C_{quart}), 143.5 (s, C_{quart}), 140.4 (s, C_{quart}), 136.5 (s, C_{quart}, C_{Ar}), 135.3 (s, C_{Ar}), 135.1 (s, C_{Ar}), 134.6 (s, C_{quart}), 134.1 (s, C_{quart}), 131.0 (s, C_{Ar}), 130.9 (s, C_{Ar}), 130.6 (s, C_{Ar}), 130.4 (s, C_{Ar}), 130.3 (s, C_{Ar}), 129.9 (s, C_{Ar}), 129.6 (s, C_{Ar}), 129.3 (s, C_{Ar}), 129.1 (s, C_{Ar}), 128.6 (s, C_{Ar}), 128.5 (s, C_{Ar}), 128.4 (s, C_{Ar}), 128.8 (s, C_{Ar}), 126.2 (s, C_{Ar}), 126.1 (s, C_{Ar}), 124.4 (s, C_{Ar}), 122.4 (s, *C(sp^{2})*H), 115.7 (s, *C(sp^{2})*H), 55.1 (s, C_{quart}, N*C*(CH$_3$)$_3$), 55.1 (s, C_{quart}, N*C*(CH$_3$)$_3$), 55.0 (s, C_{quart}, N*C*(CH$_3$)$_3$), 54.6 (s, C_{quart}, N*C*(CH$_3$)$_3$), 50.6 (s, Si*C(sp^{3})*), 43.9 (s, C_{quart}S$_2$), 35.8 (s, C*C(sp^{3})*H), 33.3 (s, NC(*C*H$_3$)$_3$), 32.2 (s, NC(*C*H$_3$)$_3$), 31.8 (s, NC(*C*H$_3$)$_3$), 31.6 (s, NC(*C*H$_3$)$_3$). **^{29}Si{^{1}H} NMR** (99.0 MHz, THF-d_8, 298 K): δ/ppm = –35.7 (s), –114.4 (s). **^{1}H,^{29}Si HMQC** (500.1/79.5 MHz, THF-d_8, 298 K, optimized for *J* = 7 Hz): δ/ppm = 3.15/–114.4 (*Si*CC*H*), 7.38/–114.3 (*Si*CCH_{Ar}) 2.28/–35.7 (*Si*CC*H),* 8.02/–35.7 (*Si*CCH_{Ar}). **FT-IR (ATR):** $\tilde{\nu}$ (cm^{-1}) = 3046 (w), 2969 (w), 2927 (w), 1582 (w), 1536 (w), 1494 (w), 1481 (w), 1441 (w), 1379 (vs), 1362 (vs), 1254 (w), 1199 (s), 1155 (w), 1124 (w), 1081 (w), 1047 (w), 10020 (w). **ESI-MS:** m/z (%): Calcd. for $[C_{49}H_{58}N_4S_2Si_2+H]^+$: 823.3714, found: 823.3715. **Melting Point:** T > 370°C (Decomposition).

2.6 Compound 2^{Mes} (*Mes*TpSi$_2$CS$_2$)

To a solution of bis(silylene) **1^{Mes}** (50 mg, 0.0601 mmol, 1.0 equiv.) in toluene (5 ml) at room temperature an excess of CS$_2$ was added via syringe. The pale yellow solution was stirred for 30 min. The solution was filtered and concentrated under reduced pressure. The title compound crystallized overnight at room temperature. After removal of the solvent by filtration, **2^{Mes}** was obtained as yellow plates in 73 % (40 mg, 0.0441 mmol) isolated yields. Single crystals suitable for X-ray diffraction analysis of **2^{Mes}** were obtained from a concentrated solution of **2^{Mes}** in toluene at room

temperature. Compound **2^{Mes}** is not air stable and generally not good soluble in common organic solvents.

^{1}H NMR (500.1 MHz, C_6D_6, 298 K): δ/ppm = 8.29 – 8.27 (m, 1H, H_{Ar}), 8.23 – 8.21 (m, 1H, H_{Ar}), 7.81 – 7.80 (m, 1H, H_{Ar}), 7.73 – 7.72 (m, 1H, H_{Ar}), 7.45 – 7.38 (m, 2H, H_{Ar}), 7.35 – 7.29 (m, 2H, H_{Ar}), 6.97 (dd, $^3J_{HH}$ = 5.8 Hz, $^4J_{HH}$ = 3.3 Hz, 1H, C(sp^2)*H*), 6.87 (d, $^3J_{HH}$ = 5.8 Hz, 1H, C(sp^2)*H*), 6.61 (d, $^3J_{HH}$ = 7.7 Hz, 2H, H_{Ar}), 6.57 (s, 1H, H_{Ar}), 6.52 (s, 1H, H_{Ar}), 3.69 (d, $^3J_{HH}$ = 10.9 Hz, 1H, SiC*H*), 2.71 (dd, $^3J_{HH}$ = 11.2 Hz, $^4J_{HH}$ = 3.2 Hz, 1H, SiCC*H*), 2.52 (s, 3H, *o*-MesCH_3), 2.42 (s, 3H, *o*-MesCH_3), 2.37 (s, 3H, *o*-MesCH_3), 2.31 (s, 3H, *o*-MesCH_3), 2.01 (d, $^4J_{HH}$ = 7.0 Hz, 6H, *p*-MesCH_3), 1.44 (s, 9H, NC(CH_3)$_3$), 1.38 (s, 9H, NC(CH_3)$_3$), 1.11 (s, 9H, NC(CH_3)$_3$), 0.88 (s, 9H, NC(CH_3)$_3$). **^{13}C{^{1}H} NMR** (125.8 MHz, C_6D_6, 298 K): δ/ppm = 177.1 (s, C_{quart}, NCN), 169.7 (s, C_{quart}, NCN), 151.9 (s, C_{quart}), 150.7 (s, C_{quart}), 144.6 (s, C_{quart}), 143.0 (s, C_{quart}), 140.5 (s, C_{quart}), 139.6 (s, C_{quart}), 139.2 (s, C_{quart}), 136.5 (s, C_{quart}), 136.4 (s, C_{quart}), 136.3 (s, C_{quart}), 134.9 (s, C_{quart}, C_{Ar}), 134.8 (s, C_{Ar}), 134.7 (s, C_{quart}, C_{Ar}), 134.2 (s, C_{Ar}), 132.6 (s, C_{quart}, C_{Ar}), 130.6 (s, C_{quart}, C_{Ar}), 130.4 (s, C_{Ar}), 129.4 (s, C_{Ar}), 129.2 (s, C_{Ar}), 128.5 (s, C_{Ar}), 128.3 (s, C_{Ar}), 126.8 (s, C_{Ar}), 126.6 (s, C_{Ar}), 125.9 (s, C_{Ar}), 125.2 (*C(sp^2)*H), 122.7 (s, C_{Ar}), 115.9 (*C(sp^2)*H), 55.0 (s, C_{quart}, N*C*(CH$_3$)$_3$), 54.7 (s, C_{quart}, N*C*(CH$_3$)$_3$), 54.6 (s, C_{quart}, N*C*(CH$_3$)$_3$), 54.0 (s, C_{quart}, N*C*(CH$_3$)$_3$), 49.6 (s, Si*C*(sp^3)), 42.1 (s, $C_{quart}S_2$), 34.9 (s, Si*C*(sp^3)H), 31.9 (s, NC(*C*H$_3$)$_3$), 30.7 (s, NC(*C*H$_3$)$_3$), 30.6 (s, NC(*C*H$_3$)$_3$), 30.02 (s, NC(*C*H$_3$)$_3$), 23.3 (s, Mes*C*H$_3$), 21.9 (s, Mes*C*H$_3$), 21.3 (s, Mes*C*H$_3$), 21.3 (s, Mes*C*H$_3$), 21.1 (s, Mes*C*H$_3$), 20.8 (s, Mes*C*H$_3$). **^{29}Si{^{1}H} NMR** (99.0 MHz, C_6D_6, 298 K): δ/ppm = –36.9 (s), –112.2. (s). **^{1}H,^{29}Si HMQC** (500.1/79.5 MHz, C_6D_6, 298 K, optimized for *J* = 7 Hz) δ/ppm = 2.70/–36.9 (*Si*CC*H*), 8.28/–36.9 (*Si*CCH_{Ar}), 3.68/ –112.2 (*Si*CC*H*), 8.23/–112.2 (*Si*CCH_{Ar}). **FT-IR (ATR):** ṽ (cm^{-1}) = 3048 (w), 2995 (w), 2975 (w), 2870 (w), 1611 (w), 1583 (w), 1523 (w), 1441 (w), 1431 (w), 1389 (vs),1360 (s), 1307 (w), 1254 (s), 1228 (s), 1217 (w), 1198 (vs) 1155 (w), 1126 (w), 1068 (s), 1047 (w), 1028 (w), 1021 (w). **Elemental Analysis (%):** Calcd. for $C_{55}H_{70}N_4S_2Si_2$: 6.17 (N), 72.80 (C), 7.78 (H), 7.07 (S) found: 6.24 (N), 71.91 (C), 7.67 (H), 7.12 (S). The reduced C content stems from SiC formation. **ESI-MS**: m/z (%): Calcd.for [$C_{55}H_{70}N_4Si_2$+H]$^+$: 907.4653, found: 907.4637. **Melting Point**: T > 270 °C (Decomposition).

Table S1. Comparison of the chemical shifts of **2*Ph*** and **2*Mes***.

Atom	Chemical Shift [ppm]			
	δ(**2*Ph***)	*J* [Hz]	δ(**2*Mes***)	*J* [Hz]
Si1	–114.3	-	–112.2	-
Si2	–35.7	-	–36.9	-
C1	43.9	-	42.1	-
H^A	3.11 (d)	$^3J_{HH}$ = 11.0	3.69 (d)	$^3J_{HH}$ = 10.9
H^B	2.23 (dd)	$^3J_{HH}$ = 11.0 $^4J_{HH}$ = 3.3	2.71 (dd)	$^3J_{HH}$ = 11.2, $^4J_{HH}$ = 3.2
H^C	6.60 (dd)	$^3J_{HH}$ = 5.8, $^4J_{HH}$ = 3.3	6.97 (dd)	$^3J_{HH}$ = 5.8, $^4J_{HH}$ = 3.3
H^D	6.35 (d)	$^3J_{HH}$ = 5.8	6.87 (d)	$^3J_{HH}$ = 5.8

2.7 Compound 4

A suspension of biphenyl-substituted silylene **3** (260 mg, 0.53 mmol, 1.0 equiv.) in Et_2O (4 ml) was cooled to –78 °C and an excess of CS_2 was added dropwise via syringe. The supernatant violet solution was filtered after 30 min at –78 °C. The residual pink solid **4** was dried under reduced pressure and isolated in yields of 77 % (230 mg, 0.41 mmol). Single crystals suitable for X-ray diffraction analysis of **4** were obtained from a concentrated solution of **4** in toluene at –30 °C overnight. Compound **4** is not air stable and generally good soluble in organic solvents except for ^{n}hexane.

^{1}H NMR (500.1 MHz, C_6D_6, 298 K): δ/ppm = 8.26 (d, $^3J_{HH}$ = 7.3 Hz, 1H, H_{Ar}), 7.52 (d, $^3J_{HH}$ = 7.3 Hz, 2H, H_{Ar}), 7.33 – 7.22 (m, 5H, H_{Ar}), 7.18 – 7.16 (m, 5H, H_{Ar}), 6.50 (s, 1H, *m*-Mes*H*), 6.46 (s, 1 H, *m*-Mes*H*), 2.22 (s, 3H, *p*-MesCH_3), 2.14 (s, 3H, *o*-MesCH_3), 1.95 (s, 3H, *o*-MesCH_3), 1.15 (s, 18H, 2x NC(CH_3)$_3$). **^{13}C{^{1}H} NMR** (125.8 MHz, C_6D_6, 298 K): δ/ppm = 274.8 (s, C_{quart}, Si*C*S_2), 182.1 (s, C_{quart}, N*C*N), 150.9 (s, C_{quart}, C_{Ar}), 143.0 (s, C_{quart}, C_{Ar}), 140.8 (s, C_{quart}, C_{Ar}), 136.3 (s, C_{quart}, C_{Ar}), 135.5 (s, C_{Ar}), 134.4 (s, C_{quart}, C_{Ar}), 132.4 (s, C_{quart}, C_{Ar}), 131.1 (s, C_{Ar}), 130.1 (s, C_{Ar}), 129.5 (s, C_{Ar}), 128.6 (s, C_{Ar}), 128.5 (s, C_{Ar}), 128.3 (s, C_{Ar}), 126.4 (s, C_{Ar}), 56.5 (s, C_{quart}, 2x N*C*(CH_3)$_3$), 30.3 (s, 2x NC(*C*H_3)$_3$), 22.2 (s, *p*-MesCH$_3$), 21.8 (s, *o*-Mes*C*H$_3$), 20.9 (s, *o*-Mes*C*H$_3$). **^{1}H,^{29}Si HMQC NMR** (400.1/79.5 MHz, C_6D_6, 298 K, optimized for *J* = 5 Hz): δ/ppm = 7.25/–90.9 (*SiH*$_{Ar}$). **FT-IR (ATR):** $\tilde{v}$ (cm^{-1}): 3051 – 2870 (w), 1611 (w), 1538 (vw), 1555 (vw), 1505 (w), 1464 – 1444 (w), 1424 (w), 1380 -1362 (s), 1301 (vw), 1275 (w), 1234 (w), 1193 (s), 1156 (w), 1122 (w), 1076 (s), 1032 (w), 1007 (vw),

997 (w), 948 (vw), 904 – 884 (w), 853 (s), 814 (vw), 789 (w), 776 (vs), 748 (s). **Elemental Analysis (%)**: Calcd. for $C_{31}H_{38}N_2S_2Si$ +$C_{3.5}H_4$: 4.98 (N), 70.41 (C), 8.24 (H), 11.39 (S), found: 5.18 (N), 69.70 (C), 8.61 (H), 10.92 (S). The reduced C content stems from SiC formation. ESI- and APCI-HR MS analyses show merely molecular fragments. **Melting Point:** T = 119°C.

2.8 Compound 6 (dithiasilolane)

To a suspension of thiasilirane **4** (50.0 mg, 0.089 mmol, 1.0 equiv.) in Et_2O (5 ml) 2-(methylamino)pyridine-substituted silylene[4] **5** (32.6 mg, 0.089 mmol, 1.0 equiv.) was added at room temperature and stirred for 30 min. The orange solution was filtered and afterwards concentrated under reduced pressure. Compound **6** was obtained as orange crystal rods from the concentrated solution in Et_2O overnight at –30 °C in 41 % (32.4 mg, 0.036 mmol, 1.0 equiv.) isolated yields. Single crystals suitable for X-ray diffraction analysis of **6** were obtained from a concentrated solution of **6** in Et_2O at –30 °C. Compound **6** is not stable in solution and shows poor solubility in polar solvents such as Et_2O and THF. Due to the observation of additional resonance signals in the ^{1}H and ^{13}C NMR spectra a full assignment was not possible.

^{29}Si{^{1}H} NMR (79.5 MHz, Tol-d_8, 223 K) δ/ppm = –71.3 (s, *Si*1), –73.5 (s, *Si*1). **^{1}H,^{29}Si HMQC NMR** (500.1/79.5 MHz, Tol-d_8, 223 K, optimized for J = 7 Hz): δ/ppm = 7.43/–71.3 (SiCCH_{Ar}), 3.15/–73.5 (*Si*NCH_3). **FT-IR (ATR):** $\tilde{v}$ (cm^{-1}): 3079 – 2864 (w), 1605 – 591 (w), 1520 (w), 1509 (w), 1489 – 1478 (w), 1458 (w), 1442 (w), 1392-1380 (s), 1361 (s), 1317 (vw), 1288 (s), 1247 (s), 1220 (vw), 1196 (s), 1178 (w), 1150 – 1116 (vw), 1085 (w), 1057 – 1050 (w), 1032 (vw), 2021 (w), 1007 (w), 925 (vw), 909 – 904 (vw), 881 (vw), 867-848 (w), 828 (w), 791- 783 (w), 775 – 738 (s), 703 (vs). **Elemental Analysis (%):** Calcd. for $C_{52}H_{68}N_6S_2Si_2$ + $C_4H_{10}O$: 8.65 (N), 69.23 (C), 8.09 (H), 6.60 (S), found: 8.88 (N), 69.32 (C), 8.18 (H), 6.44 (S). **ESI-MS:** (m/z): Calcd. for [$C_{52}H_{68}N_6S_2Si_2$] = 897.4558, found: 897.4552. **Melting Point:** T = 109°C.

2.9 Compound 8 (thiadisiletane-3-thione)

A suspension of thiasilirane **4** (30.0 mg, 0.053 mmol, 1.0 equiv.) in Et_2O (4 ml) was cooled to –78°C and a solution of phenyl-substitued silylene **7** (21.4 mg, 0.057 mmol, 1.0 equiv.) in Et_2O (1 ml) was added dropwise. The dark green mixture was warmed up to room temperature and stirred for 15 min. The resulting light green solution was filtered and concentrated under reduced pressure. Single crystals suitable for X-ray

diffraction analysis of **8** were obtained from a concentrated solution of **8** in C_6D_6 at room temperature. The obtained light green crystal rods were washed with cold Et_2O (2x 1 ml) and dried under reduced pressure to obtain compound **8** in 26% (12.8 mg, 0.0136 mmol) isolated yields. Compound **8** is air stable for 1 h and shows good solubility in common organic solvents.

^{1}H NMR (500.1 MHz, C_6D_6, 298 K): δ/ppm = 8.76 (d, $^3J_{HH}$ = 6.5 Hz, 2H, H_{Ar}), 8.03 (d, $^3J_{HH}$ = 7.2 Hz, 2H, H_{Ar}), 7.95 (d, $^3J_{HH}$ = 7.5 Hz, 1H, H_{Ar}), 7.31 (t, $^3J_{HH}$ = 7.7 Hz, 3H, H_{Ar}), 7.23 – 7.17 (m, 4H, H_{Ar}), 7.12 (d, $^3J_{HH}$ = 7.5 Hz, 1H, H_{Ar}), 6.96 (t, $^3J_{HH}$ = 7.5 Hz, 1H, H_{Ar}), 6.59 (s, 1H, H_{Ar}), 6.54 – 6.49 (m, 4H, H_{Ar}), 2.90 (s, 3H, *o*-MesCH_3), 2.83 (s, 3H, *o*-MesCH_3), 2.23 (s, 3H, *o*-MesCH_3), 2.14 (s, 3H, *o*-MesCH_3), 2.01 (s, 3H, *p*-MesCH_3), 1.99 (s, 3H, *p*-MesCH_3), 1.59 (s, NC(CH_3)$_3$), 1.32 (s, 9H, NC(CH_3)$_3$), 1.23 (s, 9H, NC(CH_3)$_3$), 0.92 (s, 9H, NC(CH_3)$_3$). **^{13}C{^{1}H} NMR** (125.8 MHz, C_6D_6, 298 K): δ/ppm = 267.3 (s, C_{quart}, *SiC(*S)Si), 172.4 (s, C_{quart}, N*C*N), 170.9 (s, C_{quart}, N*C*N), 149.5 (s, C_{quart}, C_{Ar}), 146.3 (s, C_{quart}, C_{Ar}), 145.3 (s, C_{quart}, C_{Ar}), 142.1 (s, C_{quart}, C_{Ar}), 139.9 (s, C_{quart}, C_{Ar}), 139.2 (s, C_{quart}, C_{Ar}), 138.9 (s, C_{quart}, C_{Ar}), 138.7 (s, 2x C_{Ar}), 137.6 (s, C_{quart}, C_{Ar}), 137.3 (s, C_{Ar}), 136.6 (s, C_{quart}, C_{Ar}), 135.8 (s, C_{Ar}), 133.5 (s, C_{quart}, C_{Ar}), 132.2 (s, C_{quart}, C_{Ar}), 131.7 (s, C_{Ar}), 131.4 (s, C_{Ar}), 129.3 (s, C_{Ar}), 128.9 (s, C_{Ar}), 128.7, (s, C_{Ar}), 128.4 (s, C_{quart}, C_{Ar}), 128.2 (s, C_{Ar}), 128.1 (s, C_{Ar}), 128.0 (s, C_{Ar}), 127.7 (s, C_{Ar}), 127.1 (s, C_{Ar}), 127.0 (s, C_{Ar}), 126.7 (s, C_{Ar}), 124.7 (s, C_{Ar}), 57.0 (s, C_{quart}, N*C*(CH_3)$_3$), 56.1 (s, C_{quart}, N*C*(CH_3)$_3$), 55.7 (s, C_{quart}, N*C*(CH_3)$_3$), 55.1 (s, C_{quart}, N*C*(CH_3)$_3$), 32.2 (s, NC(CH_3)$_3$), 31.3 (s, NC(CH_3)$_3$), 29.6 (s, NC(CH_3)$_3$), 29.2 (s, NC(CH_3)$_3$), 22.4 (s, 2x *o*-MesCH_3), 21.5 (s, 2x *o*-MesCH_3), 20.0 (s, 2x *p*-MesCH_3). **^{29}Si{^{1}H} NMR** (79.5 MHz, C_6D_6, 298 K): δ/ppm = –59.7 (s, *Si*2), –67.4 (s, *Si*1). **^{1}H,^{29}Si HMQC NMR** (400.1/79.5 MHz, C_6D_6, 298 K, optimized for J = 7 Hz): δ/ppm = 8.76/–59.7 (*Si*CCH_{Ar}), 7.95/–67.4 (*Si*CCH_{Ar}), 7.12/–67.4(*Si*CCH_{Ar}). **FT-IR (ATR):** $\tilde{\nu}$ (cm^{-1}) : 3038 – 2867 (w), 1658 (vw), 1612 (vw), 1550 – 1524 (w), 1476-1425 (w) 1391 (w), 1362 (w), 1306 (w), 1230 – 1082 (vs), 1060 (w), 983 (s), 949 (vw), 918 (vw). **ESI-MS:** (m/z): Calcd. for [$C_{55}H_{72}N_4S_2Si_2$] = 909.4810, found: 909.4810. **Melting Point:** T = 135°C.

2.10 Compound 9 (trithia-silabicyclo[3.2.0]-dithione)

To a solution of dithiasilolane **6** (40.0 mg, 0.045 mmol, 1.0 equiv.) in toluene (2 ml) an excess of CS_2 was added dropwise at room temperature. After stirring for 30 min at room temperature the pink solution was filtered and concentrated under reduced

pressure. Single crystals suitable for X-ray diffraction analysis of **9** were obtained from a concentrated solution of **9** in toluene at –30 °C after one week. The isolated crystals of compound **9** were found to be completely insoluble in common organic solvents (benzene, toluene). Compound **9** could only be isolated in very small quantities.

^{1}H,^{29}Si HMQC-NMR (500.1/79.5 MHz, Tol-d_8, 298 K, optimized for J = 7 Hz): δ/ppm = 2.91/–143.4 (SiNCH_3). **Elemental Analysis**: Calcd. for $C_{24}H_{30}N_4S_5Si + C_7H_8$: 9.36 (N), 56.84 (C), 5.85 (H), 24.47 (S), found: 9.80 (N), 56.78 (C), 5.64 (H), 24.23 (S).

In situ NMR spectroscopic analysis of the reaction mixture indicates the stoichiometric formation of the corresponding silathione **10**.

^{1}H NMR (500.1 MHz, C_6D_6, 298 K): δ/ppm = 8.37 (d, $^{3}J_{HH}$ = 6.9 Hz, 1H, H_{Ar}), 7.67 – 7.60 (m, 2H, H_{Ar}), 7.31 (t, $^{3}J_{HH}$ = 7.6 Hz, 2H, H_{Ar}), 7.24 (d, $^{4}J_{HH}$ = 1.3 Hz, 4H, H_{Ar}), 6.53 (d, $^{3}J_{HH}$ = 12.6 Hz, 2H, H_{Ar}), 2.32 (s, 3H, *o*-MesCH_3), 2.18 (d, $^{4}J_{HH}$ = 4.5 Hz 3H, *o*-MesCH_3), 1.99 (s, 3H, s*p*-MesCH_3), 1.10 (s, 18H, 2x NC(CH_3)$_3$). **^{1}H,^{29}Si HMQC NMR** (500.1/79.5 MHz, Tol-d_8, 298 K, optimized for J = 7 Hz): δ/ppm = 8.37/4.4 (SiCCH_{Ar}). **^{29}Si{^{1}H} NMR** (79.5 MHz, Tol-d_8, 298 K) δ/ppm = 4.9.

2.11 Compound 10

In a Y. Young NMR tube **4** (10.0 mg, 0,019 mmol, 1.0 equiv.) was dissolved in C_6D_6 (0.5 ml) and heated to 50 °C for 4 h. The color of the solution changed with increasing conversion from red to dark brown. After removal of the solvent under reduced pressure, the residue was re-dissolved in Et_2O (1.0 ml). The solution was filtered and transferred to a 5 ml Schlenk tube. The solvent was reduced to a volume of 0.2 ml upon which a dark red solution was obtained. Single crystals suitable for X-ray diffraction analysis of **10** were obtained after one week at –30 °C as colorless blocks. The conversion of thiasilirane **4** to silathione **10** is quantitative as observed by ^{1}H NMR spectroscopy showing the formation of **10** as sole product.

^{1}H NMR (500.1 MHz, C_6D_6, 298 K): δ/ppm = 8.39 (dd, $^{3}J_{HH}$ = 7.3 Hz, $^{4}J_{HH}$ = 2.5 Hz, 1H, H_{Ar}), 7.73 (dt, $^{3}J_{HH}$ = 8.0 Hz, $^{4}J_{HH}$ = 1.6 Hz, 2H, H_{Ar}), 7.36 (t, $^{3}J_{HH}$ = 7.6 Hz, 2H, H_{Ar}), 7.30 – 7.20 (m, 4H, H_{Ar}), 6.52 (s, 1H, *m*-Mes$H$), 6.48 (s, 1H, *m*-Mes$H$), 2.33(s, 3H, *p*-MesCH_3), 2.17 (s, 3H, *p*-MesCH_3), 1.97 (s, 3H, *p*-MesCH_3), 1.11 (s, 18H, NC(CH_3)$_3$). **^{13}C{^{1}H} NMR** (125.6 MHz, C_6D_6, 298 K): δ/ppm = 176.0 (s, C_{quart}, NCN),

152.0 (s, C_{quart}, C_{Ar}), 143.4 (s, C_{quart}, C_{Ar}), 140.3 (s, C_{quart}, C_{Ar}), 137.0 (s, C_{quart}, C_{Ar}), 135.3 (s, C_{Ar}), 135.0 (s, C_{quart}, C_{Ar}), 133.8 (s, C_{quart}, C_{Ar}), 132.6 (s, C_{Ar}), 131.1 (s, C_{Ar}), 130.4 (s, C_{Ar}), 129.4 (s, C_{Ar}), 128.5 (s, C_{Ar}), 127.6 (s, C_{Ar}), 127.5 (s, C_{Ar}), 126.2 (s, C_{Ar}), 55.5 (s, C_{quart}, N$C(CH_3)_3$), 30.5 (s, NC(CH_3)$_3$), 21.6 (s, MesCH_3), 21.5 (s, MesCH_3), 21.1 (s, MesCH_3). **^{1}H,^{29}Si HMQC NMR** (400.1/79.4 MHz, C_6D_6, 298 K, optimized for J = 4 Hz): δ/ppm = 7.29/ 5.9(SiH_{Ar}). **^{29}Si{^{1}H} NMR** (79.5 MHz, C_6D_6, 298 K): δ/ppm = 5.1 (s).

3. NMR Spectra

3.1 Compound S1

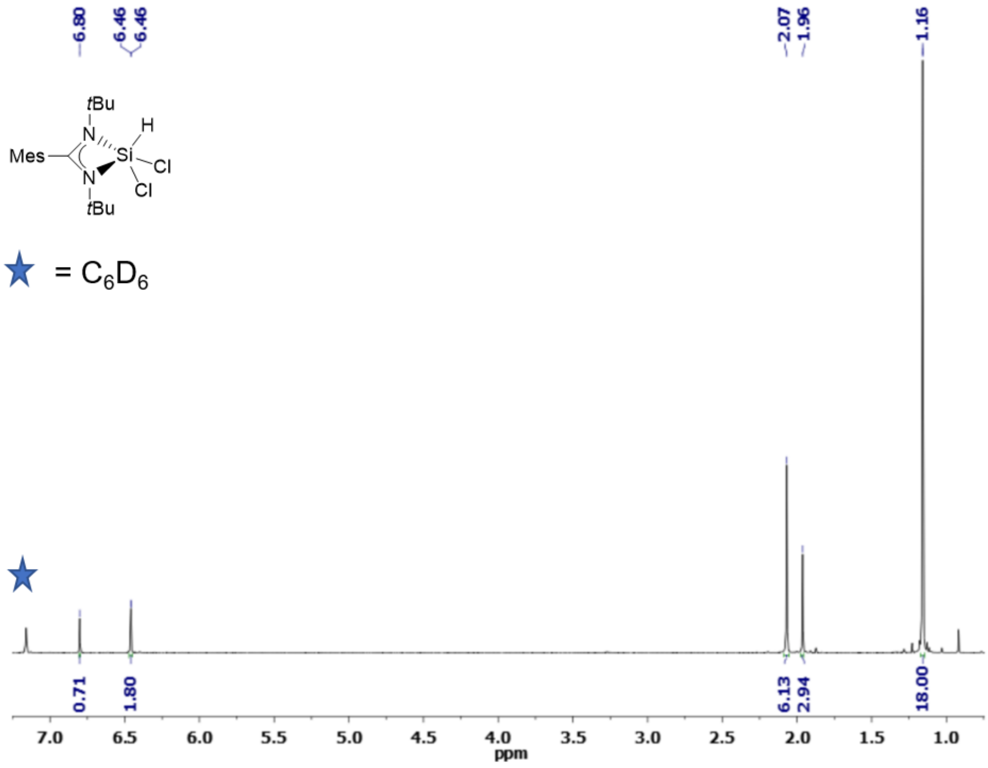

Figure S1. ^{1}H NMR spectrum of compound **S1** in C_6D_6 under 1 bar N_2 at 298 K.

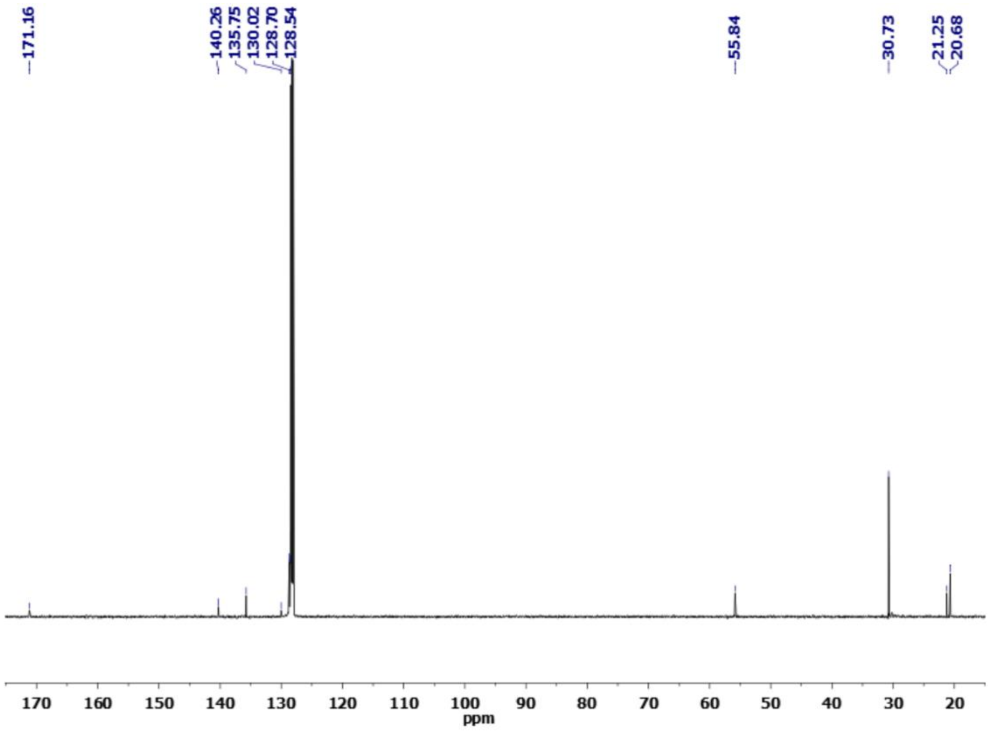

Figure S2. $^{13}C\{^{1}H\}$ NMR spectrum of compound **S1** in C_6D_6 under 1 bar N_2 at 298 K.

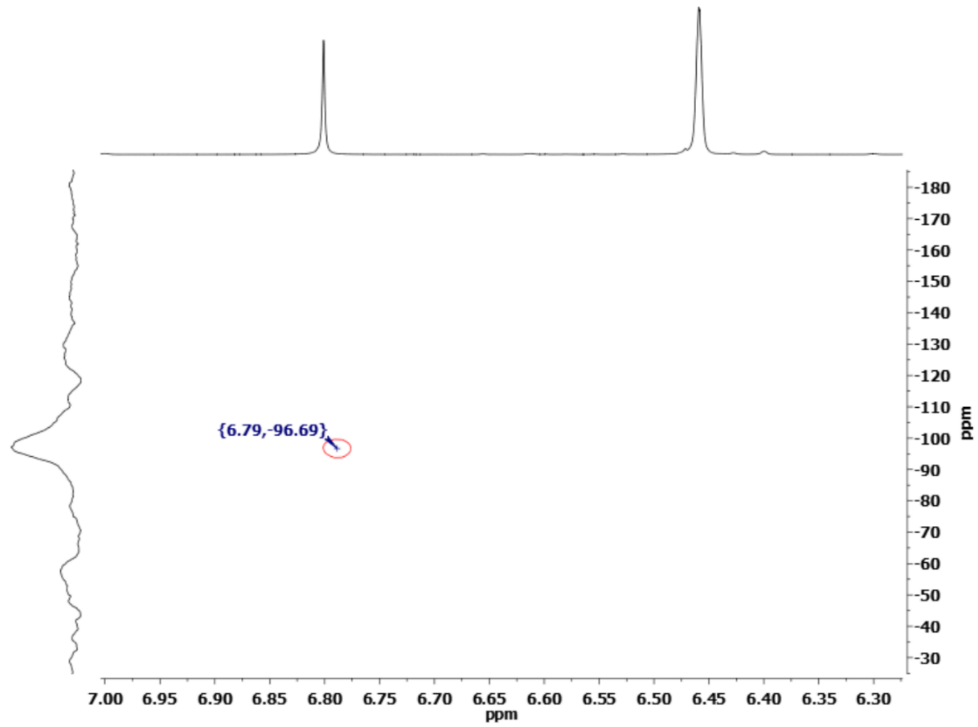

Figure S3. $^{1}H,^{29}Si$ HMQC NMR spectrum (optimized for $J_{Si,H}$ = 200 Hz) of compound **S1** in C_6D_6 under 1 bar N_2 at 298 K.

3.2 Compound S2

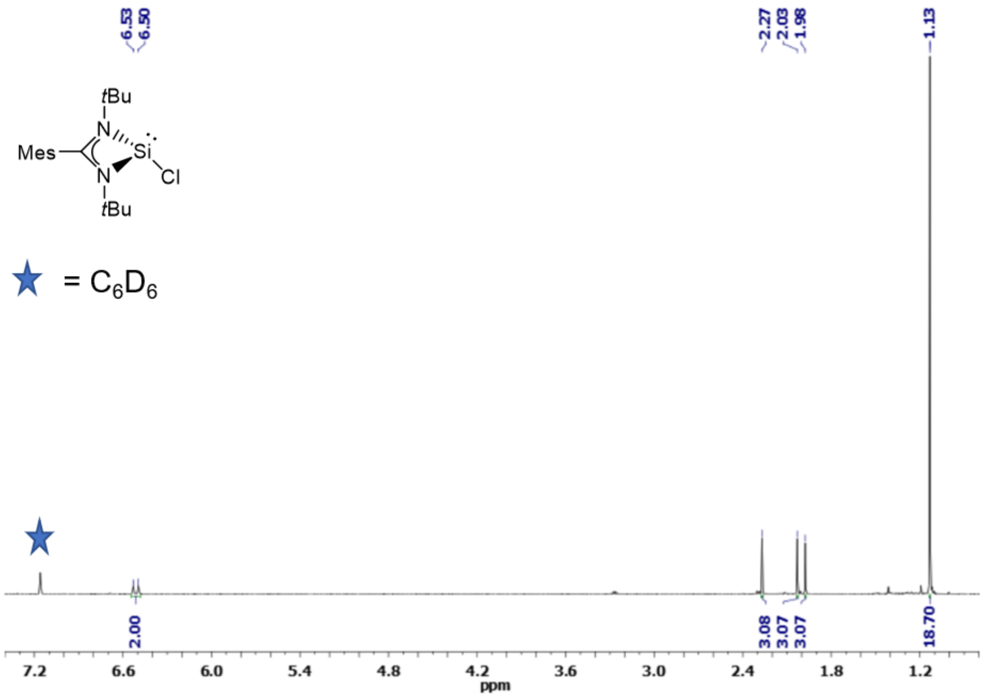

Figure S4. ^{1}H NMR spectrum of compound **S2** in C_6D_6 under 1 bar N_2 at 298 K.

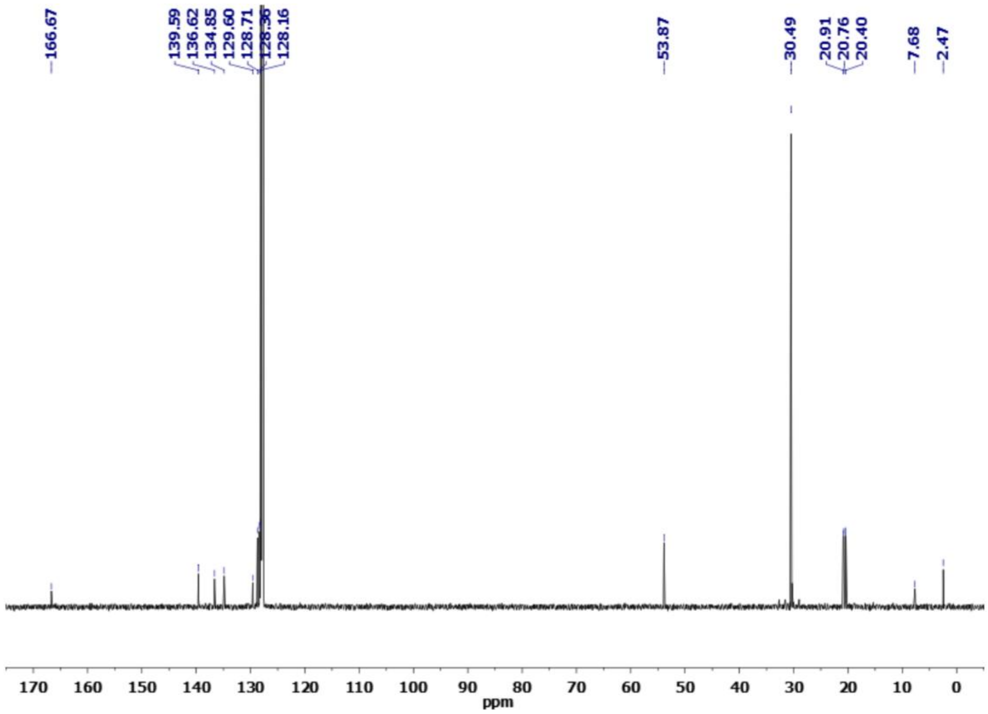

Figure S5. $^{13}C\{^{1}H\}$ NMR spectrum of compound **S2** in C_6D_6 under 1 bar N_2 at 298 K.

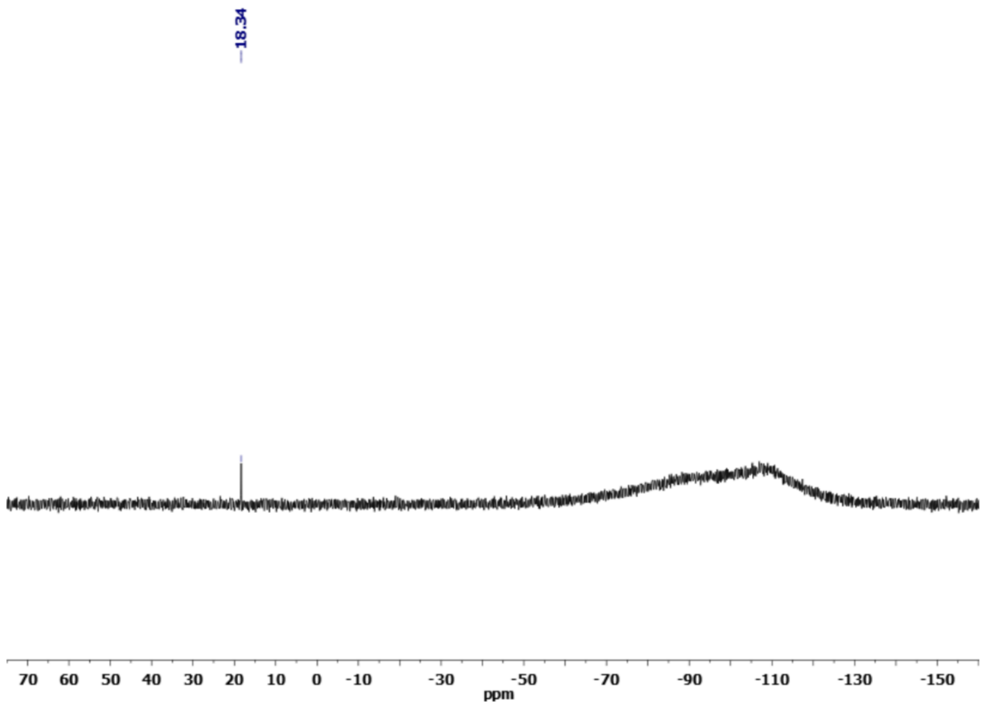

Figure S6. $^{29}Si\{^{1}H\}$ NMR spectrum of compound **S2** in C_6D_6 under 1 bar N_2 at 298 K.

3.3 Compound $\mathbf{2^{\mathit{Ph}}}$

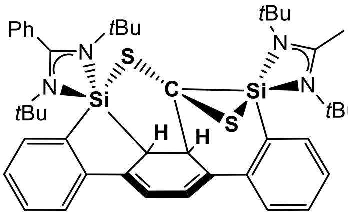

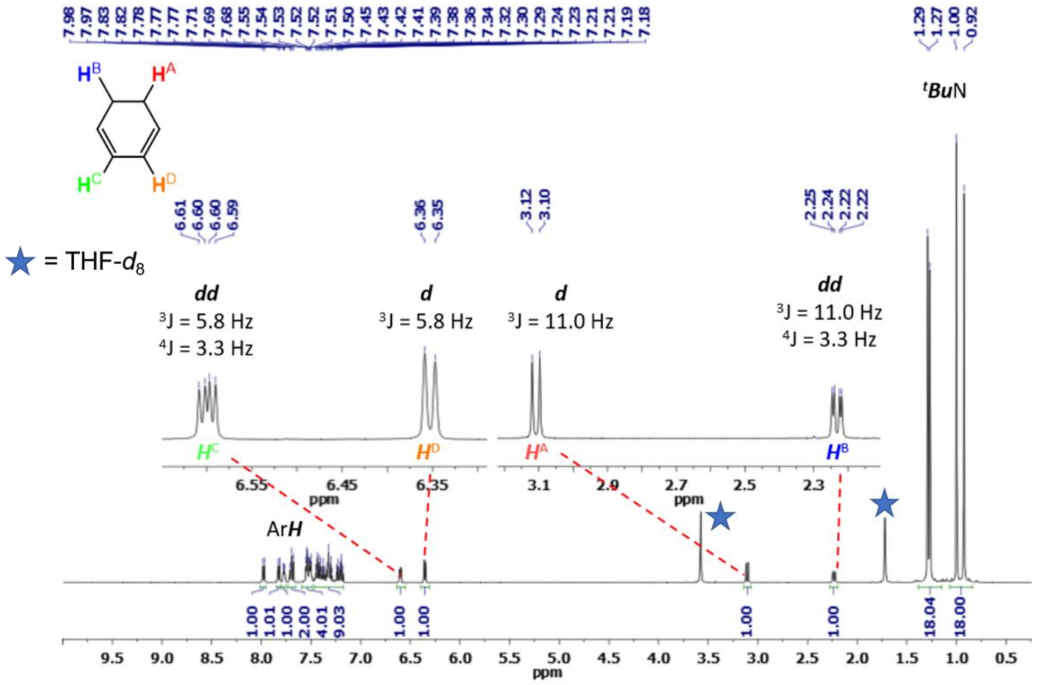

Figure S7. ^{1}H NMR spectrum of compound $\mathbf{2^{\mathit{Ph}}}$ in THF-d_8 under 1 bar N_2 at 298 K.

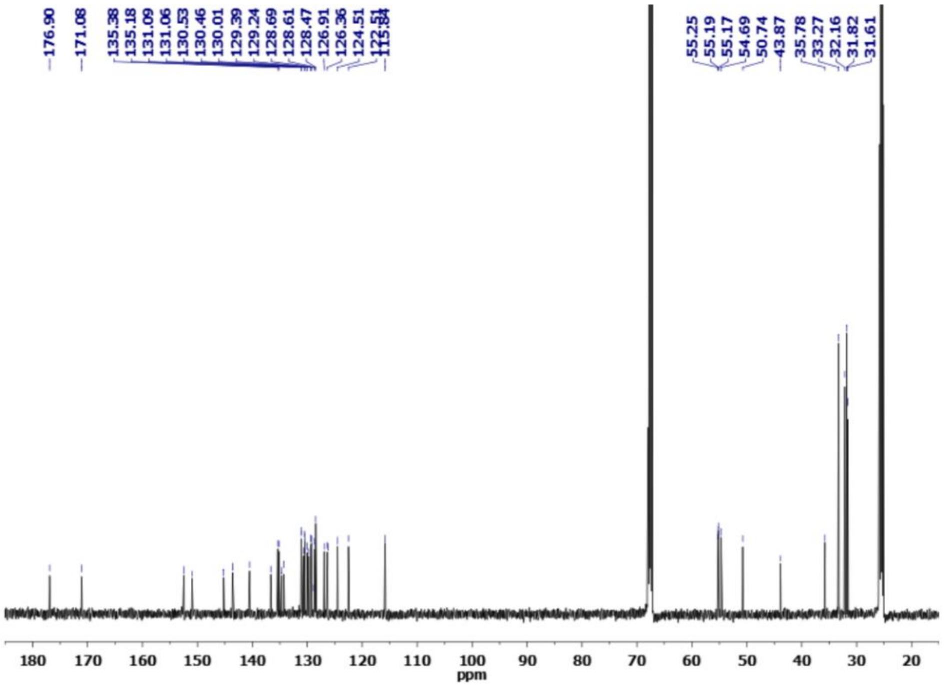

Figure 8. $^{13}C\{^{1}H\}$ NMR spectrum of compound **2*^Ph^*** in THF-d_8 under 1 bar N_2 at 298 K.

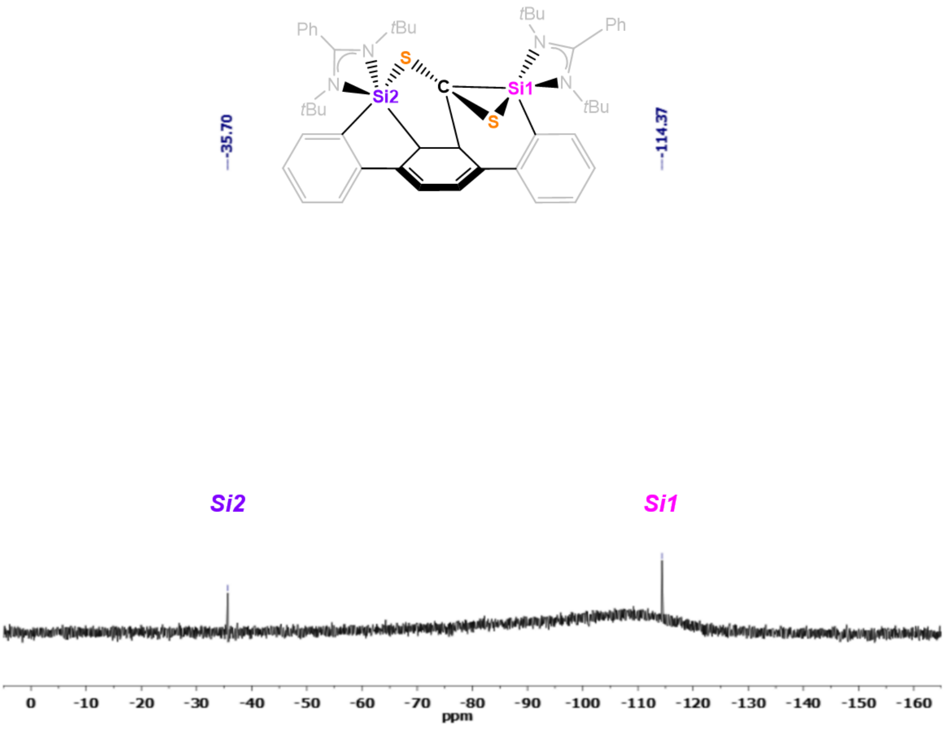

Figure S9. $^{29}Si\{^{1}H\}$ NMR spectrum of compound **2*^Ph^*** in THF-d_8 under 1 bar N_2 at 298 K.

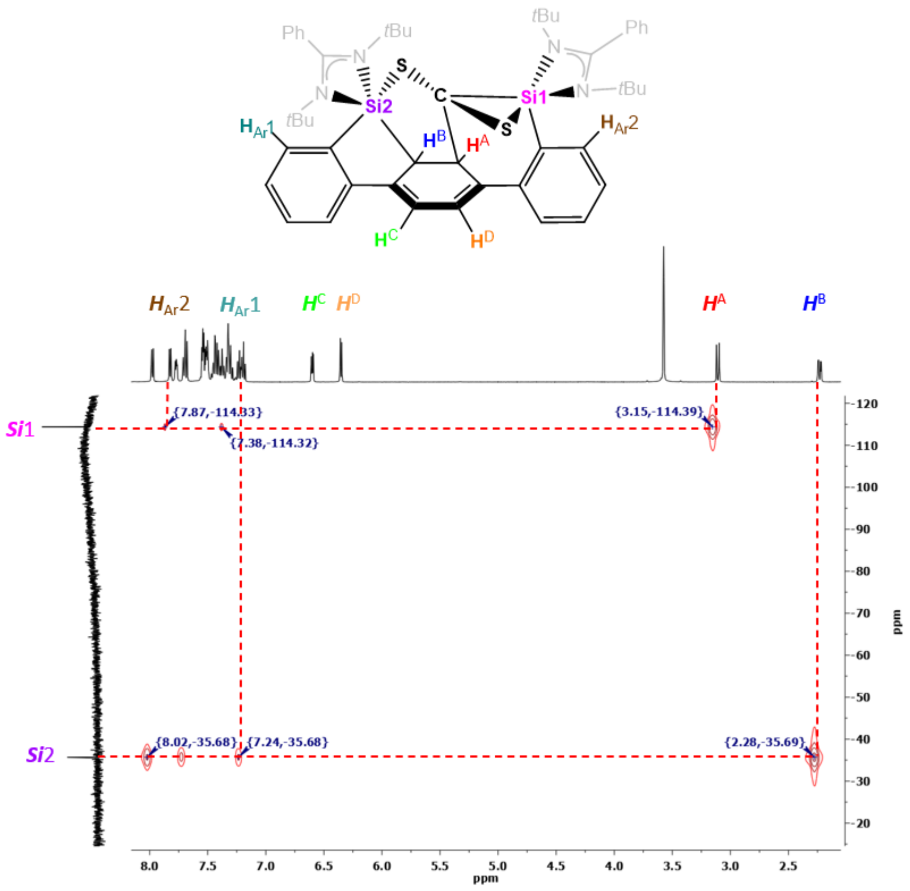

Figure S10. ^{1}H,^{29}Si HMQC NMR spectrum (optimized for $J_{Si,H}$ = 7 Hz) of compound $\mathbf{2}^{Ph}$ in THF-d_8 under 1 bar N_2 at 298 K.

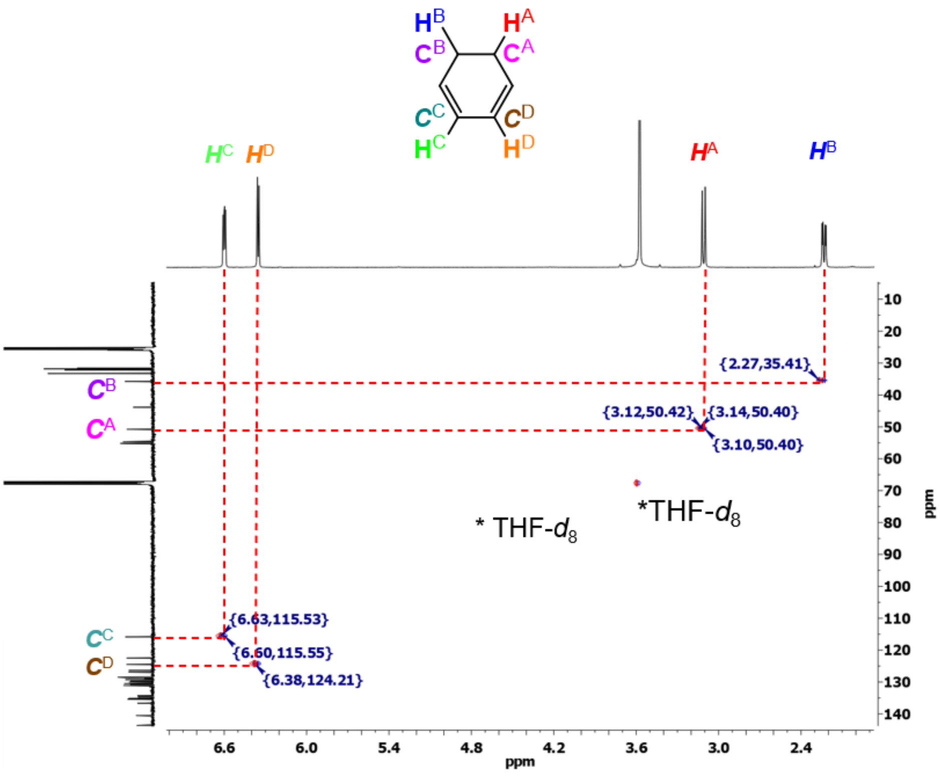

Figure S11. ^{1}H,^{13}C HSQC NMR spectrum of compound $\mathbf{2}^{Ph}$ in THF-d_8 under 1 bar N_2 at 298 K.

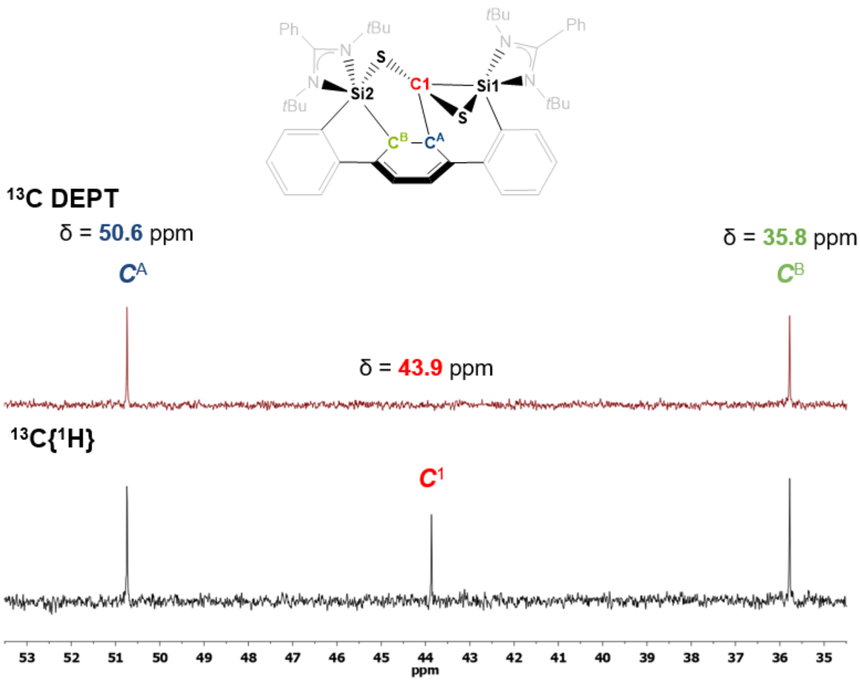

Figure S12. Section of ^{13}C{^{1}H} NMR spectrum (bottom) and ^{13}C 135 DEPT spectrum (top) of compound **2*Ph***.

3.4 Compound 2*Mes*

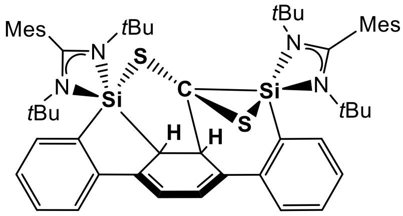

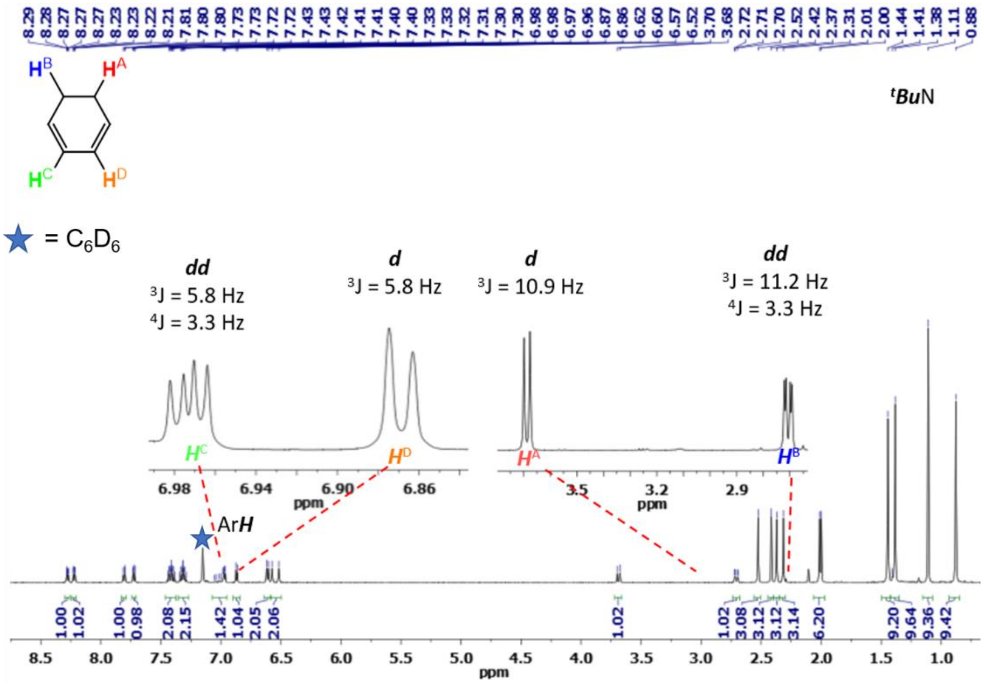

Figure S13. ^{1}H NMR spectrum of compound **2*Mes*** in C_6D_6 under 1 bar N_2 at 298 K.

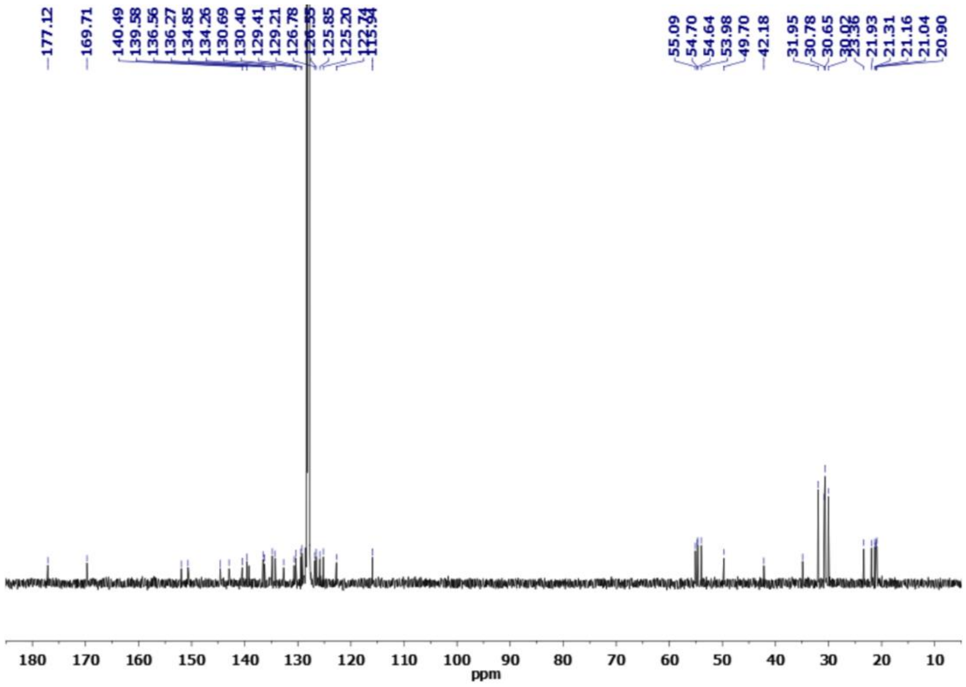

Figure S14. $^{13}C\{^1H\}$ NMR spectrum of compound **2^{Mes}** in C_6D_6 under 1 bar N_2 at 298 K.

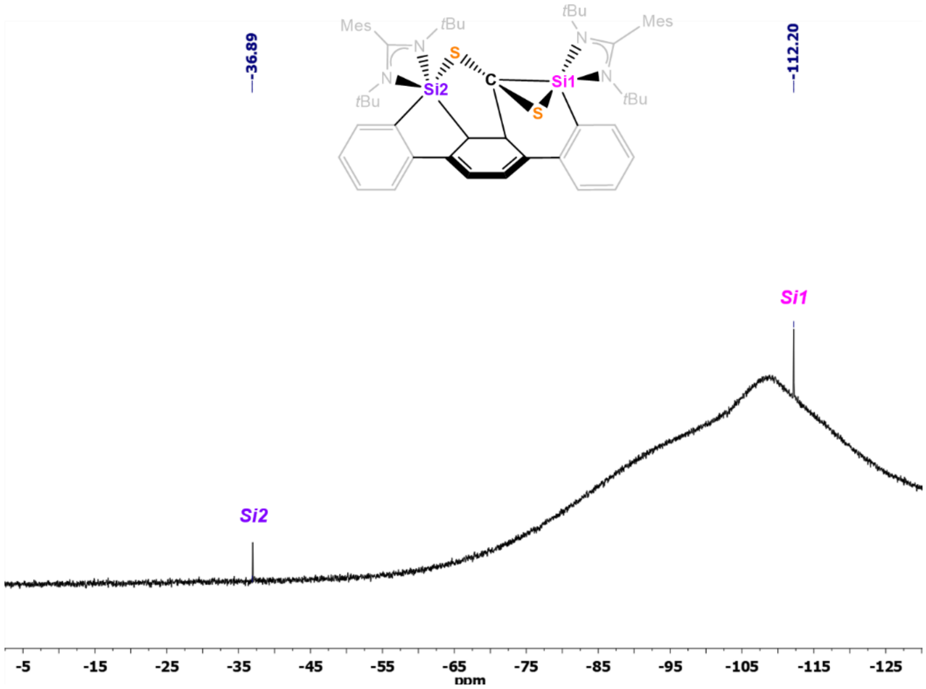

Figure S15. $^{29}Si\{^1H\}$ NMR spectrum of compound **2^{Mes}** in C_6D_6 under 1 bar N_2 at 298 K.

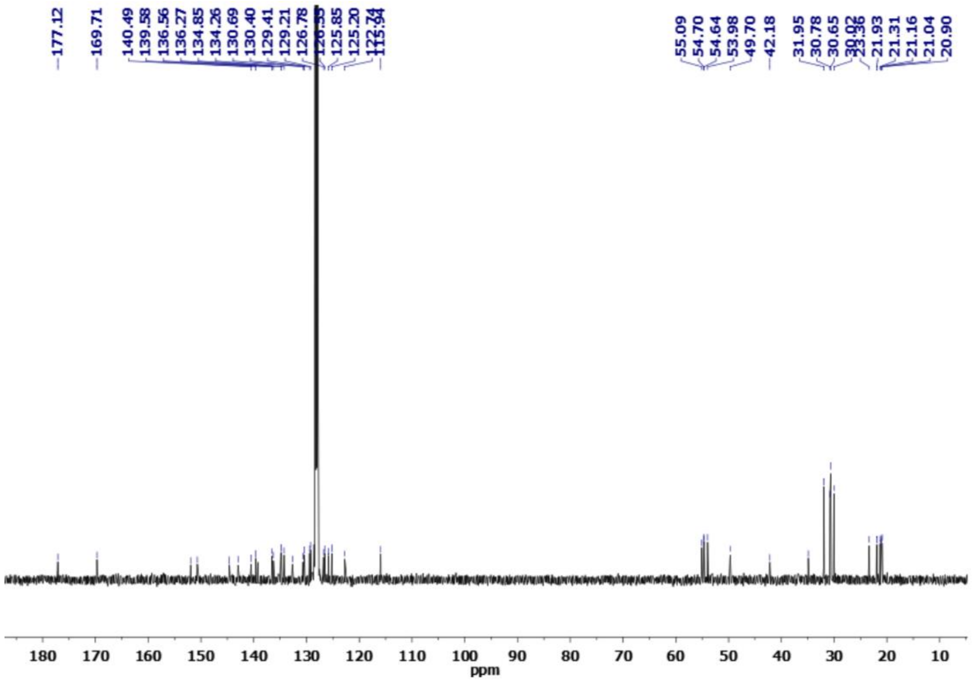

Figure S16. $^{13}C\{^{1}H\}$ NMR spectrum of compound **2*Mes*** in C_6D_6 under 1 bar N_2 at 298 K.

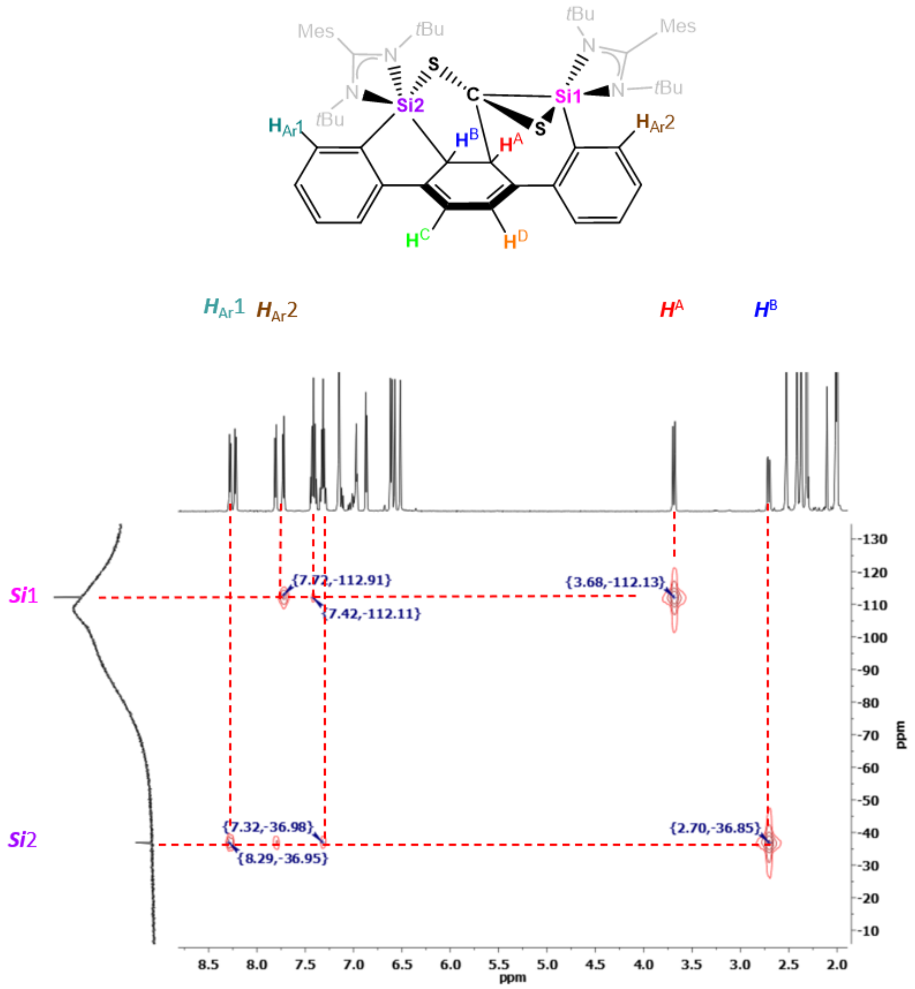

Figure S17. $^{1}H,^{29}Si$ HMQC NMR spectrum (optimized for $J_{Si,H}$ = 7 Hz) of compound **2*Mes*** in C_6D_6 under 1 bar N_2 at 298 K.

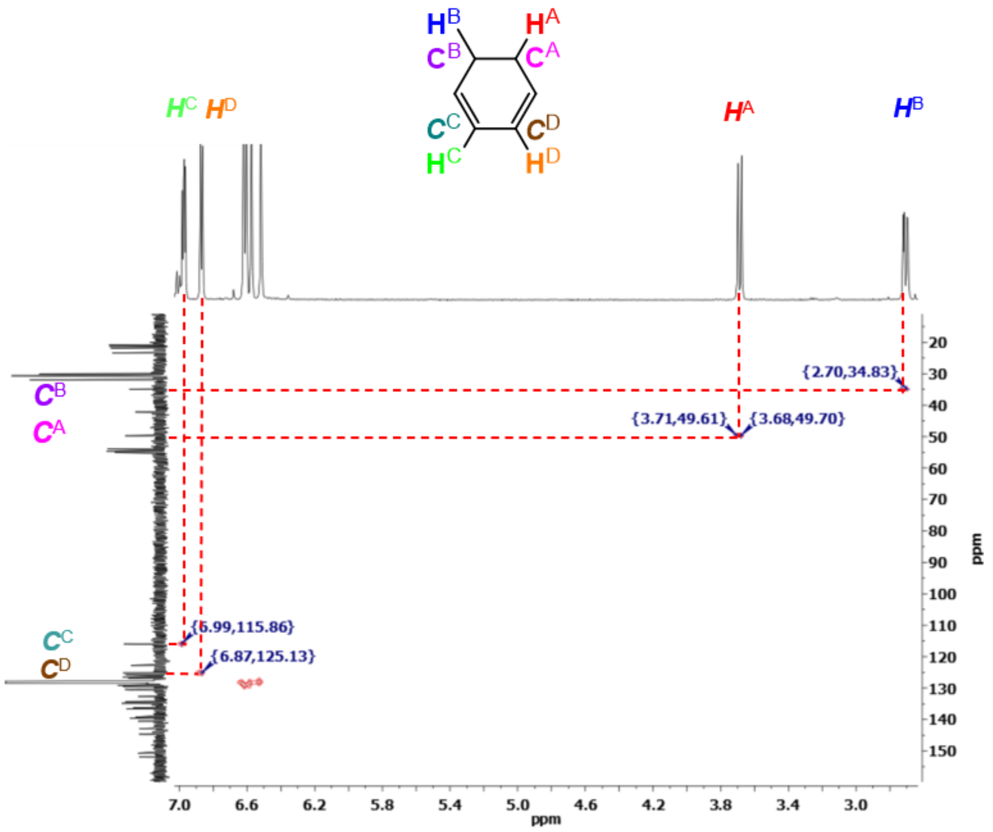

Figure S18. $^{1}H,^{13}C$ HSQC NMR spectrum of compound **2*Mes*** in C_6D_6 under 1 bar N_2 at 298 K.

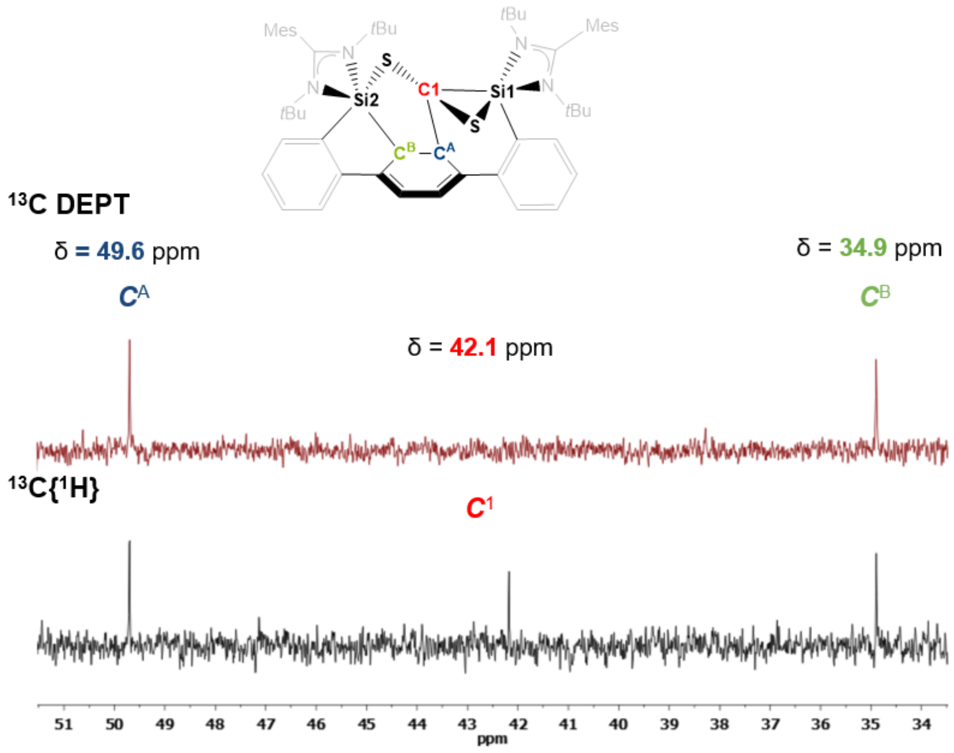

Figure S19. Section of $^{13}C\{^{1}H\}$ NMR spectrum (bottom) and ^{13}C 135 DEPT spectrum (top) of compound **2*Mes***.

3.5 Compound 3

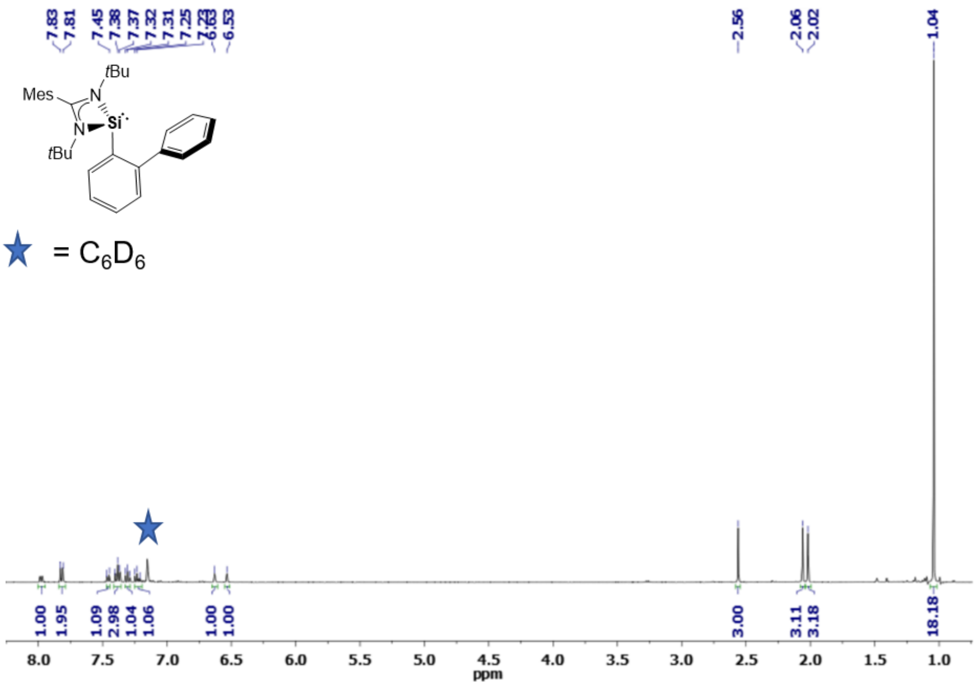

Figure S20. ^{1}H NMR spectrum of compound **3** in C_6D_6 under 1 bar N_2 at 298 K.

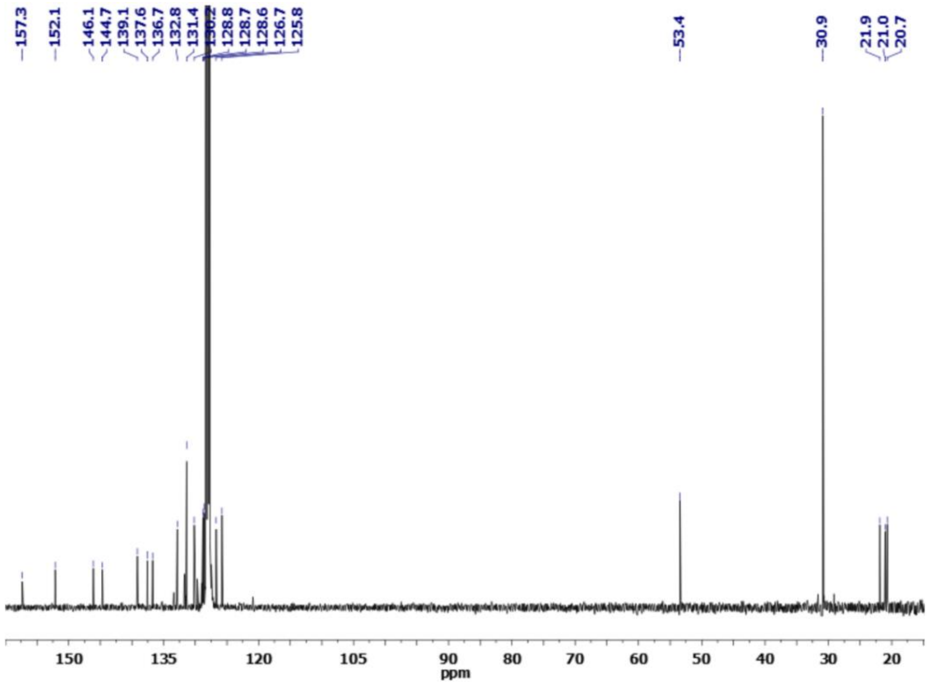

Figure S21. $^{13}C\{^{1}H\}$ NMR spectrum of compound **3** in C_6D_6 under 1 bar N_2 at 298 K.

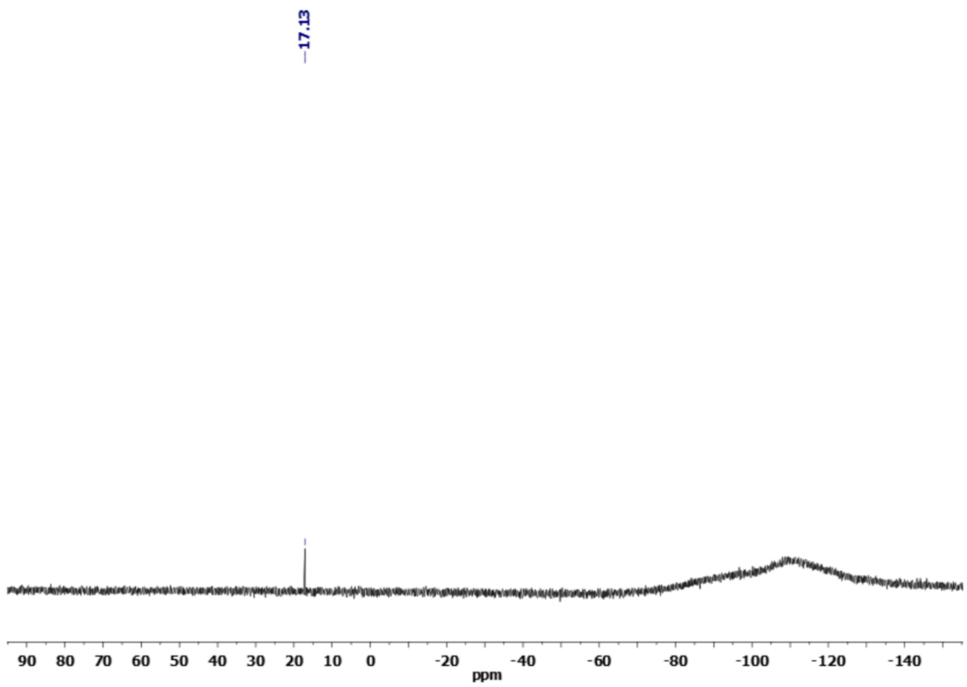

Figure S22. $^{29}Si\{^{1}H\}$ NMR spectrum of compound **3** in C_6D_6 under 1 bar N_2 at 298 K.

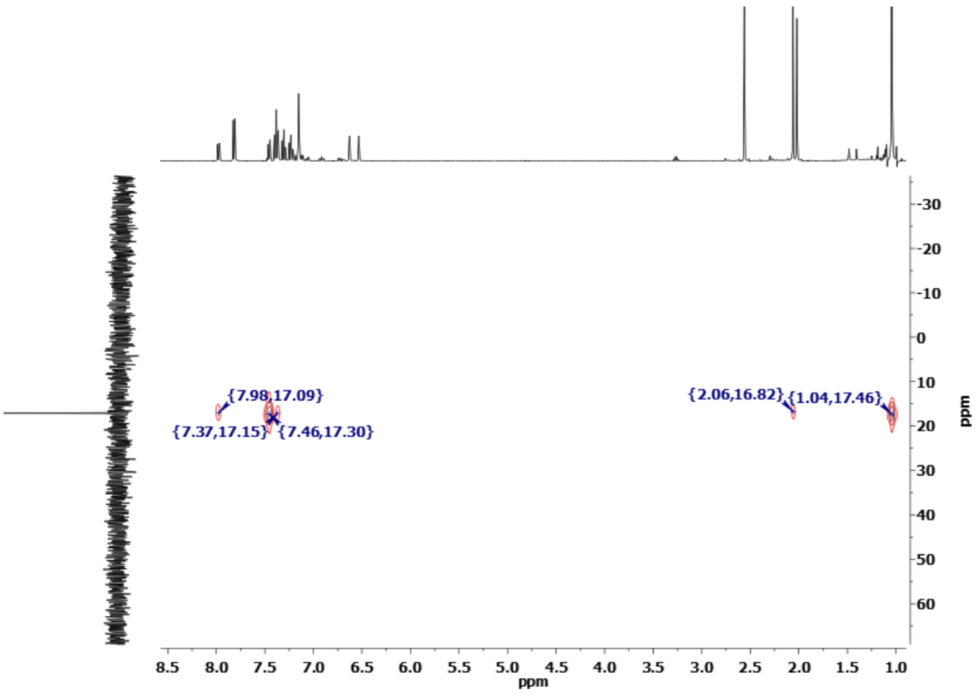

Figure S23. $^{1}H,^{29}Si$ NMR spectrum (optimized for $J_{Si,H}$ = 7 Hz) of compound **3** in C_6D_6 under 1 bar N_2 at 298 K.

3.6 Compound 4

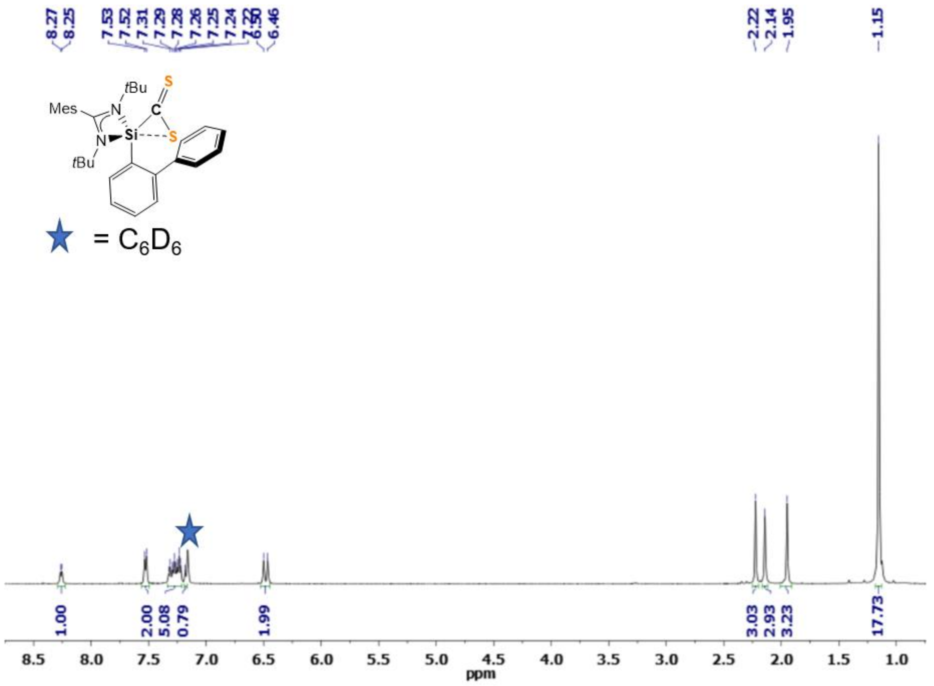

Figure S24. ^{1}H NMR spectrum of compound **4** in C_6D_6 under 1 bar N_2 at 298 K.

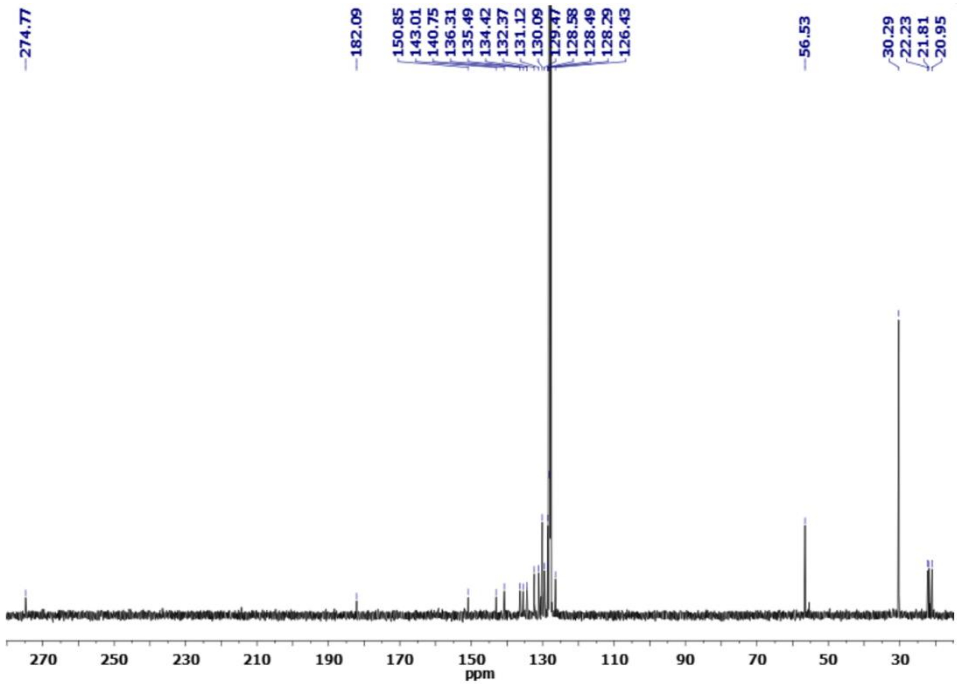

Figure S25. ^{13}C NMR spectrum of compound **4** in C_6D_6 under 1 bar N_2 at 298 K.

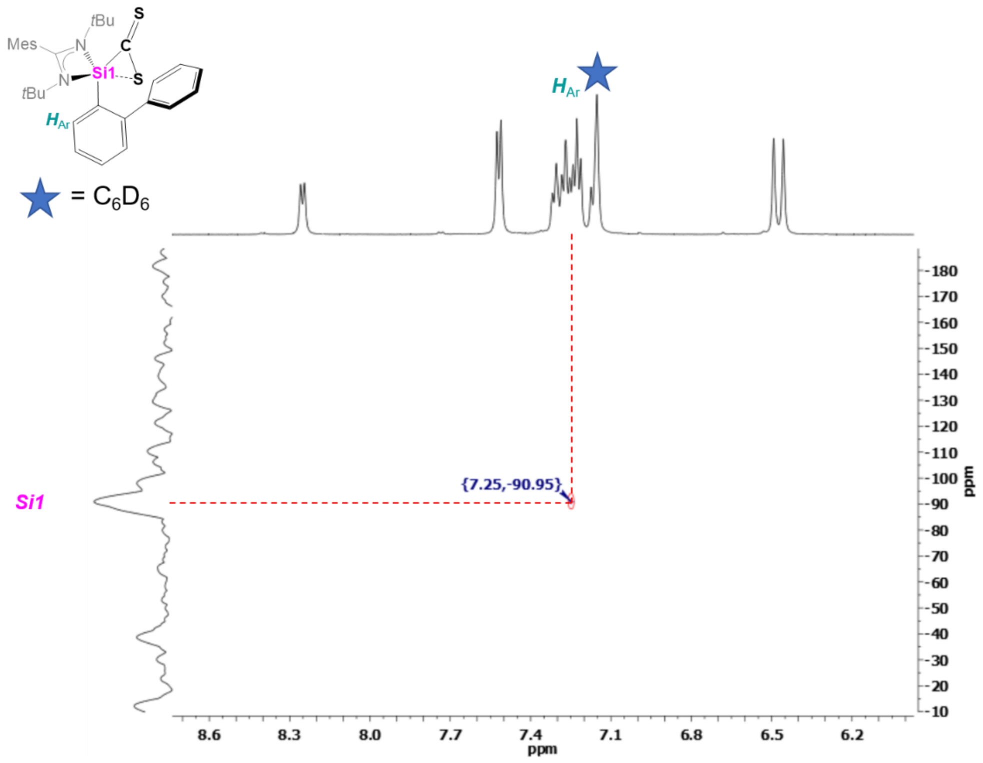

Figure 26. ^{1}H,^{29}Si NMR spectrum (optimized for $J_{Si,H}$ = 5 Hz) of compound **4** in C_6D_6 under 1 bar N_2 at 298 K.

3.7 Compound 6

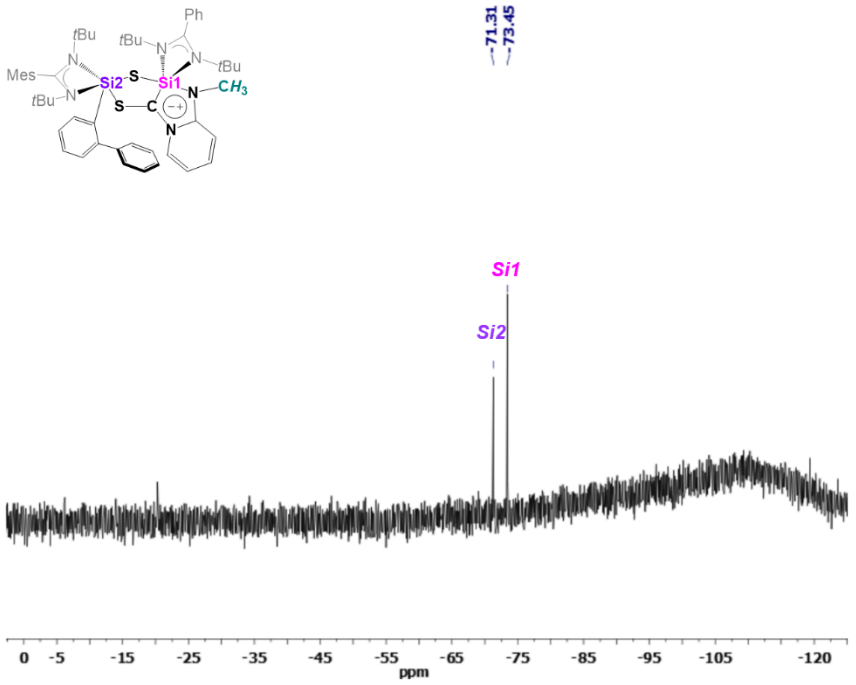

Figure S27. $^{29}Si\{^{1}H\}$ NMR spectrum of compound **6** in Tol-d_8 under 1 bar N_2 at 223 K.

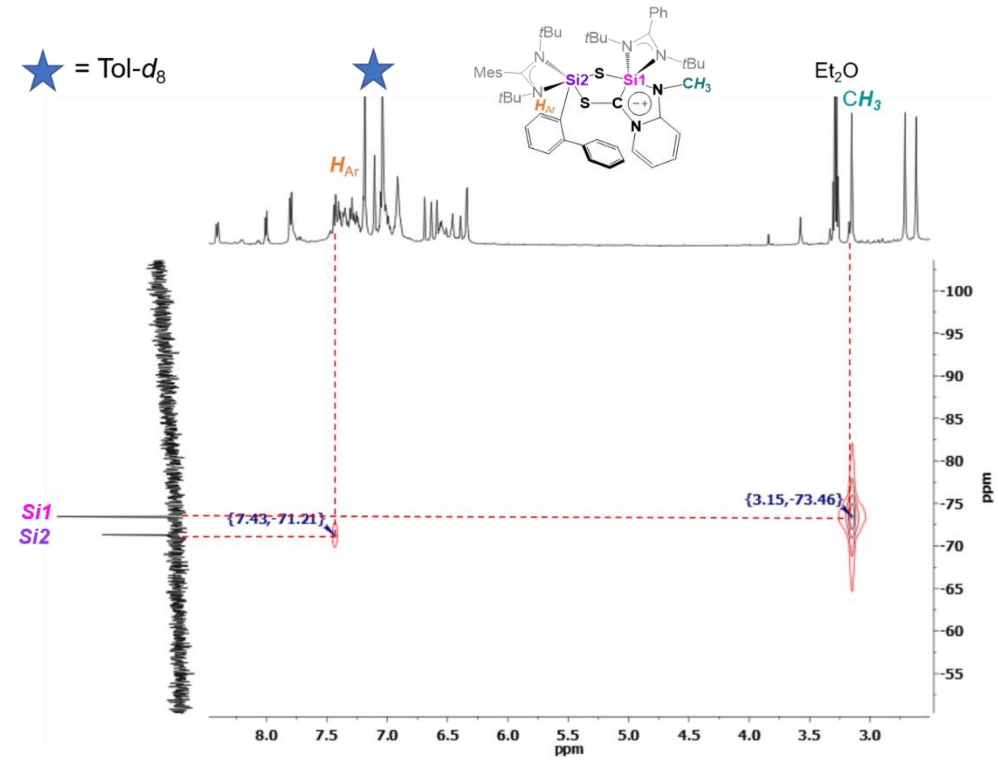

Figure S28. ^{1}H,^{29}Si HMQC NMR spectrum (optimized for $J_{Si,H}$ = 7 Hz) of compound **6** in Tol-d_8 under 1 bar N_2 at 223 K.

3.8 Compound 8

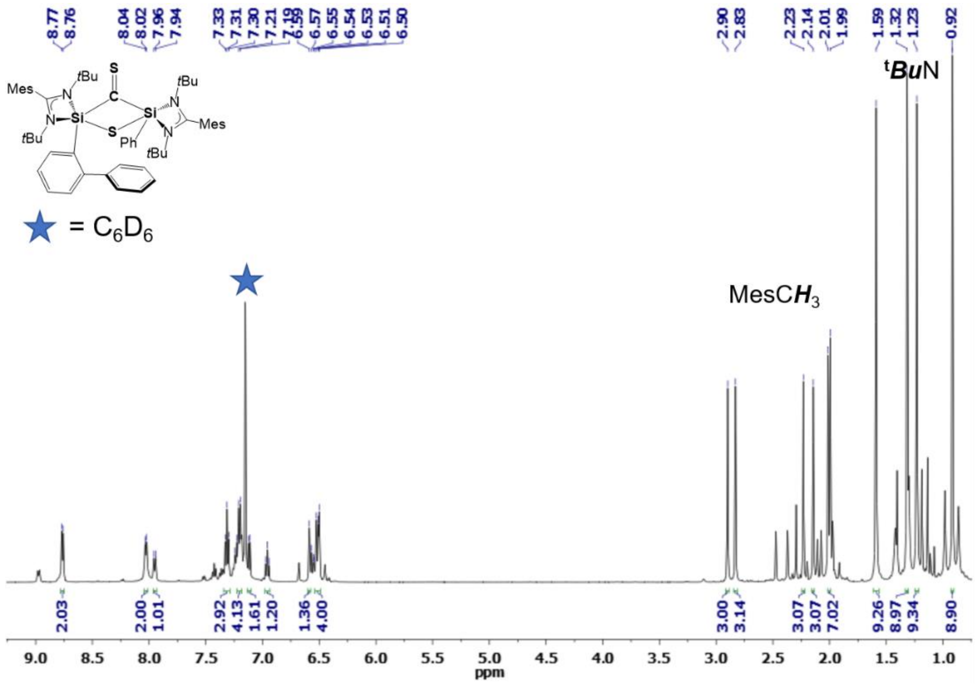

Figure S29. ^{1}H NMR spectrum of compound **8** in C_6D_6 under 1 bar N_2 at 298 K.

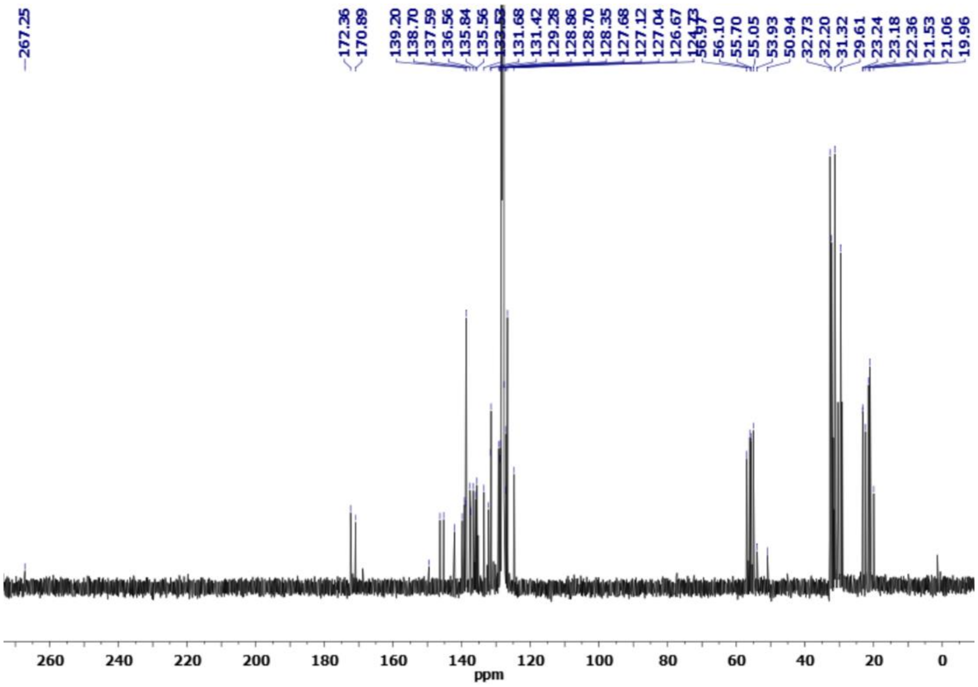

Figure S30. ^{13}C NMR spectrum of compound **8** in C_6D_6 under 1 bar N_2 at 298 K.

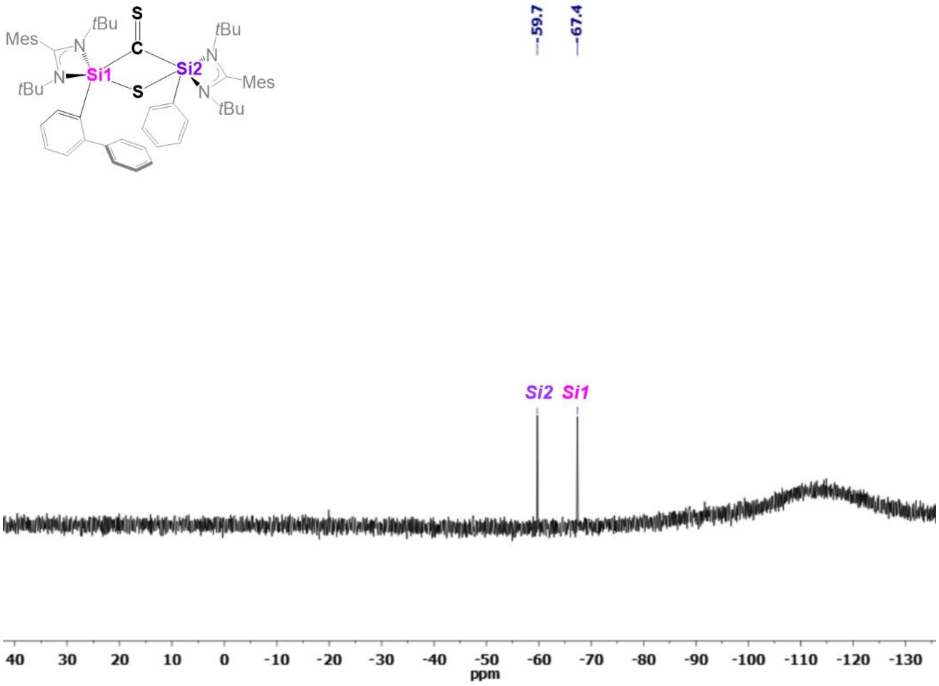

Figure S31. $^{29}Si\{^1H\}$ NMR spectrum of compound **8** in C_6D_6 under 1 bar N_2 at 298 K.

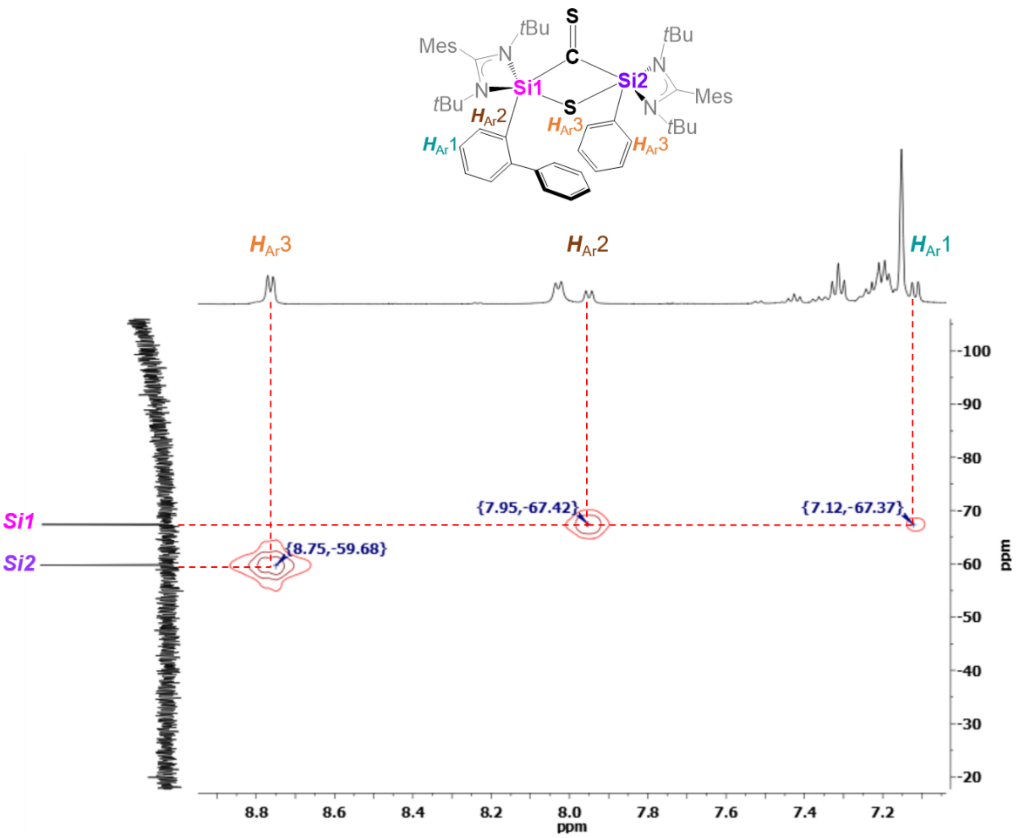

Figure S32. $^{1}H,^{29}Si$ HMQC NMR spectrum (optimized for $J_{Si,H}$ = 7 Hz) of compound **8** in C_6D_6 under 1 bar N_2 at 298 K.

3.9 Compound 10

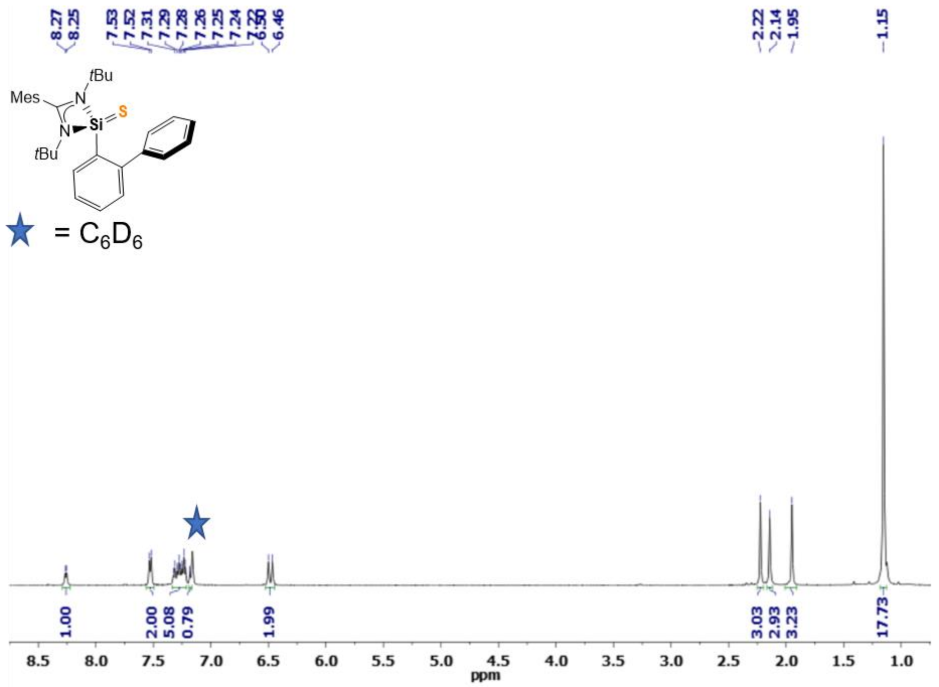

Figure S33. ^{1}H NMR spectrum of compound **10** in C_6D_6 under 1 bar N_2 at 298 K.

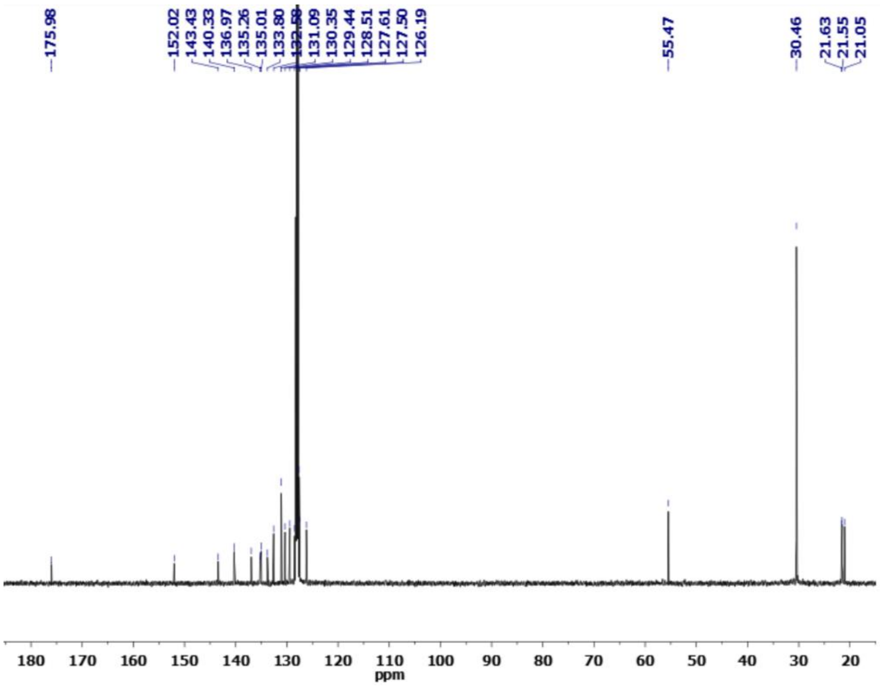

Figure S34. $^{13}C\{^1H\}$ NMR spectrum of compound **10** in C_6D_6 under 1 bar N_2 at 298 K.

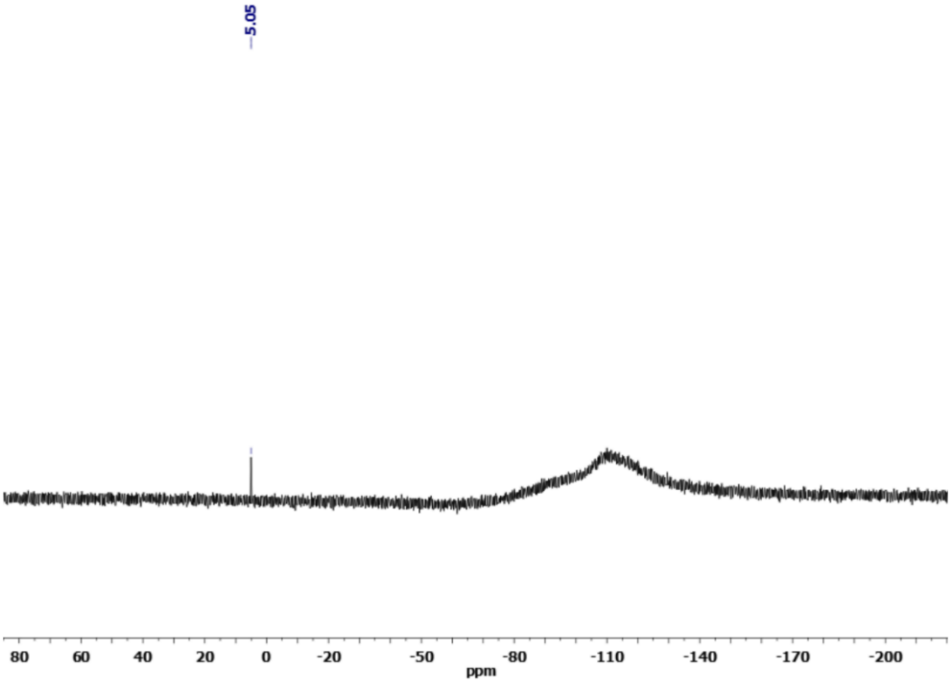

Figure S35. $^{29}Si\{^1H\}$ NMR spectrum of compound **10** in C_6D_6 under 1 bar N_2 at 298 K.

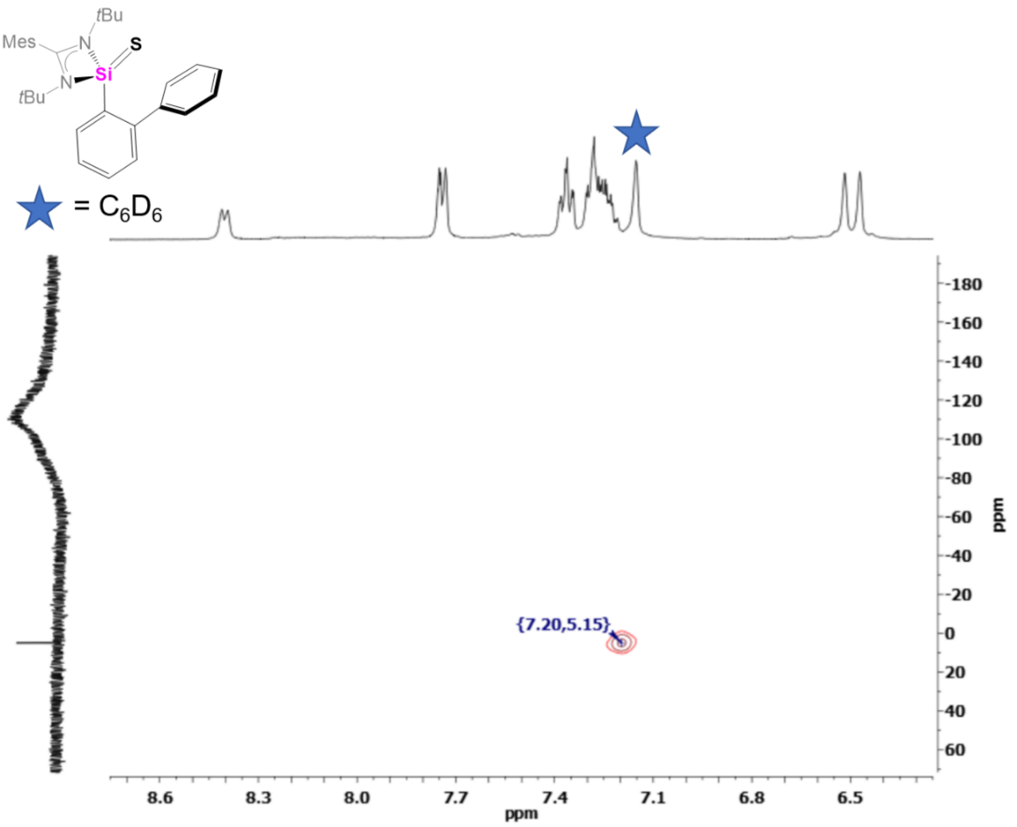

Figure S36. $^{1}H,^{29}Si$ NMR spectrum (optimized for $J_{Si,H}$ = 4 Hz) of compound **10** in C_6D_6 under 1 bar N_2 at 298 K.

4. FT-IR Spectra

4.1 Compound 1^{Mes}

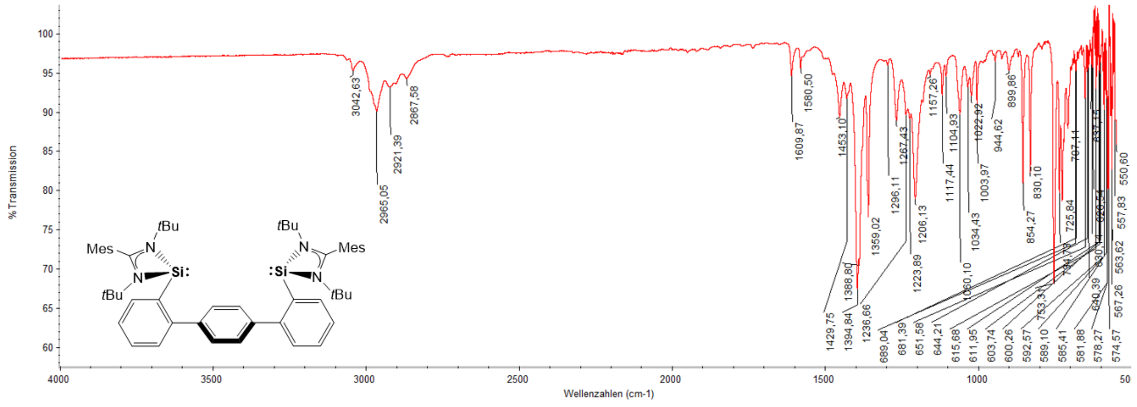

Figure S37. Solid state FT-IR spectrum of compound **1**^{Mes}.

4.2 Compound 2^{Ph}

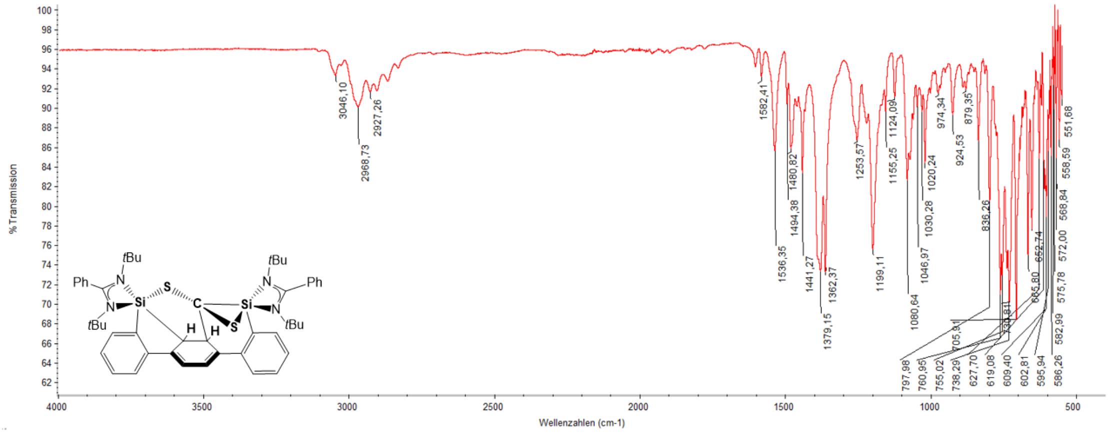

Figure S38. Solid state FT-IR spectrum of compound $\mathbf{2}^{Ph}$.

4.3 Compound 2^{Mes}

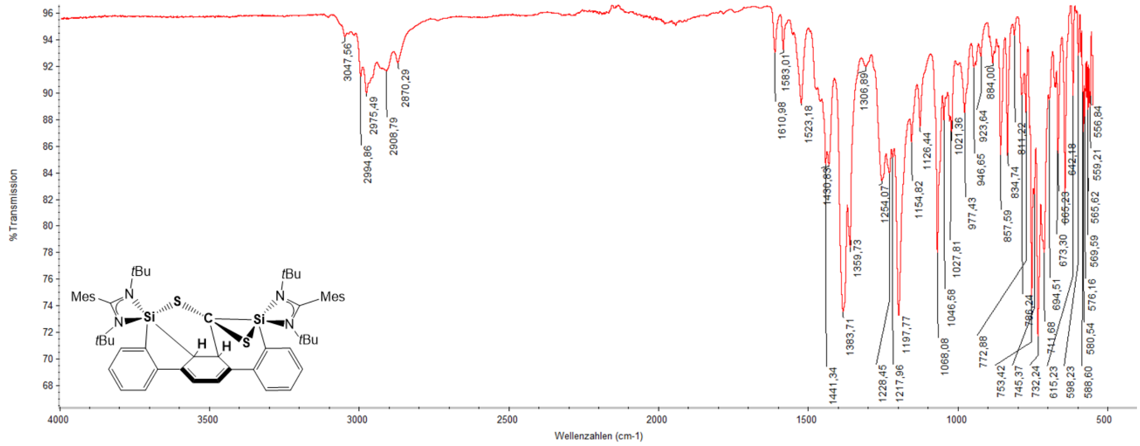

Figure S39. Solid state FT-IR spectrum of compound $\mathbf{2}^{Mes}$.

4.4 Compound 3

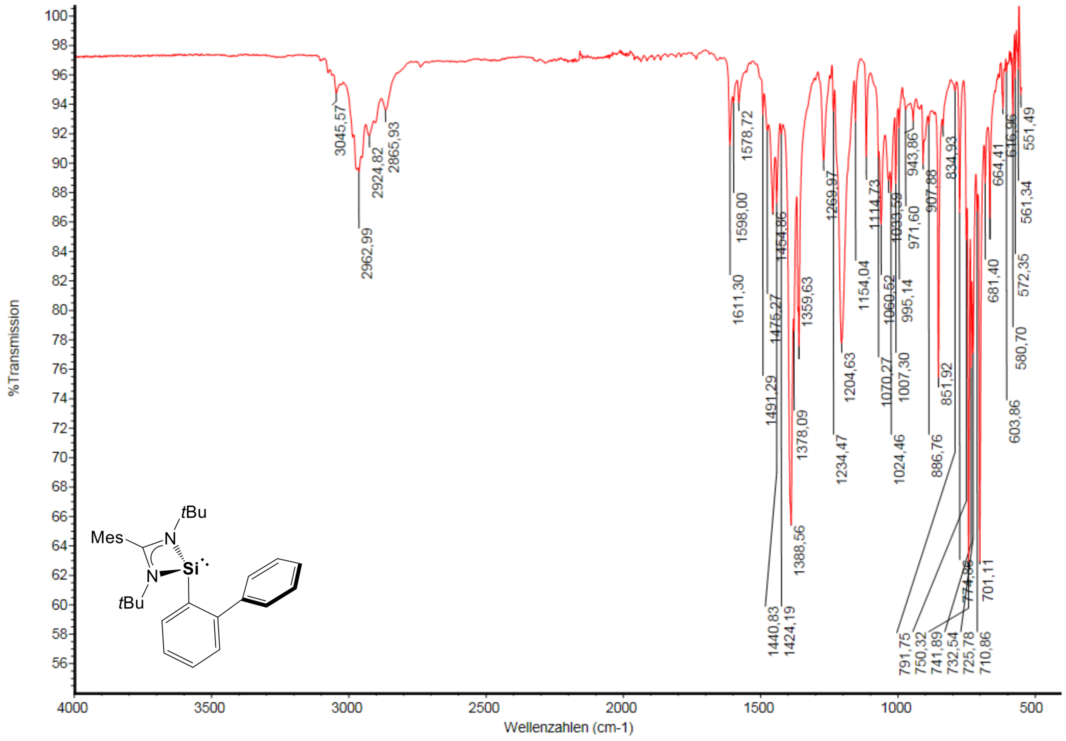

Figure S40. Solid state FT-IR spectrum of compound **XX**.

4.5 Compound 4

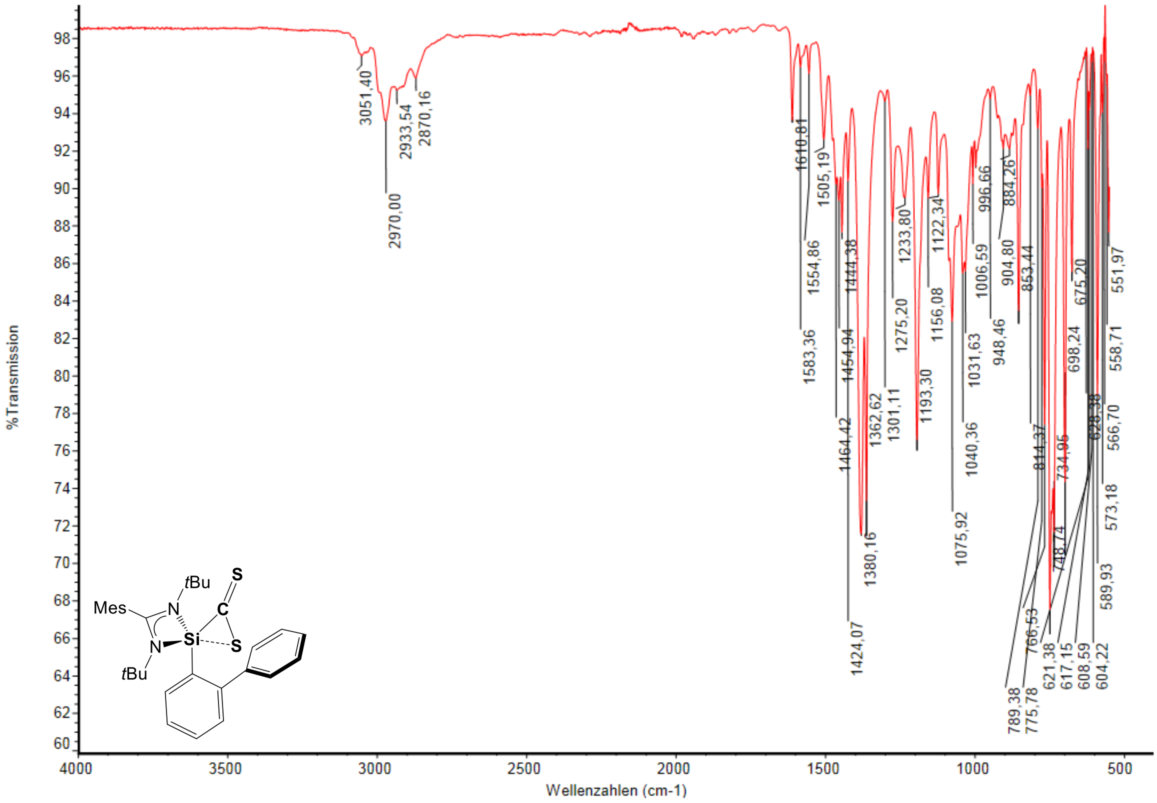

Figure S41. Solid state FT-IR spectrum of compound **4**.

4.6 Compound 6

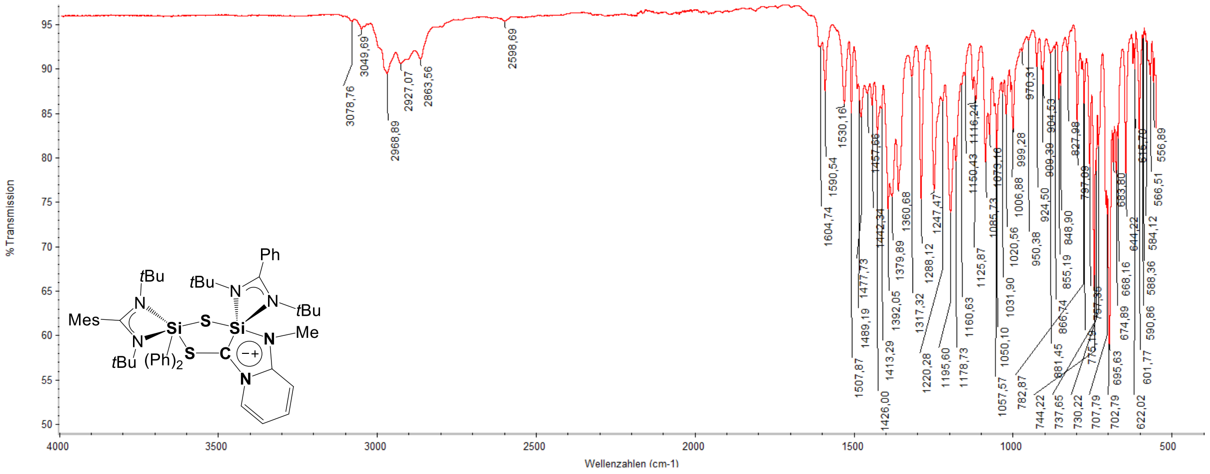

Figure S42. Solid state FT-IR spectrum of compound **6**.

4.7 Compound 7

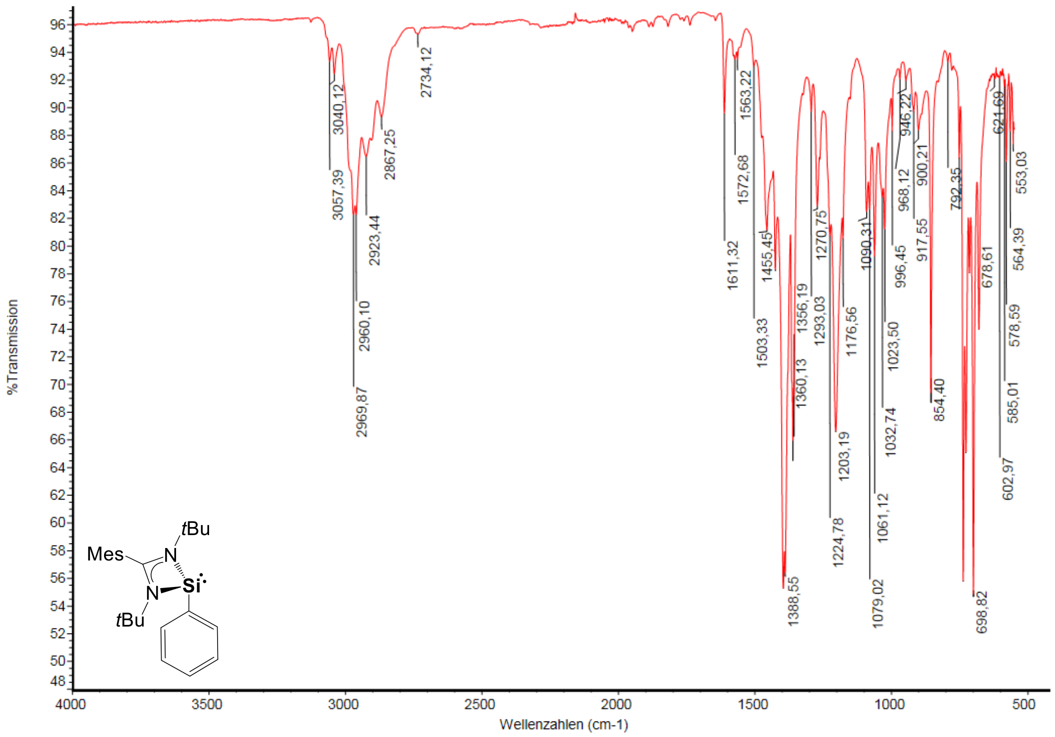

Figure S43. Solid state FT-IR spectrum of compound **7**.

4.8 Compound 8

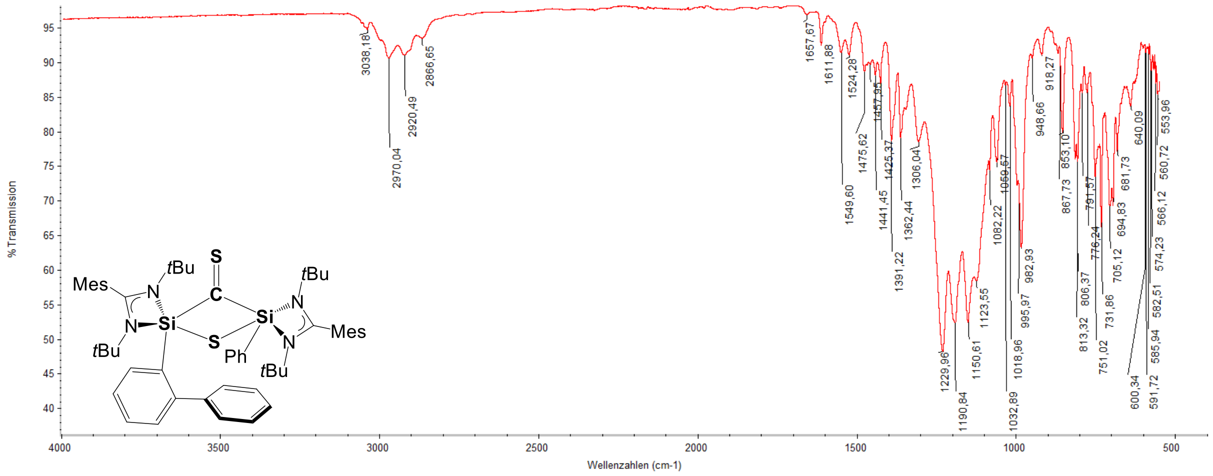

Figure S44. Solid state FT-IR spectrum of compound **8**.

5. Calculations

5.1 Compound 2 – DFT

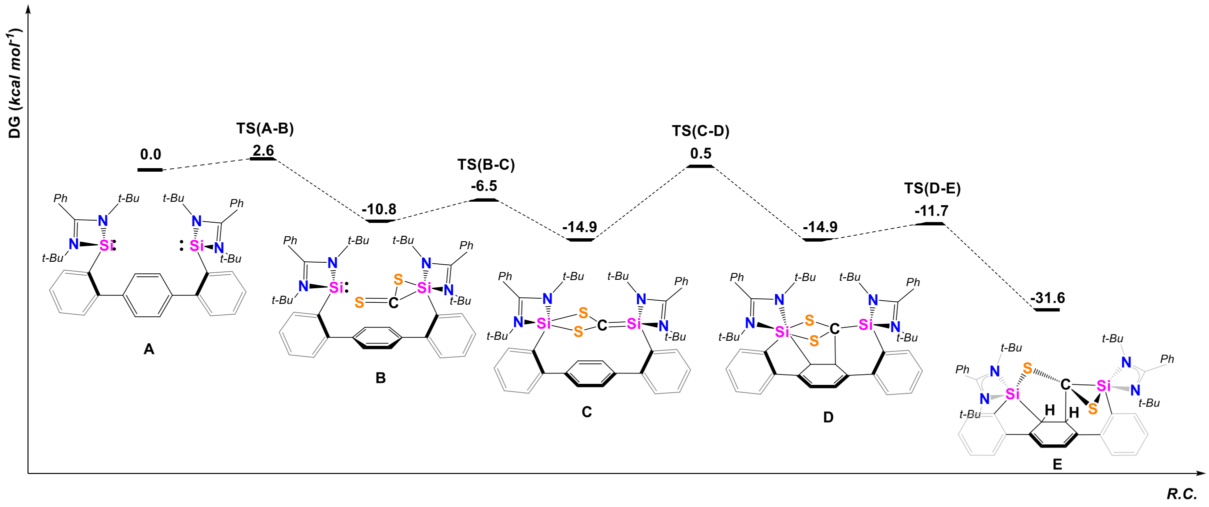

Figure S45. Calculated potential energy surface of the metal free intramolecular de-aromatization.

All quantum chemical calculations were carried out using Gaussian 09.[7] The geometries of all compounds were optimized at B3LYP[8] -D3[9]/def2-SVP[10]basis set for all other atoms. Analytical frequencies were computed to verify the stationary points. Thermochemistry at 298.15 K.

Calculated cartesian coordinates and energies of A

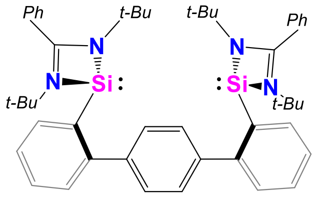

A

E(B3LYP)	-2660.579296 E_h
Sum of electronic and zero-point Energies	-2659.629404 E_h
Sum of electronic and thermal Energies	-2659.575030 E_h
Sum of electronic and thermal Enthalpies	-2659.574086 E_h
Sum of electronic and thermal Free Energies	-2659.719427 E_h

Number of imaginary frequencies: 0.

Si	-2.91897	0.87777	0.54007
Si	1.62058	1.75632	-0.34069
N	-2.74520	-0.40434	-0.87795
N	-3.99651	-0.68089	0.84650
N	3.17950	0.72627	-0.77730
N	1.94554	0.36744	0.94429
C	3.94713	0.59831	-2.02739
C	-0.78695	4.25755	1.55799
C	-2.12107	4.01404	1.23019
C	-3.43543	-1.32093	-0.19486
C	2.42613	3.27393	0.57732
C	2.90640	-0.16937	0.18799
C	-3.89115	3.44533	-0.48360
C	1.63451	4.40684	0.90435
C	-2.53269	3.94642	-0.11115
C	-4.22773	2.08460	-0.25273
C	-4.64130	-1.19735	2.06585
C	-2.02570	-0.46589	-2.16159
C	-0.25135	4.46603	-0.78130
C	-1.58965	4.24309	-1.10810
C	-5.49925	1.64593	-0.66865

C	0.18083	4.42017	0.55415
C	1.30629	-0.07882	2.19365
C	3.77508	3.28004	0.98030
C	-6.06018	3.83772	-1.52061
C	-4.80328	4.30021	-1.12510
C	-6.41152	2.50406	-1.28849
C	2.19464	5.47830	1.62001
C	4.33379	4.35518	1.67644
C	3.53857	5.45956	1.99910
H	-5.77438	0.59850	-0.51131
H	-7.39306	2.13286	-1.59768
H	-6.76419	4.51750	-2.00850
H	-4.51992	5.34094	-1.30568
H	4.39877	2.41095	0.74999
H	5.38628	4.33065	1.97389
H	3.96414	6.30553	2.54592
H	1.56508	6.33606	1.87285
H	-0.47318	4.24484	2.60471
H	-2.84752	3.81845	2.02227
H	0.48258	4.62223	-1.57532
H	-1.89646	4.22046	-2.15671
C	-2.89384	-1.13967	-3.23970
H	-3.87723	-0.64781	-3.29963
H	-2.40265	-1.05159	-4.22143
H	-3.04806	-2.20839	-3.03743
C	-0.68296	-1.20900	-2.01151
H	-0.06247	-0.71622	-1.24878
H	-0.83691	-2.26091	-1.73054
H	-0.12829	-1.18868	-2.96300
C	-1.75288	0.99026	-2.57410
H	-1.20171	1.01796	-3.52677
H	-2.69665	1.54224	-2.69685
H	-1.13392	1.50104	-1.82040
C	-5.81616	-2.13363	1.72989

H	-5.47519	-3.08011	1.29050
H	-6.37595	-2.37310	2.64757
H	-6.50671	-1.64892	1.02221
C	-3.62073	-1.92439	2.96369
H	-4.08902	-2.23222	3.91255
H	-3.23041	-2.82632	2.47038
H	-2.77416	-1.25834	3.19022
C	-5.18769	0.03452	2.81000
H	-4.37212	0.73062	3.06469
H	-5.91612	0.57588	2.18783
H	-5.67958	-0.26835	3.74686
C	0.56160	1.14355	2.75679
H	-0.20049	1.49897	2.04639
H	0.04264	0.87550	3.68996
H	1.26469	1.96342	2.96648
C	0.29242	-1.20857	1.92305
H	-0.23089	-1.48182	2.85304
H	-0.46212	-0.87173	1.19705
H	0.79175	-2.10945	1.53787
C	2.36149	-0.53924	3.21540
H	1.87888	-0.73133	4.18652
H	2.86342	-1.46486	2.90245
H	3.12358	0.24285	3.35770
C	3.26715	-0.39024	-2.99492
H	3.79757	-0.41489	-3.96054
H	3.26569	-1.41002	-2.58377
H	2.22435	-0.08719	-3.17556
C	3.96345	2.00179	-2.65939
H	2.93813	2.34839	-2.86697
H	4.43902	2.72825	-1.98344
H	4.51727	1.98948	-3.61050
C	5.39862	0.16315	-1.75407
H	5.86511	0.82331	-1.00627
H	5.45621	-0.87109	-1.39036

H	5.98771	0.22507	-2.68258
C	-3.42829	-2.79409	-0.43917
C	-2.34931	-3.56734	0.01747
C	-4.48215	-3.41977	-1.12078
C	-2.32886	-4.94726	-0.20172
H	-1.52775	-3.08175	0.54786
C	-4.45784	-4.79864	-1.34381
H	-5.32080	-2.81845	-1.47833
C	-3.38203	-5.56522	-0.88350
H	-1.48667	-5.54126	0.16211
H	-5.28238	-5.27656	-1.87851
H	-3.36474	-6.64419	-1.05648
C	3.43707	-1.56120	0.29197
C	4.66303	-1.82167	0.92110
C	2.69626	-2.62849	-0.23980
C	5.14147	-3.13053	1.01893
H	5.23757	-0.99177	1.33810
C	3.17827	-3.93666	-0.14538
H	1.74243	-2.42739	-0.73176
C	4.40064	-4.19035	0.48518
H	6.09617	-3.32384	1.51429
H	2.59647	-4.76034	-0.56671
H	4.77618	-5.21387	0.56099

Calculated cartesian coordinates and energies of TS(A-B)

E(B3LYP)	-3494.831523 E_h
Sum of electronic and zero-point Energies	-3493.872902 E_h
Sum of electronic and thermal Energies	-3493.815017 E_h
Sum of electronic and thermal Enthalpies	-3493.814072 E_h
Sum of electronic and thermal Free Energies	-3493.966023 E_h

Number of imaginary frequencies: 1, v = -164.9 cm^{-1}.

S	-0.22176	-1.69461	0.93309
S	-0.10426	0.47401	3.16218

Si	2.56126	0.88035	-0.23202
Si	-2.37824	1.00573	0.40417
N	3.73953	-0.02652	0.97447
N	3.57367	-0.49646	-1.11545
N	-3.76074	-0.25865	0.70284
N	-2.68531	-0.02489	-1.14383
C	-0.37484	-0.42374	1.88049
C	-4.46403	-0.75331	1.90149
C	-0.61680	4.44892	-1.43757
C	0.77181	4.42400	-1.57776
C	4.04115	-0.95308	0.05837
C	-3.37894	2.62710	0.04065
C	-3.54697	-0.81895	-0.49847
C	3.05288	3.72911	-0.77631
C	-2.72604	3.86349	-0.21340
C	1.58993	3.91467	-0.55649
C	3.64969	2.44910	-0.63081
C	3.36370	-1.18401	-2.40067
C	4.16633	0.12772	2.37516
C	-0.41475	3.53697	0.78333
C	0.97242	3.51583	0.64232
C	5.03251	2.33928	-0.87311
C	-1.23832	3.96439	-0.27266
C	-2.14094	-0.05521	-2.51153
C	-4.78754	2.60876	0.00277
C	5.20473	4.69052	-1.40080
C	3.83654	4.83102	-1.16953
C	5.80913	3.43693	-1.24870
C	-3.49065	5.01055	-0.49681
C	-5.53849	3.75558	-0.26659
C	-4.88499	4.96514	-0.51632
H	5.51024	1.35911	-0.77372
H	6.88198	3.31891	-1.42714
H	5.80119	5.55864	-1.69476

H	3.36184	5.81046	-1.27623
H	-5.30965	1.66484	0.17328
H	-6.63077	3.70349	-0.28617
H	-5.45874	5.87218	-0.72488
H	-2.97394	5.95399	-0.69209
H	-1.23774	4.79407	-2.26842
H	1.22837	4.74730	-2.51684
H	-0.85812	3.20120	1.72304
H	1.58860	3.17671	1.47595
C	5.70483	0.12210	2.45595
H	6.12502	0.89406	1.79291
H	6.03260	0.33206	3.48646
H	6.11859	-0.85393	2.16500
C	3.57786	-0.96932	3.28359
H	2.48273	-1.00358	3.18564
H	3.98614	-1.95941	3.04036
H	3.82167	-0.75512	4.33664
C	3.64771	1.49782	2.84481
H	3.94305	1.67804	3.88939
H	4.05574	2.30756	2.22182
H	2.54934	1.53036	2.79349
C	4.69155	-1.72647	-2.95970
H	5.08702	-2.54816	-2.34818
H	4.53943	-2.11165	-3.98043
H	5.44589	-0.92531	-3.00055
C	2.33264	-2.32084	-2.26115
H	2.07064	-2.73169	-3.24946
H	2.72474	-3.14249	-1.64519
H	1.41722	-1.93952	-1.78511
C	2.81873	-0.11813	-3.36886
H	1.85651	0.28045	-3.01218
H	3.52531	0.72070	-3.46100
H	2.65725	-0.55330	-4.36706
C	-1.02685	1.00265	-2.55407

H	-0.22638	0.77171	-1.83278
H	-0.58436	1.04883	-3.56049
H	-1.41978	2.00117	-2.31515
C	-1.54234	-1.42730	-2.87134
H	-0.98924	-1.35267	-3.82056
H	-0.84211	-1.75186	-2.08873
H	-2.31465	-2.19830	-2.99223
C	-3.25438	0.32525	-3.50508
H	-2.85130	0.38906	-4.52835
H	-4.05744	-0.42689	-3.50268
H	-3.68800	1.30086	-3.23670
C	-3.84054	-2.05841	2.43235
H	-4.31202	-2.33856	3.38762
H	-3.98838	-2.88947	1.72928
H	-2.76155	-1.93274	2.59941
C	-4.31198	0.34437	2.96999
H	-3.25021	0.50371	3.21718
H	-4.73499	1.29819	2.62037
H	-4.83468	0.05141	3.89294
C	-5.95885	-0.96963	1.60157
H	-6.42093	-0.04723	1.21747
H	-6.11082	-1.76816	0.86240
H	-6.48858	-1.25859	2.52283
C	4.59774	-2.31063	0.33259
C	3.74311	-3.31042	0.82474
C	5.95025	-2.60648	0.11320
C	4.23747	-4.58994	1.08732
H	2.69063	-3.07452	1.00142
C	6.44437	-3.88593	0.38269
H	6.61398	-1.82850	-0.27045
C	5.58868	-4.87974	0.86809
H	3.56548	-5.36313	1.46813
H	7.50089	-4.10710	0.21209
H	5.97513	-5.88047	1.07714

C	-4.11272	-2.10379	-1.00182
C	-5.33094	-2.10379	-1.69663
C	-3.43107	-3.31349	-0.80029
C	-5.86242	-3.30077	-2.18497
H	-5.86259	-1.16198	-1.85020
C	-3.96568	-4.50814	-1.28702
H	-2.47926	-3.30697	-0.26643
C	-5.18113	-4.50440	-1.98026
H	-6.81168	-3.29211	-2.72615
H	-3.42894	-5.44640	-1.12659
H	-5.59676	-5.44036	-2.36180

Calculated cartesian coordinates and energies of B

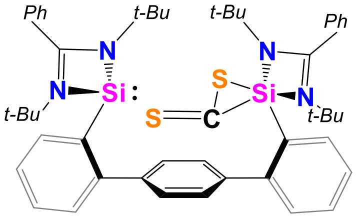

B

E(B3LYP)	-3494.866599 E_h
Sum of electronic and zero-point Energies	-3493.906109 E_h
Sum of electronic and thermal Energies	-3493.848358 E_h
Sum of electronic and thermal Enthalpies	-3493.847414 E_h
Sum of electronic and thermal Free Energies	-3493.999178 E_h

Number of imaginary frequencies: 0.

S	0.27046	-1.28136	0.17882
S	0.33866	1.02847	-1.83232
Si	-2.66637	0.61960	0.19204
Si	2.56338	0.77715	-0.08890
N	-3.64389	-0.47467	-1.01275
N	-3.63411	-0.74399	1.11506
N	3.76466	-0.29144	-1.01100
N	3.45568	-0.30588	1.12421
C	0.89317	0.11429	-0.52288
C	4.04272	-0.58073	-2.43930
C	0.34055	3.05443	1.26061
C	-1.04923	3.01688	1.36276
C	-3.92340	-1.35679	-0.04448
C	3.20299	2.53458	0.00511
C	4.02979	-0.97073	0.11466
C	-3.34173	3.45153	0.41044
C	2.44181	3.73022	0.06126
C	-1.86381	3.64990	0.40623
C	-3.87489	2.13727	0.37819
C	-3.62609	-1.23240	2.50008
C	-3.70565	-0.56451	-2.48027

C	0.15640	4.42851	-0.71194
C	-1.23515	4.38292	-0.61226
C	-5.27393	1.99402	0.35731
C	0.96361	3.73438	0.20249
C	3.39658	-0.57544	2.57804
C	4.61020	2.63079	-0.05766
C	-5.58790	4.39072	0.37834
C	-4.20347	4.56391	0.39648
C	-6.12833	3.09885	0.36378
C	3.11436	4.96523	-0.00226
C	5.26215	3.86266	-0.09438
C	4.50463	5.03757	-0.08116
H	-5.69996	0.98542	0.32263
H	-7.21335	2.95872	0.35060
H	-6.24720	5.26329	0.37959
H	-3.77744	5.57120	0.41557
H	5.21051	1.71822	-0.10225
H	6.35307	3.90575	-0.14428
H	4.99815	6.01226	-0.11919
H	2.52291	5.88301	0.03772
H	0.94807	2.55920	2.01830
H	-1.51447	2.49926	2.20318
H	0.62420	4.95851	-1.54509
H	-1.84927	4.87269	-1.37214
C	-5.08671	-1.07125	-2.93207
H	-5.88618	-0.44774	-2.50138
H	-5.16197	-1.02066	-4.02985
H	-5.25769	-2.11433	-2.63218
C	-2.58677	-1.46850	-3.03284
H	-1.61086	-1.13688	-2.64823
H	-2.74445	-2.51771	-2.74525
H	-2.56899	-1.41919	-4.13393
C	-3.50070	0.86992	-3.00154
H	-3.54962	0.88585	-4.10136

H	-4.27474	1.54373	-2.60365
H	-2.51282	1.25155	-2.70000
C	-5.03671	-1.69434	2.91007
H	-5.34815	-2.58271	2.34332
H	-5.05744	-1.95386	3.98069
H	-5.76865	-0.89117	2.73172
C	-2.60474	-2.36839	2.70888
H	-2.53334	-2.62375	3.77883
H	-2.89595	-3.27756	2.16502
H	-1.61164	-2.05479	2.35308
C	-3.21954	-0.03018	3.37188
H	-2.20268	0.30475	3.11126
H	-3.91138	0.81260	3.22179
H	-3.22600	-0.30676	4.43728
C	2.34662	0.39513	3.14142
H	1.36502	0.20432	2.67948
H	2.25112	0.25974	4.22879
H	2.64146	1.44040	2.95754
C	2.95860	-2.01801	2.88477
H	2.76279	-2.11675	3.96381
H	2.03666	-2.25126	2.33221
H	3.73376	-2.74908	2.61966
C	4.77132	-0.27468	3.20032
H	4.72415	-0.38274	4.29525
H	5.53593	-0.97153	2.82646
H	5.08725	0.75368	2.96470
C	2.99714	-1.56778	-2.99056
H	3.17095	-1.73248	-4.06565
H	3.06933	-2.53986	-2.48190
H	1.97996	-1.17332	-2.85676
C	3.93589	0.76424	-3.17991
H	2.93392	1.20291	-3.05941
H	4.67955	1.48197	-2.80123
H	4.11455	0.61176	-4.25492

C	5.46210	-1.14173	-2.62792
H	6.20849	-0.49991	-2.13363
H	5.56600	-2.16243	-2.23809
H	5.69503	-1.17070	-3.70309
C	-4.24717	-2.80154	-0.22987
C	-3.19470	-3.70892	-0.43530
C	-5.56807	-3.26955	-0.20576
C	-3.46675	-5.06735	-0.61366
H	-2.16743	-3.33537	-0.44871
C	-5.83717	-4.62949	-0.38611
H	-6.38566	-2.56195	-0.05061
C	-4.78719	-5.53011	-0.59038
H	-2.64320	-5.76830	-0.77227
H	-6.87019	-4.98592	-0.36848
H	-4.99785	-6.59330	-0.73187
C	4.72999	-2.27672	0.20799
C	6.09427	-2.35815	0.51215
C	3.98312	-3.44319	-0.01605
C	6.71484	-3.60801	0.58465
H	6.67124	-1.44560	0.67593
C	4.60941	-4.68858	0.05963
H	2.91339	-3.36103	-0.22401
C	5.97430	-4.77255	0.35724
H	7.78040	-3.67198	0.81739
H	4.02781	-5.59760	-0.11073
H	6.46184	-5.74885	0.41437

Calculated cartesian coordinates and energies of TS(B-C)

E(B3LYP)	-3494.863172 E_h
Sum of electronic and zero-point Energies	-3493.902655 E_h
Sum of electronic and thermal Energies	-3493.846028 E_h
Sum of electronic and thermal Enthalpies	-3493.845084 E_h
Sum of electronic and thermal Free Energies	-3493.992411 E_h

Number of imaginary frequencies: 1, v = -84.9 cm^{-1}.

S	-0.09755	-0.93804	0.38027
S	-0.09718	1.23520	-1.63633
Si	-2.38105	0.67833	0.01390
Si	2.40449	0.80287	-0.04645
N	-3.27791	-0.56738	-1.07586
N	-3.32567	-0.62019	1.07534
N	3.53765	-0.31679	-1.03325
N	3.21887	-0.41004	1.09732
C	0.70303	0.33199	-0.43470
C	3.67751	-0.60526	-2.47884
C	0.41262	3.05784	1.31023
C	-0.98217	3.01655	1.33112
C	-3.59002	-1.33599	-0.01929
C	3.23068	2.48653	0.03652
C	3.77565	-1.04909	0.05857
C	-3.19774	3.47899	0.24322
C	2.56456	3.72855	0.19758
C	-1.73946	3.73052	0.38002
C	-3.65529	2.14464	0.10552
C	-3.42716	-0.95165	2.50355
C	-3.36124	-0.82267	-2.52614
C	0.33447	4.58314	-0.55902
C	-1.06298	4.55374	-0.52749
C	-5.03743	1.93279	-0.05048
C	1.08790	3.80376	0.32920
C	3.23871	-0.68773	2.54963
C	4.63814	2.48540	-0.06896
C	-5.47749	4.30856	0.03854
C	-4.11222	4.54728	0.19417
C	-5.94531	2.99367	-0.07893
C	3.32099	4.91444	0.20610
C	5.37754	3.66783	-0.03337
C	4.71142	4.89045	0.09465
H	-5.40944	0.90932	-0.16202

H	-7.01563	2.80030	-0.19653
H	-6.17946	5.14671	0.01444
H	-3.74155	5.57117	0.29632
H	5.16606	1.53811	-0.20700
H	6.46697	3.63694	-0.11728
H	5.27630	5.82622	0.11546
H	2.79815	5.86687	0.32273
H	0.97846	2.51932	2.06908
H	-1.49257	2.45716	2.11418
H	0.84743	5.16319	-1.33005
H	-1.63403	5.10288	-1.28019
C	-4.64588	-1.59007	-2.89040
H	-5.53108	-1.10215	-2.45234
H	-4.76538	-1.59799	-3.98494
H	-4.62105	-2.63401	-2.55260
C	-2.12324	-1.60004	-3.01239
H	-1.20718	-1.06679	-2.72138
H	-2.09895	-2.60842	-2.57462
H	-2.14601	-1.70362	-4.10962
C	-3.41326	0.55674	-3.20994
H	-3.48031	0.43393	-4.30213
H	-4.28763	1.12880	-2.86441
H	-2.50776	1.13827	-2.98957
C	-4.91166	-1.07090	2.90002
H	-5.38371	-1.93601	2.41356
H	-5.01022	-1.20261	3.98962
H	-5.45782	-0.16136	2.60571
C	-2.66789	-2.24152	2.87150
H	-2.67306	-2.37780	3.96500
H	-3.12851	-3.13099	2.42167
H	-1.62286	-2.17900	2.53288
C	-2.79868	0.22474	3.27107
H	-1.73240	0.32012	3.01662
H	-3.30936	1.16797	3.02292

H	-2.88456	0.06099	4.35595
C	2.31880	0.36075	3.19396
H	1.29941	0.26760	2.78782
H	2.27732	0.20820	4.28256
H	2.69516	1.37948	3.01026
C	2.69200	-2.09092	2.86654
H	2.57369	-2.20145	3.95580
H	1.71101	-2.22188	2.38631
H	3.37119	-2.88280	2.52376
C	4.67196	-0.52182	3.08473
H	4.68383	-0.63565	4.18005
H	5.34511	-1.28132	2.66116
H	5.06493	0.47604	2.83417
C	2.56271	-1.56614	-2.93497
H	2.63075	-1.72951	-4.02221
H	2.65762	-2.54287	-2.43872
H	1.57316	-1.14687	-2.70298
C	3.51906	0.74572	-3.19871
H	2.53205	1.18948	-2.99314
H	4.29501	1.45683	-2.87695
H	3.60568	0.60166	-4.28613
C	5.06213	-1.19124	-2.80025
H	5.86229	-0.55315	-2.39283
H	5.18874	-2.20627	-2.40135
H	5.18904	-1.24328	-3.89243
C	-3.98138	-2.77343	-0.06417
C	-2.97378	-3.73750	-0.23067
C	-5.31681	-3.17983	0.05735
C	-3.30473	-5.09367	-0.27242
H	-1.93593	-3.40501	-0.30789
C	-5.64436	-4.53779	0.01258
H	-6.10131	-2.42841	0.16859
C	-4.63951	-5.49635	-0.15172
H	-2.51649	-5.84038	-0.39767

H	-6.68844	-4.84775	0.10304
H	-4.89681	-6.55809	-0.18592
C	4.44291	-2.37609	0.10073
C	5.82772	-2.49070	0.27765
C	3.65479	-3.52640	-0.05464
C	6.42384	-3.75415	0.29625
H	6.43719	-1.59139	0.38885
C	4.25694	-4.78618	-0.03889
H	2.57342	-3.42332	-0.17120
C	5.64042	-4.90179	0.13569
H	7.50425	-3.84216	0.43300
H	3.64227	-5.68131	-0.15966
H	6.10910	-5.88884	0.14803

Calculated cartesian coordinates and energies of C

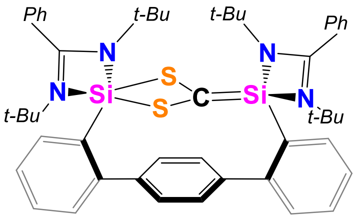

C

E(B3LYP)	-3494.878431 E_h
Sum of electronic and zero-point Energies	-3493.916430 E_h
Sum of electronic and thermal Energies	-3493.859813 E_h
Sum of electronic and thermal Enthalpies	-3493.858869 E_h
Sum of electronic and thermal Free Energies	-3494.005707 E_h

Number of imaginary frequencies: 0.

S	-0.52084	-0.32524	0.79504
S	-0.52465	1.56521	-1.35669
Si	-2.23807	0.74791	-0.10430
Si	2.24946	0.91995	0.00413
N	-3.13040	-0.47512	-1.20445
N	-3.39631	-0.54519	0.94029
N	3.38781	-0.27974	-0.96939

N	2.97755	-0.35870	1.14972
C	0.54549	0.67039	-0.24142
C	3.40849	-0.65339	-2.39968
C	0.54194	3.24674	1.49826
C	-0.84656	3.19446	1.56653
C	-3.64451	-1.18986	-0.17948
C	3.20812	2.55327	-0.03379
C	3.58564	-0.99953	0.12990
C	-3.06318	3.57030	0.45639
C	2.63940	3.83114	0.23916
C	-1.62922	3.90503	0.64112
C	-3.44882	2.23698	0.15635
C	-3.74064	-0.84159	2.34094
C	-3.12322	-0.72487	-2.66609
C	0.39783	4.82941	-0.31491
C	-0.99687	4.80649	-0.22581
C	-4.82039	1.99705	-0.06981
C	1.18117	3.99443	0.49442
C	3.06299	-0.58758	2.60943
C	4.59698	2.48650	-0.27435
C	-5.37999	4.31950	0.28874
C	-4.03153	4.58993	0.51296
C	-5.77675	3.01185	-0.00777
C	3.46476	4.96879	0.22709
C	5.40974	3.62231	-0.25565
C	4.83719	4.87259	-0.01236
H	-5.15465	0.98852	-0.32016
H	-6.82971	2.78274	-0.19427
H	-6.11861	5.12367	0.34536
H	-3.70663	5.60697	0.74828
H	5.04993	1.51903	-0.50399
H	6.48323	3.53193	-0.44275
H	5.45758	5.77287	-0.00314
H	3.01344	5.94354	0.42916

H	1.13577	2.68348	2.21243
H	-1.33003	2.55888	2.30955
H	0.88135	5.43680	-1.08466
H	-1.59687	5.37869	-0.93752
C	-4.31470	-1.58225	-3.13185
H	-5.26790	-1.17938	-2.75678
H	-4.34866	-1.56347	-4.23182
H	-4.23551	-2.63170	-2.82379
C	-1.80248	-1.41221	-3.06172
H	-0.95000	-0.78719	-2.76200
H	-1.71598	-2.39202	-2.56680
H	-1.76324	-1.57118	-4.15175
C	-3.24389	0.64577	-3.36286
H	-3.27144	0.50887	-4.45510
H	-4.16677	1.15725	-3.04932
H	-2.38749	1.29086	-3.13025
C	-5.27026	-0.85452	2.53051
H	-5.73481	-1.70087	2.00714
H	-5.51849	-0.94651	3.59982
H	-5.71009	0.08144	2.15284
C	-3.12991	-2.17847	2.80322
H	-3.29489	-2.31421	3.88406
H	-3.58319	-3.03361	2.28320
H	-2.04617	-2.18777	2.61150
C	-3.15254	0.29997	3.19172
H	-2.05765	0.33016	3.09758
H	-3.55519	1.27215	2.87100
H	-3.40945	0.15001	4.25151
C	2.29037	0.55482	3.28473
H	1.24288	0.56487	2.94781
H	2.30302	0.41905	4.37669
H	2.75358	1.52772	3.05994
C	2.39993	-1.92400	2.99291
H	2.34757	-2.01366	4.08940

H	1.37682	-1.96500	2.58974
H	2.96815	-2.78450	2.61540
C	4.52935	-0.54933	3.07674
H	4.57727	-0.61307	4.17517
H	5.10845	-1.38902	2.66824
H	5.00709	0.39236	2.76362
C	2.32884	-1.71855	-2.67402
H	2.24065	-1.90450	-3.75627
H	2.58170	-2.67228	-2.18810
H	1.35791	-1.37142	-2.28947
C	3.06392	0.62880	-3.17904
H	2.05505	0.98754	-2.92093
H	3.78587	1.42931	-2.95826
H	3.08422	0.42767	-4.26080
C	4.79558	-1.15496	-2.83348
H	5.57365	-0.41862	-2.57729
H	5.05384	-2.11305	-2.36314
H	4.80964	-1.30273	-3.92468
C	-4.24418	-2.54973	-0.29609
C	-3.36386	-3.64043	-0.36201
C	-5.62627	-2.76696	-0.34557
C	-3.86600	-4.93800	-0.47985
H	-2.28801	-3.45609	-0.31196
C	-6.12568	-4.06630	-0.46609
H	-6.31034	-1.91677	-0.30838
C	-5.24767	-5.15294	-0.53458
H	-3.17695	-5.78477	-0.52987
H	-7.20512	-4.23035	-0.51072
H	-5.64051	-6.16826	-0.63021
C	4.28949	-2.30810	0.20654
C	5.69047	-2.35882	0.25277
C	3.55090	-3.50098	0.20061
C	6.34580	-3.59119	0.29912
H	6.26311	-1.42888	0.24977

C	4.21034	-4.73195	0.23671
H	2.46042	-3.45656	0.17004
C	5.60729	-4.77936	0.28823
H	7.43723	-3.62397	0.33881
H	3.62997	-5.65773	0.22799
H	6.12128	-5.74318	0.31967

Calculated cartesian coordinates and energies of TS(C-D)

E(B3LYP)	-3494.853950 E_h
Sum of electronic and zero-point Energies	-3493.892996 E_h
Sum of electronic and thermal Energies	-3493.837189 E_h
Sum of electronic and thermal Enthalpies	-3493.836245 E_h
Sum of electronic and thermal Free Energies	-3493.981237 E_h

Number of imaginary frequencies: 1, ν = -279.0 cm^{-1}.

S	-0.37054	-0.44616	0.68920
S	-0.28509	1.32490	-1.59964
Si	-2.21649	0.87199	-0.16098
Si	2.31681	0.95582	0.15199
N	-2.97695	-0.63172	-1.10523
N	-3.34480	-0.28993	1.00780
N	3.44329	-0.05652	-0.92316
N	2.99645	-0.43010	1.15485
C	0.56642	0.77570	-0.16291
C	3.58917	-0.17086	-2.39335
C	0.07091	2.61911	1.18582
C	-1.36728	2.61169	0.95462
C	-3.54626	-1.12806	-0.00510
C	3.10908	2.56959	0.60195
C	3.60441	-0.93986	0.07185
C	-3.31062	3.47347	-0.31443
C	2.33023	3.72699	0.85488
C	-1.93416	3.69657	0.14812
C	-3.63448	2.12112	-0.56819

C	-3.77250	-0.41343	2.41567
C	-2.88690	-1.17598	-2.47869
C	0.26082	4.69824	-0.08811
C	-1.14036	4.69892	-0.34629
C	-4.91977	1.82753	-1.05187
C	0.85938	3.70682	0.66729
C	3.04553	-0.81915	2.58344
C	4.51025	2.63102	0.72508
C	-5.51623	4.17761	-1.03623
C	-4.24611	4.49603	-0.55927
C	-5.85697	2.83848	-1.28061
C	2.99388	4.91138	1.23055
C	5.15134	3.81143	1.10227
C	4.38283	4.95423	1.35475
H	-5.19383	0.78955	-1.26069
H	-6.85508	2.58839	-1.65207
H	-6.24933	4.96996	-1.21175
H	-3.97734	5.53504	-0.34917
H	5.10982	1.74137	0.50843
H	6.23963	3.84375	1.19809
H	4.87091	5.88501	1.65653
H	2.39808	5.80271	1.44013
H	0.43481	2.11587	2.08268
H	-1.97611	2.36388	1.82530
H	0.88322	5.47497	-0.54062
H	-1.55930	5.44432	-1.02675
C	-4.08948	-2.05863	-2.87169
H	-5.04286	-1.54843	-2.66624
H	-4.03848	-2.25580	-3.95359
H	-4.09858	-3.02920	-2.36253
C	-1.58571	-1.99111	-2.61180
H	-0.72878	-1.37919	-2.30239
H	-1.62403	-2.88350	-1.96821
H	-1.44088	-2.32163	-3.65354

C	-2.87174	0.01460	-3.45950
H	-2.80645	-0.35913	-4.49326
H	-3.78959	0.61206	-3.36111
H	-2.01069	0.66876	-3.28504
C	-5.30963	-0.35689	2.52486
H	-5.77851	-1.25479	2.10276
H	-5.61557	-0.28810	3.58129
H	-5.69375	0.52587	1.99078
C	-3.22828	-1.70165	3.06799
H	-3.43425	-1.68995	4.15036
H	-3.69193	-2.60538	2.65273
H	-2.13883	-1.76540	2.92121
C	-3.19694	0.78144	3.19091
H	-2.09883	0.78655	3.13563
H	-3.58445	1.73071	2.79370
H	-3.48913	0.71422	4.24973
C	2.21772	0.22597	3.34764
H	1.17271	0.20588	3.00333
H	2.23450	0.00068	4.42425
H	2.62735	1.23809	3.20541
C	2.42412	-2.20975	2.79948
H	2.35610	-2.41922	3.87839
H	1.41129	-2.23571	2.37119
H	3.02959	-3.00400	2.34197
C	4.50296	-0.78321	3.07642
H	4.54041	-0.99062	4.15707
H	5.11834	-1.53983	2.56859
H	4.94494	0.20991	2.89961
C	2.48395	-1.07917	-2.96214
H	2.55632	-1.11764	-4.06047
H	2.58203	-2.10622	-2.58025
H	1.49044	-0.69090	-2.69301
C	3.41988	1.25858	-2.93761
H	2.42433	1.66314	-2.69190

H	4.18557	1.93020	-2.51968
H	3.51546	1.25757	-4.03361
C	4.98092	-0.69969	-2.77481
H	5.77229	-0.10511	-2.29177
H	5.11525	-1.75330	-2.49679
H	5.11164	-0.62250	-3.86501
C	-4.20425	-2.46619	0.08319
C	-3.39250	-3.59923	0.24269
C	-5.59228	-2.61918	-0.02095
C	-3.96581	-4.87138	0.29841
H	-2.31130	-3.46876	0.32718
C	-6.16422	-3.89287	0.03141
H	-6.22242	-1.73908	-0.16371
C	-5.35296	-5.02093	0.19120
H	-3.32804	-5.74989	0.42489
H	-7.24766	-4.00510	-0.05714
H	-5.80156	-6.01675	0.23120
C	4.23233	-2.28239	-0.03349
C	5.61956	-2.44968	0.06839
C	3.40465	-3.39228	-0.26084
C	6.17752	-3.72446	-0.05470
H	6.25956	-1.58112	0.23693
C	3.96883	-4.66314	-0.39173
H	2.32350	-3.25093	-0.32832
C	5.35385	-4.83081	-0.28758
H	7.25932	-3.85323	0.02726
H	3.32336	-5.52583	-0.57267
H	5.79253	-5.82658	-0.38775

Calculated cartesian coordinates and energies of D

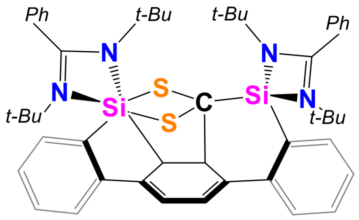

D

E(B3LYP)	-3494.882471 E_h
Sum of electronic and zero-point Energies	-3493.918920 E_h
Sum of electronic and thermal Energies	-3493.863538 E_h
Sum of electronic and thermal Enthalpies	-3493.862593 E_h
Sum of electronic and thermal Free Energies	-3494.005775 E_h

Number of imaginary frequencies: 0.

S	-0.30419	-0.24790	1.03354
S	-0.00869	1.22372	-1.49879
Si	-2.05274	0.98707	-0.20496
Si	2.45879	0.90202	0.36394
N	-2.70975	-0.67634	-1.00266
N	-3.35369	0.04518	0.94120
N	3.22196	-0.19336	-0.88420
N	3.04151	-0.60974	1.22565
C	0.62967	1.12866	0.23668
C	3.52080	-0.16156	-2.33864
C	0.21050	2.46942	0.95086
C	-1.29877	2.63441	0.67966
C	-3.45659	-0.95201	0.06317
C	3.29518	2.46337	0.85461
C	3.34503	-1.15963	0.04140
C	-2.98827	3.50978	-0.91329
C	2.51652	3.65823	0.89661
C	-1.68451	3.72783	-0.26742
C	-3.33546	2.14632	-1.06099
C	-3.97403	0.16447	2.27716
C	-2.43381	-1.45660	-2.22765

C	0.53796	4.70903	-0.13298
C	-0.82481	4.72501	-0.60410
C	-4.54149	1.83358	-1.70269
C	1.07718	3.66456	0.55610
C	3.04479	-1.15065	2.60692
C	4.67066	2.49779	1.15472
C	-5.02767	4.18732	-2.03391
C	-3.82951	4.52450	-1.40417
C	-5.38655	2.83950	-2.18338
C	3.18297	4.85133	1.26191
C	5.30417	3.68934	1.49821
C	4.54507	4.86789	1.54951
H	-4.82802	0.78500	-1.82917
H	-6.32812	2.57825	-2.67550
H	-5.69140	4.97428	-2.40299
H	-3.55458	5.57422	-1.26832
H	5.25385	1.57188	1.11269
H	6.37123	3.70560	1.73259
H	5.02227	5.80969	1.83444
H	2.60590	5.77450	1.34501
H	0.36481	2.30093	2.03144
H	-1.84865	2.79844	1.61290
H	1.18616	5.53682	-0.43660
H	-1.11364	5.48408	-1.33556
C	-3.56935	-2.42152	-2.62917
H	-4.53844	-1.90198	-2.67138
H	-3.35265	-2.81237	-3.63549
H	-3.66724	-3.28279	-1.95805
C	-1.13153	-2.25813	-2.02348
H	-0.32913	-1.58209	-1.70178
H	-1.27233	-3.02352	-1.24363
H	-0.83630	-2.76365	-2.95798
C	-2.27046	-0.46495	-3.39876
H	-2.04814	-1.01881	-4.32438

H	-3.19443	0.11144	-3.55047
H	-1.45106	0.24025	-3.22420
C	-5.50996	0.24991	2.16454
H	-5.94839	-0.70107	1.83626
H	-5.95044	0.49599	3.14427
H	-5.79336	1.03550	1.44707
C	-3.55962	-1.00217	3.19878
H	-3.90140	-0.80773	4.22816
H	-3.99605	-1.95745	2.88084
H	-2.46280	-1.09868	3.20632
C	-3.47355	1.46451	2.92276
H	-2.38178	1.43875	3.04327
H	-3.74888	2.33887	2.31588
H	-3.92955	1.58253	3.91747
C	2.41980	-0.06378	3.49755
H	1.36993	0.10780	3.21268
H	2.44760	-0.38506	4.54938
H	2.97696	0.88293	3.41681
C	2.22161	-2.44440	2.72207
H	2.12567	-2.71883	3.78393
H	1.21682	-2.28684	2.30505
H	2.70084	-3.28450	2.20134
C	4.50411	-1.39207	3.03321
H	4.53998	-1.72793	4.08107
H	4.97682	-2.16911	2.41488
H	5.09238	-0.46496	2.94722
C	2.50155	-0.99557	-3.13105
H	2.68464	-0.87451	-4.21013
H	2.58672	-2.06503	-2.89171
H	1.48175	-0.65523	-2.90363
C	3.43341	1.31602	-2.75693
H	2.41914	1.71393	-2.59754
H	4.15128	1.92828	-2.18842
H	3.67057	1.41264	-3.82688

C	4.95195	-0.67161	-2.58232
H	5.67663	-0.11065	-1.97068
H	5.05044	-1.74173	-2.35442
H	5.21594	-0.53101	-3.64156
C	-4.20708	-2.22698	0.26878
C	-3.51087	-3.34912	0.74196
C	-5.56752	-2.33968	-0.04326
C	-4.17030	-4.56964	0.90209
H	-2.45027	-3.25293	0.98462
C	-6.22581	-3.56189	0.11448
H	-6.10440	-1.47029	-0.42805
C	-5.52923	-4.67887	0.58671
H	-3.62208	-5.43931	1.27323
H	-7.28618	-3.64353	-0.13703
H	-6.04511	-5.63467	0.70857
C	3.57993	-2.60137	-0.22199
C	4.84103	-3.20090	-0.12612
C	2.45116	-3.36683	-0.55768
C	4.97478	-4.57102	-0.36940
H	5.71536	-2.59789	0.12698
C	2.59398	-4.73391	-0.79808
H	1.47157	-2.88498	-0.60996
C	3.85367	-5.33671	-0.70449
H	5.95877	-5.04030	-0.29830
H	1.71608	-5.32997	-1.05784
H	3.96208	-6.40746	-0.89374

Calculated cartesian coordinates and energies of TS(D-E)

E(B3LYP)	-3494.877599 E_h
Sum of electronic and zero-point Energies	-3493.914041 E_h
Sum of electronic and thermal Energies	-3493.859314 E_h
Sum of electronic and thermal Enthalpies	-3493.858370 E_h
Sum of electronic and thermal Free Energies	-3494.000593 E_h

Number of imaginary frequencies: 1, v = -90.8 cm^{-1}.

S	-0.38517	-0.29437	0.68962
S	0.29848	1.50207	-1.66072
Si	-2.25829	0.93988	-0.04677
Si	2.40115	0.94881	0.20301
N	-2.88328	-0.62253	-1.04649
N	-3.41410	-0.13524	1.00144
N	3.26055	-0.22566	-0.90292
N	3.01381	-0.48076	1.22696
C	0.58474	1.15514	0.12537
C	3.63724	-0.30566	-2.34060
C	0.12411	2.40288	0.95904
C	-1.39325	2.55352	0.73908
C	-3.55105	-1.04234	0.01473
C	3.21744	2.52956	0.68156
C	3.42141	-1.09370	0.11423
C	-3.15996	3.48637	-0.73039
C	2.39779	3.67163	0.91629
C	-1.83556	3.69231	-0.12964
C	-3.54616	2.13183	-0.84675
C	-3.83914	-0.21794	2.41488
C	-2.66586	-1.20922	-2.38572
C	0.35276	4.73890	0.06848
C	-1.01745	4.74295	-0.38652
C	-4.79011	1.83859	-1.42573
C	0.94089	3.65855	0.64968
C	2.93662	-0.92232	2.63772
C	4.60674	2.59973	0.89107
C	-5.23838	4.19750	-1.75745
C	-4.00599	4.51408	-1.18990
C	-5.63579	2.85621	-1.87434
C	3.03474	4.84401	1.38199
C	5.21347	3.77423	1.33257
C	4.41279	4.89741	1.58049
H	-5.10487	0.79641	-1.53472

H	-6.60594	2.61135	-2.31638
H	-5.90048	4.99566	-2.10466
H	-3.70514	5.55928	-1.07976
H	5.22374	1.71503	0.70190
H	6.29379	3.81652	1.49164
H	4.86861	5.82207	1.94526
H	2.42468	5.72065	1.60976
H	0.29768	2.15851	2.02215
H	-1.90116	2.68426	1.70653
H	0.96771	5.60855	-0.18037
H	-1.34147	5.55355	-1.04412
C	-3.82519	-2.10652	-2.85843
H	-4.79055	-1.58375	-2.77702
H	-3.66436	-2.35430	-3.91896
H	-3.89412	-3.05055	-2.30421
C	-1.35172	-2.01477	-2.35976
H	-0.52477	-1.37201	-2.02808
H	-1.43445	-2.86448	-1.66341
H	-1.12842	-2.40915	-3.36452
C	-2.53607	-0.04408	-3.38498
H	-2.28386	-0.43944	-4.38135
H	-3.47810	0.51733	-3.46055
H	-1.74179	0.64709	-3.07619
C	-5.37324	-0.31146	2.52749
H	-5.75443	-1.27028	2.15427
H	-5.67956	-0.22111	3.58182
H	-5.84661	0.50205	1.95606
C	-3.16547	-1.40968	3.12496
H	-3.38464	-1.38098	4.20444
H	-3.52676	-2.37150	2.73730
H	-2.07476	-1.35842	2.98405
C	-3.38498	1.07351	3.11118
H	-2.28898	1.15557	3.10039
H	-3.81633	1.95848	2.61981

H	-3.71730	1.06713	4.16017
C	2.15211	0.16574	3.39023
H	1.11956	0.22090	3.01481
H	2.12013	-0.07424	4.46343
H	2.63069	1.15083	3.27547
C	2.19808	-2.26482	2.77529
H	2.03755	-2.48496	3.84213
H	1.21872	-2.20772	2.27770
H	2.77170	-3.09677	2.34452
C	4.36103	-1.01667	3.21399
H	4.31873	-1.24600	4.29010
H	4.93894	-1.81352	2.72476
H	4.89387	-0.06167	3.08405
C	2.53915	-1.03456	-3.13196
H	2.78629	-1.03019	-4.20541
H	2.45210	-2.08232	-2.80762
H	1.57492	-0.52845	-2.97993
C	3.77722	1.14560	-2.83311
H	2.83079	1.69309	-2.71164
H	4.56796	1.67282	-2.27590
H	4.04525	1.14863	-3.90052
C	4.99025	-1.01770	-2.51530
H	5.75757	-0.58043	-1.85630
H	4.92921	-2.09532	-2.31664
H	5.32454	-0.89198	-3.55607
C	-4.27804	-2.34181	0.11520
C	-3.55098	-3.51191	0.37736
C	-5.65981	-2.41822	-0.10220
C	-4.20278	-4.74644	0.42589
H	-2.47311	-3.44560	0.54043
C	-6.30889	-3.65421	-0.05967
H	-6.22094	-1.50643	-0.31696
C	-5.58215	-4.81987	0.20491
H	-3.63149	-5.65484	0.63270

H	-7.38586	-3.70815	-0.23669
H	-6.09128	-5.78637	0.23755
C	3.83706	-2.51357	-0.00974
C	5.13659	-2.94194	0.28773
C	2.86692	-3.43622	-0.43082
C	5.46757	-4.29299	0.15922
H	5.89150	-2.21721	0.59837
C	3.20352	-4.78584	-0.55184
H	1.85211	-3.09002	-0.64081
C	4.50266	-5.21517	-0.25924
H	6.48354	-4.62589	0.38412
H	2.44721	-5.50475	-0.87550
H	4.76415	-6.27157	-0.35766

Calculated cartesian coordinates and energies of E

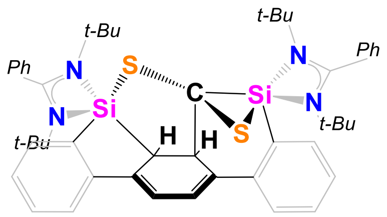

E

E(B3LYP)	-3494.907661 E_h
Sum of electronic and zero-point Energies	-3493.944193 E_h
Sum of electronic and thermal Energies	-3493.888609 E_h
Sum of electronic and thermal Enthalpies	-3493.887665 E_h
Sum of electronic and thermal Free Energies	-3494.032396 E_h

Number of imaginary frequencies: 0.

S	0.59546	0.21227	-0.79352
S	-1.42487	-2.00399	-1.62096
Si	2.50206	-0.76330	-0.26746
Si	-2.39706	-0.85393	-0.04962
N	3.39194	0.95134	-1.07323
N	3.41428	0.26564	0.99279
N	-4.02457	-0.20573	-0.83424

N	-2.75702	0.96324	0.46163
C	-0.54377	-1.06636	-0.20566
C	-4.75577	2.17332	-0.48572
C	-4.43637	3.09578	-1.49156
C	-4.92213	-0.69015	-1.90527
C	0.10578	-1.92754	0.90666
C	1.58480	-2.21312	0.65518
C	3.72364	1.28667	0.15182
C	-2.90835	-1.96999	1.38054
C	4.16695	2.63854	0.60394
C	-3.86878	0.99550	-0.27728
C	-5.27353	4.18993	-1.72883
C	-5.91376	2.35572	0.28322
C	-6.42794	4.36941	-0.96110
C	3.01922	-3.34790	-1.03732
C	-2.03789	-3.01605	1.80195
C	1.82574	-3.44273	-0.18456
C	3.53544	-2.03572	-1.21437
C	3.19212	3.48217	1.16009
C	3.81168	-0.00551	2.39806
C	-6.74462	3.45155	0.04704
C	3.37236	1.75137	-2.31972
C	-0.25203	-4.37407	0.70727
C	0.95446	-4.48073	-0.09475
C	2.46221	2.99042	-2.19714
C	4.70091	-1.85706	-1.97219
C	-0.70617	-3.18068	1.17468
C	2.82266	0.84091	-3.43540
C	5.48795	3.08689	0.49111
C	4.79824	2.18943	-2.71055
C	2.54293	-0.04935	3.27154
C	-2.22196	1.93680	1.43905
C	-4.16377	-1.84728	1.99669
C	4.81501	-4.23688	-2.40843

C	4.55804	-1.35769	2.42563
C	3.66399	-4.44003	-1.64974
C	3.53850	4.76302	1.59392
C	-4.32189	-0.30808	-3.27247
C	5.34415	-2.94655	-2.56502
C	-3.31479	2.42157	2.41133
C	-2.46873	-3.89701	2.81156
C	4.86108	5.20697	1.48359
C	4.76843	1.03963	2.99950
C	-1.13732	1.21793	2.26152
C	-1.58584	3.12767	0.69809
C	-5.00472	-2.22182	-1.75991
C	5.83400	4.36667	0.93346
C	-6.35062	-0.12745	-1.78765
C	-4.57505	-2.72164	3.00684
C	-3.72264	-3.75508	3.40859
H	5.11193	-0.85248	-2.09490
H	6.25564	-2.79685	-3.15013
H	5.31551	-5.09051	-2.87403
H	3.27477	-5.45175	-1.51130
H	6.86874	4.70781	0.84953
H	2.77412	5.41685	2.02117
H	-4.84594	-1.05804	1.66542
H	-5.55647	-2.60245	3.47390
H	-4.03164	-4.44805	4.19600
H	-1.79320	-4.69068	3.14100
H	-7.64551	3.58860	0.64993
H	-5.02154	4.90363	-2.51687
H	0.04922	-1.29586	1.81029
H	2.07118	-2.40456	1.62690
H	-0.87607	-5.26493	0.82328
H	1.10202	-5.39332	-0.67906
H	1.45206	2.69683	-1.87671
H	2.85960	3.72166	-1.48035

H	2.38158	3.48967	-3.17580
H	3.44566	-0.05531	-3.55847
H	1.79832	0.51317	-3.21307
H	2.81413	1.38965	-4.38982
H	5.20287	2.93448	-2.01376
H	5.48022	1.32536	-2.73728
H	4.78746	2.64334	-3.71395
H	2.02483	0.92119	3.23375
H	1.83828	-0.81826	2.93254
H	2.80274	-0.26576	4.31974
H	3.95390	-2.17932	2.02287
H	5.47536	-1.29445	1.82034
H	4.83889	-1.61903	3.45802
H	-4.29645	0.78753	-3.38677
H	-3.29499	-0.69010	-3.36270
H	-4.93201	-0.72178	-4.09141
H	-4.05572	3.06503	1.92065
H	-3.83497	1.56184	2.86276
H	-2.85527	3.00740	3.22260
H	5.68614	1.14474	2.40472
H	4.30919	2.02959	3.10659
H	5.05901	0.69610	4.00390
H	-1.54600	0.32535	2.75909
H	-0.29399	0.91803	1.62693
H	-0.75474	1.89806	3.03812
H	-0.81728	2.76701	-0.00216
H	-2.34056	3.69727	0.13637
H	-1.11042	3.81418	1.41750
H	-4.01830	-2.69514	-1.84712
H	-5.42421	-2.49283	-0.77891
H	-5.65593	-2.63526	-2.54502
H	-6.75421	-0.28123	-0.77516
H	-6.40781	0.94186	-2.02665
H	-7.00111	-0.66064	-2.49788

H	-3.53409	2.95055	-2.08954
H	-6.16100	1.63513	1.06510
H	-7.08173	5.22499	-1.14711
H	2.16209	3.12622	1.24122
H	6.24939	2.42612	0.07186
H	5.13325	6.20813	1.82690

5.2 Compound 6 – NBO

Natural population analysis on heterocycle **6** at the B3LYP-D3(BJ) / def2-TZVP // B3LYP-D3(BJ) / def2-SVP level using NBO 6[11] in Gaussian 16 A.03.[12] Natural populations in the mesoionic heterocycles Münchnones and Montrealones are given for reference, as well as the pyrido-annealed aza-analogues for as close a comparison as possible.

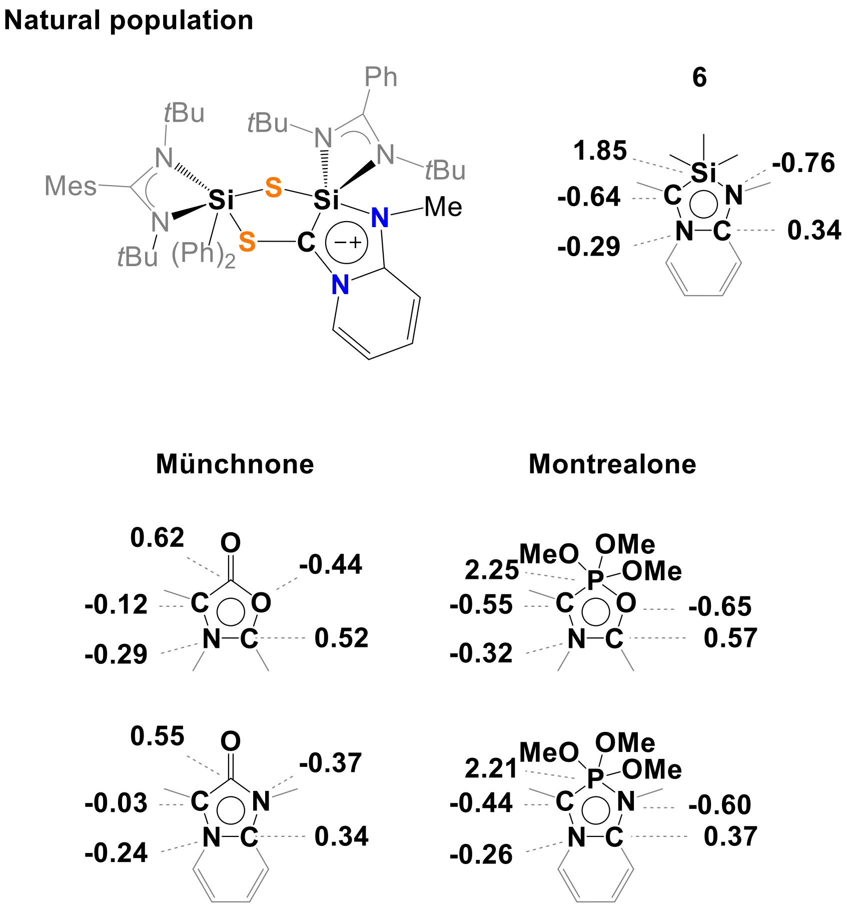

Figure S46. NBO Analysis of compound **6** and five membered heteroatom containing compounds.

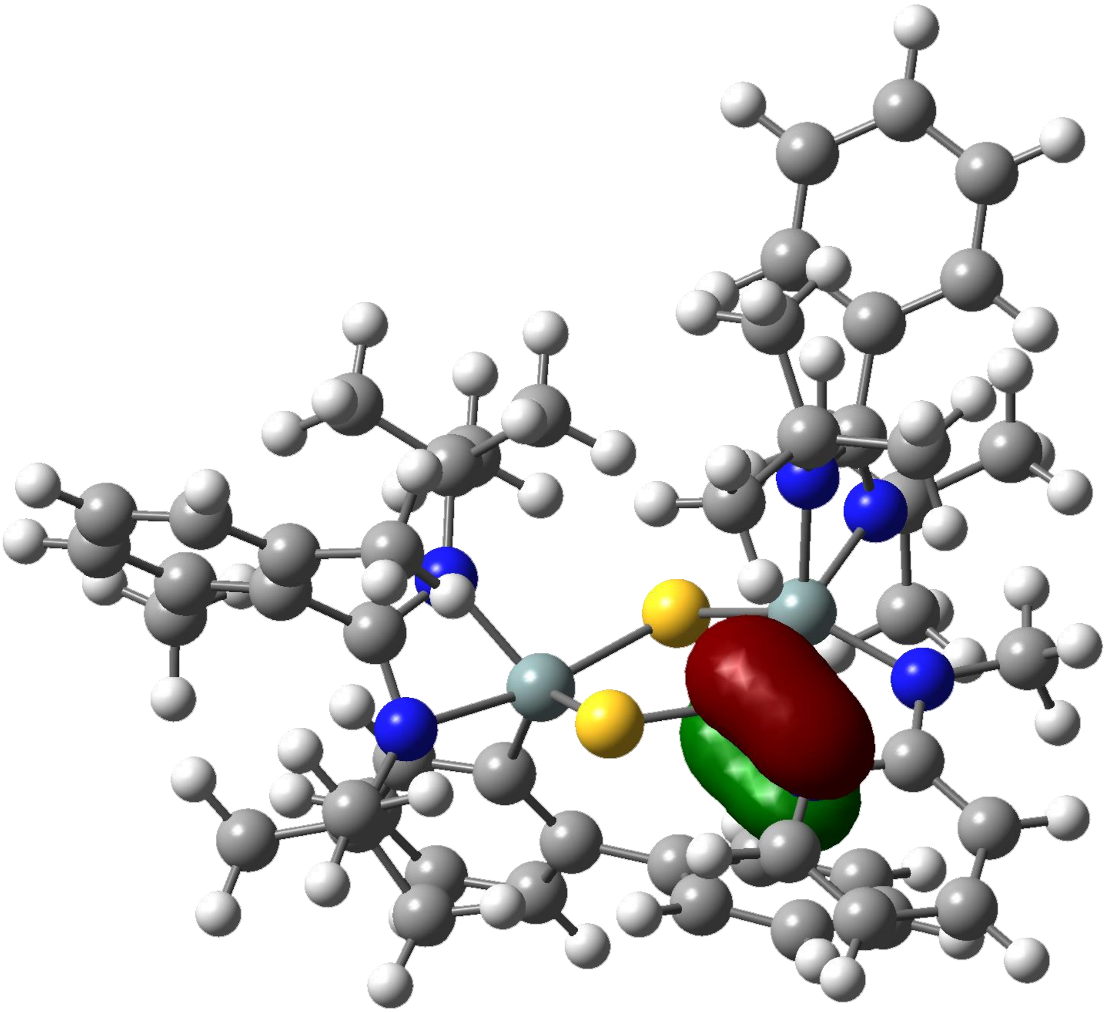

Figure S47. NB-Orbital 212: π(C−N) of compound **6**.

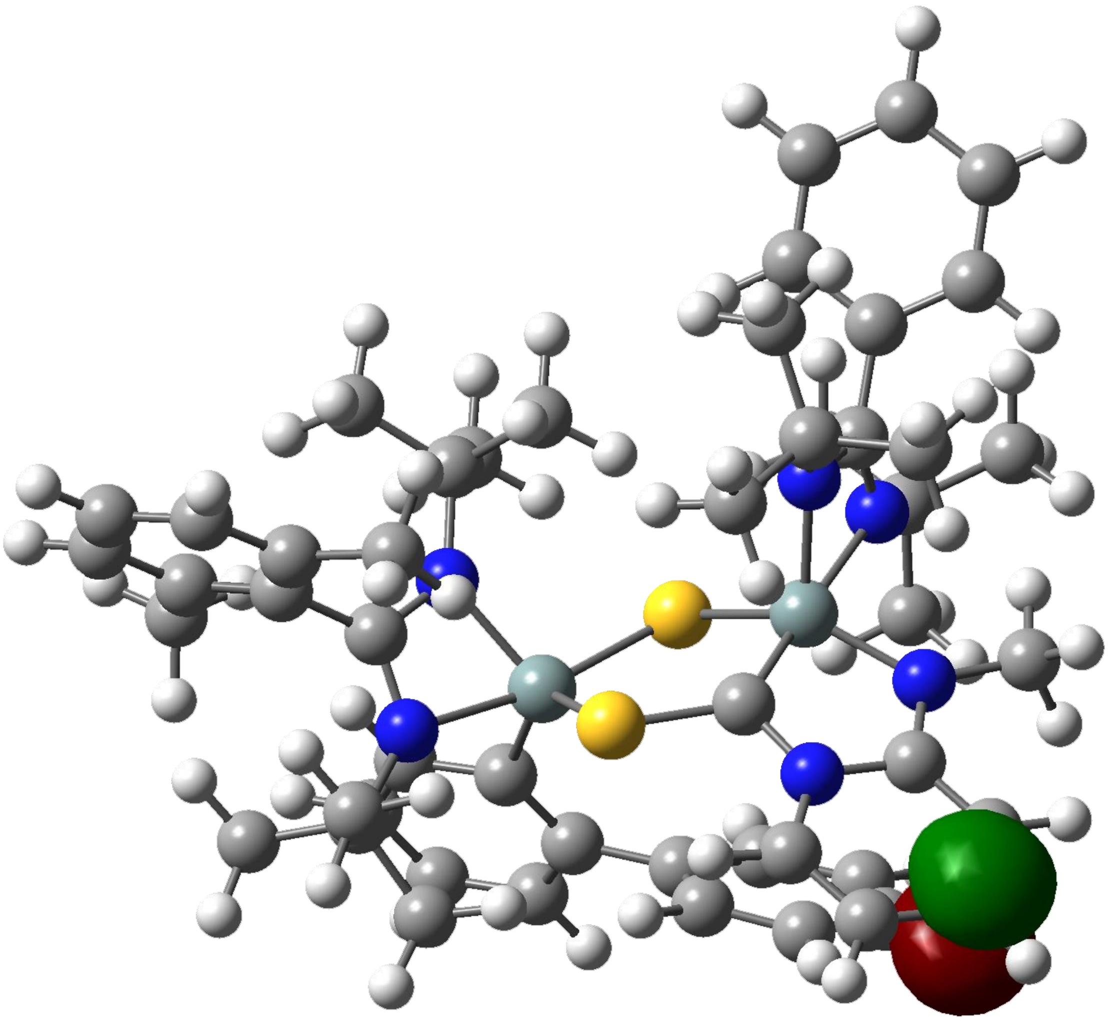

Figure 48. NB-HOMO (237): component of pyridine π-system, anion, of compound **6**.

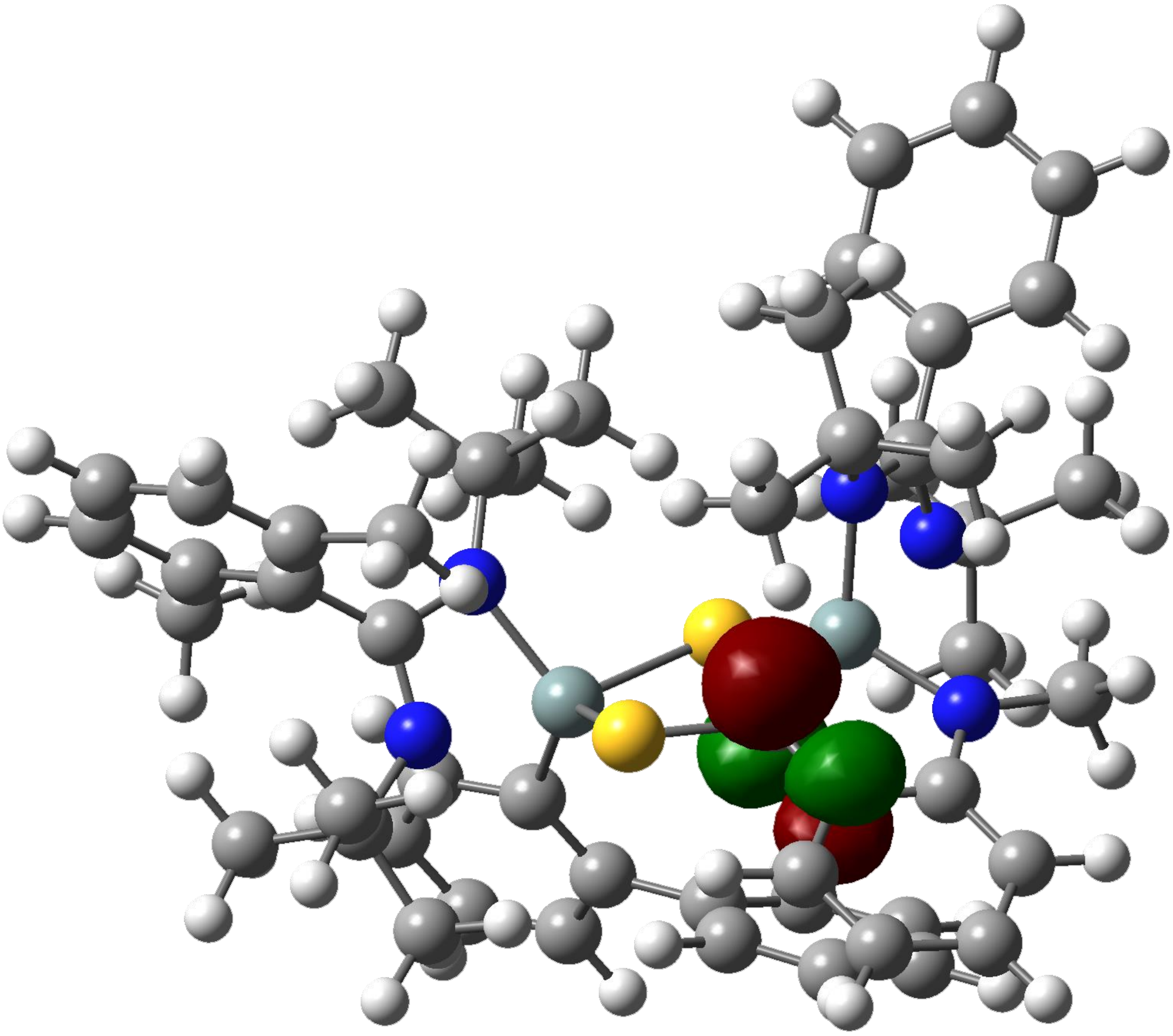

Figure S49. NB-LUMO (238): π*(C−N) of compound **6**.

Electronic energy of **6** at B3LYP-D3(BJ) / def2-SVP:	3724.8544727 Hartree
Electronic energy of **6** at B3LYP-D3(BJ) / def2-TZVP:	-3727.8609604 Hartree

Number of imaginary frequencies: 0

xyz coordinates for **6**

S	0.78149400	-0.32375300	-1.29905700
S	-0.75493200	-1.02141100	1.59521500
Si	-1.24583900	-0.78997600	-0.53680200
Si	2.13828100	-0.24927100	0.43905800
N	3.47549300	0.63026800	-0.81724800
N	-3.19469700	-0.82596800	0.22981100
N	1.61643300	-1.90938200	2.49379500

N	2.41357700	1.56924900	0.82917600
N	-2.19098900	0.81132700	-0.77259900
N	3.44472100	-1.22698800	1.26591500
C	-3.31490700	0.43590100	-0.10124200
C	3.84849900	3.09391700	-0.56036900
C	-1.67566700	-2.07407200	-1.87897200
C	3.27928100	1.77068500	-0.18600000
C	-1.84191300	2.09569000	-1.43722400
C	-4.43980500	1.33477200	0.30236700
C	-5.60709600	1.45656800	-0.47470300
C	3.07074400	3.99443100	-1.30068100
C	0.31658900	-3.63322800	-1.47417800
C	0.99344500	-1.04473900	1.63866800
C	5.13986400	3.45870800	-0.15984300
C	-4.30717300	2.04950000	1.51316200
C	2.26348000	2.36026900	2.07461100
C	1.02313900	-2.65744500	3.48871100
C	4.07629800	0.38647000	-2.15033400
C	2.99895300	-2.03204700	2.28173200
C	-0.89928800	-3.20853900	-2.22054100
C	-6.64068900	2.28102300	-0.01516300
C	0.28418900	-3.90494000	-0.09908800
C	3.58192400	5.24851700	-1.63552800
C	-2.79236000	-1.77853700	-2.68212800
C	-3.04671400	1.99802800	2.34124600
C	5.64571100	4.71852400	-0.48764900
C	-5.36630400	2.86142200	1.93165800
C	-4.10645100	-1.72879900	0.95691100
C	-6.54502600	2.98826600	1.18711700
C	1.52471800	-3.82757100	-2.16579000
C	5.56013300	0.79818000	-2.18345400
C	-3.00454300	3.09641300	-1.52448100
C	3.97407100	-1.12400700	-2.40831800
C	4.86802500	5.61492500	-1.22615100

C	3.31508300	1.13570400	-3.25927000
C	3.73956500	-2.91631700	3.05838900
C	-0.69875300	2.77197600	-0.66179000
C	-5.76186300	0.77441500	-1.81087100
C	1.75538700	-3.54245000	4.26191800
C	2.67346500	-4.24852600	-1.49674300
C	2.13440700	3.87152400	1.81271100
C	-3.46047700	-3.12444300	0.93456700
C	3.12974100	-3.68800200	4.05959500
C	4.87331900	-1.24307300	1.03082700
C	2.63024900	-4.50490200	-0.12322300
C	-1.27510300	-3.99182100	-3.32890800
C	1.42833400	-4.34589600	0.56832700
C	0.98273200	1.90329800	2.78719600
C	3.47836900	2.08145000	2.97830900
C	-3.15287800	-2.55543500	-3.78215300
C	-2.38835800	-3.67786100	-4.10584000
C	-5.47610200	-1.83086000	0.26395500
C	-1.41353000	1.77691900	-2.88210300
C	-4.29187500	-1.28217100	2.41780900
C	-7.68510800	3.83977500	1.68393300
H	-0.04703100	-2.50397100	3.60381500
H	1.23114700	-4.11015400	5.03277800
H	3.72249100	-4.37426300	4.66546900
H	4.80654300	-3.01247900	2.86640600
H	5.22438800	-2.23595100	0.69514000
H	5.12332700	-0.50862800	0.26427400
H	5.42955800	-0.98268700	1.94901800
H	4.40891500	2.42654500	2.50165900
H	3.37691300	2.60356600	3.94287200
H	3.56911400	1.00257900	3.17429600
H	0.81011600	2.53633700	3.67040500
H	0.11382600	1.98159100	2.12084200
H	1.05255000	0.85816700	3.10918800

H	3.06569300	4.32959800	1.46129400
H	1.34570500	4.07419000	1.07436100
H	1.85622000	4.36849800	2.75445300
H	5.74600300	2.75408000	0.41266700
H	6.65125700	4.99994600	-0.16685200
H	5.26468600	6.59965200	-1.48380400
H	2.97177300	5.94591100	-2.21402500
H	2.06214700	3.70685300	-1.60081000
H	3.70845400	0.83913800	-4.24414700
H	2.24542000	0.89052500	-3.21956500
H	3.43598600	2.22328200	-3.16566100
H	4.51920300	-1.69890300	-1.64772600
H	2.92773500	-1.45121200	-2.39226000
H	4.40324100	-1.36490200	-3.39255700
H	6.12713000	0.32463000	-1.36764300
H	6.00680800	0.47078000	-3.13482000
H	5.68888400	1.88534300	-2.11152700
H	0.17189600	2.11235300	-0.58232100
H	-0.39656600	3.70255800	-1.16870300
H	-1.03196800	3.03240000	0.35301700
H	-2.64569300	3.97014500	-2.08882100
H	-2.40577300	1.13878100	2.10790000
H	-2.44503300	2.90803400	2.17807400
H	-3.28704700	1.96059100	3.41440400
H	-2.49511700	-3.11319200	1.45660900
H	-4.12025800	-3.84856400	1.43550000
H	-3.29301800	-3.46009200	-0.09839700
H	-4.99519700	0.01134400	-1.98046600
H	-6.74417000	0.28560600	-1.89449900
H	-5.69273600	1.50731000	-2.63212600
H	-7.54704100	2.37420400	-0.62067100
H	-5.26498700	3.41211700	2.87156700
H	-6.07474300	-2.61677200	0.74978200
H	-6.04087700	-0.89318900	0.33294000

H	-5.35399700	-2.10225300	-0.79481600
H	-3.38533600	-0.89406100	-2.45370400
H	-4.02396600	-2.28299400	-4.38369500
H	-2.65706400	-4.30738300	-4.95783300
H	-0.67905200	-4.87557700	-3.56804000
H	1.56404000	-3.60938000	-3.23518400
H	-0.64714900	-3.77952700	0.45082600
H	1.38289300	-4.56020200	1.63662500
H	3.60895500	-4.36764600	-2.04900500
H	3.52636800	-4.82927800	0.41049100
H	-3.86450400	2.68549500	-2.06650700
H	-3.34332600	3.44995900	-0.54398500
H	-2.24853900	1.31884200	-3.43373600
H	-1.12272100	2.70351600	-3.40153000
H	-0.56111700	1.08808100	-2.90628200
H	-4.82656500	-0.32481000	2.48091200
H	-4.88026400	-2.03461000	2.96603000
H	-3.31698300	-1.17612200	2.91414600
H	-7.31984400	4.73935400	2.20266100
H	-8.34053800	4.15917800	0.86012000
H	-8.30853100	3.28074600	2.40322500

6. Crystallographic Data

6.1 Compound S2

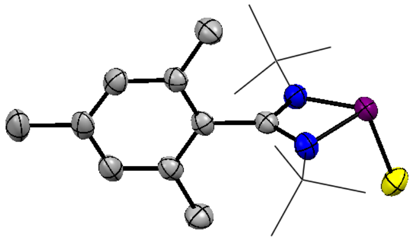

Empirical formula	$C_{18}H_{29}ClN_2Si$	
Formula weight	336.97	
Temperature	150(2) K	
Wavelength	1.54184 Å	
Crystal system	Triclinic	
Space group	P-1	
Unit cell dimensions	a = 8.8957(6) Å	α = 101.429(5)°.
	b = 9.1640(6) Å	β = 102.349(5)°.
	c = 12.6005(7) Å	γ = 95.939(6)°.
Volume	972.05(11) $Å^3$	
Z	2	
Density (calculated)	1.151 Mg/m^3	
Absorption coefficient	2.303 mm^{-1}	
F(000)	364	
Crystal size	0.350 x 0.190 x 0.130 mm^3	
Theta range for data collection	3.687 to 67.494°.	
Index ranges	-10<=h<=10, -10<=k<=10, -12<=l<=15	
Reflections collected	6294	
Independent reflections	3424 [R(int) = 0.0353]	
Completeness to theta = 67.494°	98.0 %	
Absorption correction	Semi-empirical from equivalents	
Max. and min. transmission	1.00000 and 0.65607	
Refinement method	Full-matrix least-squares on F^2	
Data / restraints / parameters	3424 / 0 / 227	
Goodness-of-fit on F^2	1.101	
Final R indices [I>2sigma(I)]	R1 = 0.0647, wR2 = 0.1760	
R indices (all data)	R1 = 0.0753, wR2 = 0.1828	
Largest diff. peak and hole	0.555 and -0.271 e.$Å^{-3}$	

6.2 Compound 2^{Mes}

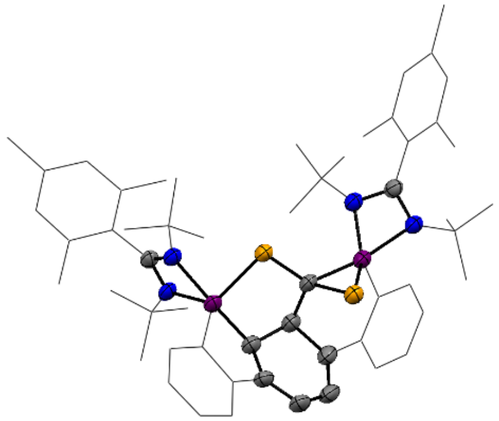

Empirical formula	$C_{69}H_{86}N_4S_2Si_2$ (including 2 toluene molecules)	
Formula weight	1091.71	
Temperature	150(2) K	
Wavelength	1.54184 Å	
Crystal system	Triclinic	
Space group	*P*-1	
Unit cell dimensions	a = 14.7039(9) Å	α = 104.274(5)°.
	b = 14.9136(8) Å	β = 95.168(5)°.
	c = 16.8768(11) Å	γ = 115.468(5)°.
Volume	3155.7(4) $Å^3$	
Z	2	
Density (calculated)	1.149 mg/m^3	
Absorption coefficient	1.447 mm^{-1}	
F(000)	1176	
Crystal size	0.120 x 0.100 x 0.040 mm^3	
Theta range for data collection	2.772 to 61.498°.	
Index range	-17<=h<=16, -17<=k<=17, -20<=l<=19	
Reflections collected	21271	
Independent reflections, R_{int}	11330 [R(int) = 0.0395]	
Completeness to theta = 61.498°	99.6 %	
Refinement method	Full-matrix least-squares on F^2	
Data / restraints / parameters	11330 / 0 / 714	
Goodness-of-fit on F^2, *S*	1.017	
Final R indices [I>2σ(I)]	R_1 = 0.0649, wR_2 = 0.1721	
R Indices (all data)	R_1 = 0.0897, wR_2 = 0.1958	
Largest diff. peak and hole	0.866 and -0.405 e.$Å^{-3}$	

6.3 Compound 3

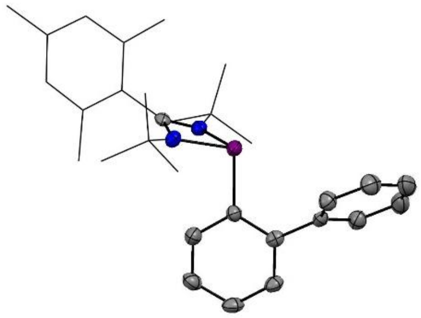

Empirical formula	$C_{54}H_4N_2Si$	
Formula weight	454.71	
Temperature	150(2) K	
Wavelength	1.54184 Å	
Crystal system	Orthorhombic	
Space group	$P2_12_12_1$	
Unit cell dimensions	a = 9.07710(10) Å	α = 90 °
	b = 14.34970(10) Å	β = 90 °
	c = 20.6941(2) Å	γ = 90 °
Volume	2695.48(4) $Å^3$	
Z	4	
Density (calculated)	1.120 Mg/m^3	
Absorption coefficient	0.896 mm^{-1}	
F(000)	984	
Crystal size	0.320 x 0.250 x 0.050 mm^3	
Theta range for data collection	3.748 bis 67.464°	
Index range	-10←h←10, -17←k←12, -24←l←24	
Reflections collected	18272	
Independent reflections, R_{int}	4829 [R_{int} = 0.0189]	
Completeness	99.8 %	
Absorption correction	semi empirical (*multi scan*)	
Max. and min. transmission	1.00000 und 0.76492	
Refinement method	Full-matrix least-squares on F^2	
Data / restraints / parameters	4829 / 0 / 307	
Goodness-of-fit on F^2, *S*	1.050	
Final R indices [I>2σ(I)]	R_1 = 0.0289, wR_2 = 0.0784	
R Indices (all data)	R_1 = 0.0295, wR_2 = 0.0812	
Largest diff. peak and hole	0.292 und -0.162 e.$Å^{-3}$	

6.4 Compound 4

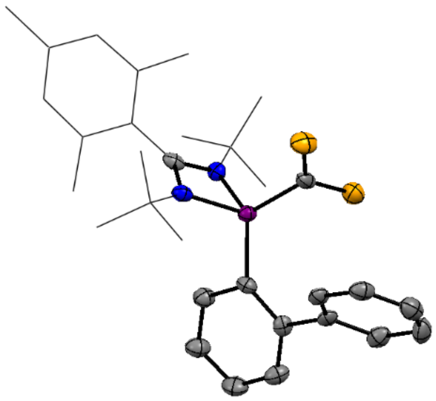

Empirical formula	$C_{34.50}H_{42}N_2S_2Si$ (including 0.5 toluene molecules)	
Formula weight	576.91	
Temperature	150(2) K	
Wavelength	1.54184 Å	
Crystal system	Monoclinic	
Space group	*P*c	
Unit cell dimensions	a = 10.97620(10) Å	α = 90 °
	b = 16.7272(2) Å	β = 102.2820(10)°
	c = 17.8380(2) Å	γ = 90 °
Volume	3200.12(6) $Å^3$	
Z	4	
Density (calculated)	1.197 Mg/m^3	
Absorption coefficient	2.048 mm^{-1}	
F(000)	1236	
Crystal size	0.100 x 0.050 x 0.030 mm^3	
Theta range for data collection	2.642 to 67.497°	
Index range	-13<=h<=13, -20<=k<=19, -21<=l<=21	
Reflections collected	21463	
Independent reflections, R_{int}	9179 [R_{int} = 0.0243]	
Completeness	99.5 %	
Absorption correction	semi empirical (*multi scan*)	
Max. und Min. Transmission	1.00000 and 0.62662	
Refinement method	Full-matrix least-squares on F^2	
Data / restraints / parameters	9179/ 101 / 731	
Goodness-of-fit on F^2, *S*	1.028	
Final R indices [I>2σ(I)]	R_1 = 0.0322 wR_2 = 0.0825	
R Indices (all data)	R_1 = 0.0380, wR_2 = 0.0886	
Largest diff. peak and hole	0-254 and -0.296 e.$Å^{-3}$	

6.5 Compound 6

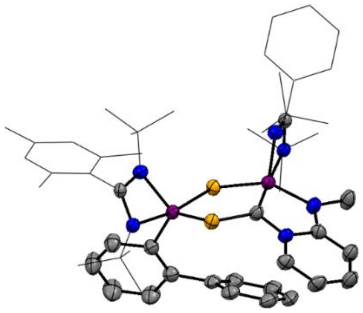

Empirical formula	$C_{52}H_{68}N_6S_2Si_2$	
Formula weight	897.42	
Temperature	150(2) K	
Wavelength	1.54184 Å	
Crystal system	Monoclinic	
Space group	$P2_1/n$	
Unit cell dimensions	a = 16.8842(5) Å	α = 90°.
	b = 17.6363(4) Å	β = 100.291(3)°.
	c = 17.9120(6) Å	γ = 90°.
Volume	5247.9(3) $Å^3$	
Z	4	
Density (calculated)	1.136 Mg/m^3	
Absorption coefficient	1.649 mm^{-1}	
F(000)	1928	
Crystal size	0.210 x 0.120 x 0.050 mm^3	
Theta range for data collection	3.314 to 67.497°.	
Index ranges	-20<=h<=17, -20<=k<=21, -21<=l<=21	
Reflections collected	36328	
Independent reflections, R_{int}	9441 [R_{int} = 0.0819]	
Completeness to theta = 67.497°	99.9 %	
Refinement method	Full-matrix least-squares on F^2	
Data / restraints / parameters	9441 / 0 / 575	
Goodness-of-fit on F^2, *S*	0.994	
Final R indices [I>2σ(I)]	R_1 = 0.0538, wR_2 = 0.1238	
R indices (all data)	R_1 = 0.0827, wR_2 = 0.1387	
Largest diff. peak and hole	0.399 and -0.266 e.$Å^{-3}$	

6.6 Compound 8

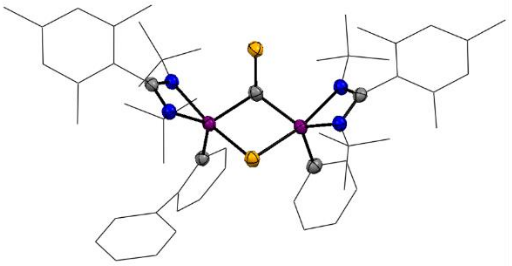

Empirical formula	$C_{73}H_{90}N_4S_2Si_2$ (including 3 benzene molecules)	
Formula weight	1143.78	
Temperature	150(2) K	
Wavelength	1.54184 Å	
Crystal system	Triclinic	
Space group	*P*-1	
Unit cell dimensions	a = 13.8712(5) Å	α = 82.195(4)°.
	b = 15.6328(7) Å	β = 87.552(3)°.
	c = 15.7624(7) Å	γ = 75.047(4)°.
Volume	3271.6(2) $Å^3$	
Z	2	
Density (calculated)	1.161 Mg/m^3	
Absorption coefficient	1.418 mm^{-1}	
F(000)	1232	
Crystal size	0.050 x 0.040 x 0.030 mm^3	
Theta range for data collection	2.830 to 67.499°.	
Index ranges	-14<=h<=16, -18<=k<=18, -18<=l<=18	
Reflections collected	22803	
Independent reflections, R_{int}	11766 [R_{int} = 0.0402]	
Completeness to theta = 67.499°	99.6 %	
Refinement method	Full-matrix least-squares on F^2	
Data / restraints / parameters	11766 / 0 / 748	
Goodness-of-fit on F^2, *S*	1.025	
Final R indices [I>2σ(I)]	R_1 = 0.0442, wR_2 = 0.1086	
R indices (all data)	R_1 = 0.0608, wR_2 = 0.1219	
Largest diff. peak and hole	0.330 and -0.400 e.$Å^{-3}$	

6.7 Compound 9

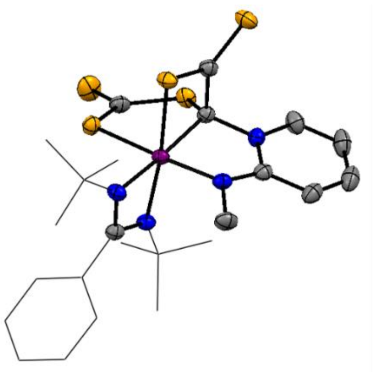

Empirical formula	$C_{31}H_{38}N_4S_5Si$ (including 1 toluene molecule)	
Formula weight	655.04	
Temperature	150(2) K	
Wavelength	1.54184 Å	
Crystal system	Triclinic	
Space group	*P*-1	
Unit cell dimensions	a = 10.4013(4) Å	α = 97.345(3)°.
	b = 11.3810(4) Å	β = 103.185(3)°.
	c = 14.4360(5) Å	γ = 96.635(3)°.
Volume	1631.56(10) Å^3	
Z	2	
Density (calculated)	1.333 Mg/m^2	
Absorption coefficient	3.840 mm^{-1}	
F(000)	692	
Crystal size	0.020 x 0.020 x 0.010 mm^3	
Theta range for data collection	3.185 to 67.496°.	
Index ranges	-12<=h<=12, -13<=k<=13, -17<=l<=10	
Reflections collected	11109	
Independent reflections, R_{int}	5884 [R_{int} = 0.0253]	
Completeness to theta = 67.496°	99.8 %	
Refinement method	Full-matrix least-squares on F	
Data / restraints / parameters	5884 / 0 / 378	
Goodness-of-fit on F^2, *S*	1.038	
Final R indices [I>2σ(I)]	R_1 = 0.0339, wR_2 = 0.0902	
R indices (all data)	R_1 = 0.0394, wR_2 = 0.0958	
Largest diff. peak and hole	0.432 and -0.309 e.Å^{-3}	

6.8 Compound 10

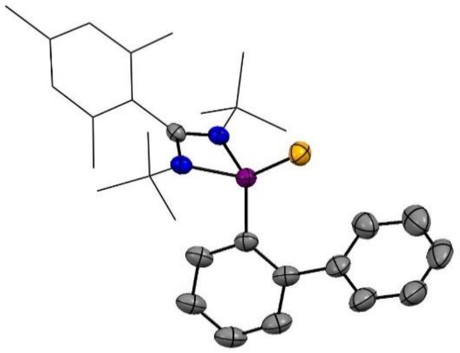

Empirical formula	$C_{30}H_{38}N_2SSi$	
Formula weight	486.77	
Temperature	150(2) K	
Wavelength	1.54184 Å	
Crystal system	Triclinic	
Space group	*P*-1	
Unit cell dimensions	a = 9.0115(3) Å	a= 102.785(3)°.
	b = 18.9015(7) Å	b= 90.166(3)°.
	c = 19.3421(8) Å	g = 99.367(3)°.
Volume	3167.4(2) Å^3	
Z	4	
Density (calculated)	1.021 Mg/m^3	
Absorption coefficient	1.390 mm^{-1}	
F(000)	1048	
Crystal size	0.080 x 0.040 x 0.020 mm^3	
Theta range for data collection	2.975 to 67.499°.	
Index ranges	-10<=h<=10, -21<=k<=22, -23<=l<=23	
Reflections collected	22180	
Independent reflections	11426 [R(int) = 0.0417]	
Completeness to theta = 67.499°	99.9 %	
Absorption correction	Semi-empirical from equivalents	
Max. and min. transmission	1.00000 and 0.40424	
Refinement method	Full-matrix least-squares on F^2	
Data / restraints / parameters	11426 / 0 / 631	
Goodness-of-fit on F^2	0.976	
Final R indices [I>2sigma(I)]	R1 = 0.0450, wR2 = 0.1119	
R indices (all data)	R1 = 0.0621, wR2 = 0.1216	
Extinction coefficient	n/a	
Largest diff. peak and hole	0.202 and -0.310 e.Å^{-3}	

7. References

[1] A. Velian, S. Lin, A. J. M. Miller, M. W. Day, T. Agapie, *J. Am. Chem. Soc.* **2010**, *132*, 6296.

[2] C.-W. So, H. W. Roesky, J. Magull, R. B. Oswald, *Angew. Chem.* **2006**, *118*, 4052; *Angew. Chem. Int. Ed.* **2006**, *45*, 3948.

[3] Z. Mo, T. Szilvási, Y.-P. Zhou, S. Yao, M. Driess, *Angew. Chem.* **2017**, *129*, 3753; *Angew. Chem. Int. Ed.* **2017**, *56*, 3699.

[4] X. Qi, T. Zheng, J. Zhou, Y. Dong, X. Zuo, X. Li, H. Sun, O. Fuhr, D. Fenske, *Organometallics* **2019**, *38*, 268.

[5] M.-P. Lücke, S. Yao, M. Driess, *Chem. Sci.* **2021**, *12*, 2909.

[6] G. M. Sheldrick, *SHELX-97 Program for Crystal Structure Determination, Universität Göttingen (Germany* ***1997****)*.

[7] Gaussian 09, Revision D.01, M. J. Frisch, G. W. Trucks, H. B. Schlegel, G. E. Scuseria, M. A. Robb, J. R. Cheeseman, G. Scalmani, V. Barone, B. Mennucci, G. A. Petersson, H. Nakatsuji, M. Caricato, X. Li, H. P. Hratchian, A. F. Izmaylov, J. Bloino, G. Zheng, J. L. Sonnenberg, M. Hada, M. Ehara, K. Toyota, R. Fukuda, J. Hasegawa, M. Ishida, T. Nakajima, Y. Honda, O. Kitao, H. Nakai, T. Vreven, J. A. Montgomery, Jr., J. E. Peralta, F. Ogliaro, M. Bearpark, J. J. Heyd, E. Brothers, K. N. Kudin, V. N. Staroverov, T. Keith, R. Kobayashi, J. Normand, K. Raghavachari, A. Rendell, J. C. Burant, S. S. Iyengar, J. Tomasi, M. Cossi, N. Rega, J. M. Millam, M. Klene, J. E. Knox, J. B. Cross, V. Bakken, C. Adamo, J. Jaramillo, R. Gomperts, R. E. Stratmann, O. Yazyev, A. J. Austin, R. Cammi, C. Pomelli, J. W. Ochterski, R. L. Martin, K. Morokuma, V. G. Zakrzewski, G. A. Voth, P. Salvador, J. J. Dannenberg, S. Dapprich, A. D. Daniels, O. Farkas, J. B. Foresman, J. V. Ortiz, J. Cioslowski, D. J. Fox, *Gaussian, Inc., Wallingford CT* **2013**.

[8] a) P. J. Stephens, F. J. Devlin, C. F. Chabalowski, M. J. Frisch, *J. Phys. Chem.* **1994**, *98*, 11623; b) S. H. Vosko, L. Wilk, M. Nusair, *Can. J. Phys.* **1980**, *58*, 1200; c) Lee, Yang, Parr, *Phys. Rev. B* **1988**, *37*, 785; d) A. D. Becke, *J. Chem. Phys.* **1993**, 5648.

[9] a) S. Grimme, S. Ehrlich, L. Goerigk, *J. Comput. Chem.* **2011**, *32*, 1456; b) S. Grimme, J. Antony, S. Ehrlich, H. Krieg, *J. Chem. Phys.* **2010**, *132*, 154104.

[10] F. Weigend, R. Ahlrichs, *Phys. Chem. Chem. Phys.* **2005**, *7*, 3297.

[11] E. D. Glendening, C. R. Landis, F. Weinhold, *J. Comput. Chem.* **2013**, *34*, 1429.

[12] Gaussian 16, Revision A.03, M. J. Frisch, G. W. Trucks, H. B. Schlegel, G. E. Scuseria, . A. Robb, J. R. Cheeseman, G. Scalmani, V. Barone, G. A. Petersson, H. Nakatsuji, X. Li, M. Caricato, A. V. Marenich, J. Bloino, B. G. Janesko, R. Gomperts, B. Mennucci, H. P. Hratchian, J. V. Ortiz, A. F. Izmaylov, J. L. Sonnenberg, D. Williams-Young, F. Ding, F. Lipparini, F. Egidi, J. Goings, B. Peng, A. Petrone, T. Henderson, D. Ranasinghe, V. G. Zakrzewski, J. Gao, N. Rega, G. Zheng, W. Liang, M. Hada, M. Ehara, K. Toyota, R. Fukuda, J. Hasegawa, M. Ishida, T. Nakajima, Y. Honda, O. Kitao, H. Nakai, T. Vreven, K. Throssell, J. A. Montgomery, Jr., J. E. Peralta, F. Ogliaro, M. J. Bearpark, J. J. Heyd, E. N. Brothers, K. N. Kudin, V. N. Staroverov, T. A. Keith, R. Kobayashi, J. Normand, K. Raghavachari, A. P. Rendell, J. C. Burant, S. S. Iyengar, J. Tomasi, M. Cossi, J. M. Millam, M. Klene, C. Adamo, R. Cammi, J. W. Ochterski, R. L. Martin, K. Morokuma, O. Farkas, J. B. Foresman, D. J. Fox, *Gaussian, Inc., Wallingford CT* **2016**.
